# A randomized clinical trial testing digital mindset intervention for knee osteoarthritis pain and activity improvement

**DOI:** 10.1038/s41746-024-01281-8

**Published:** 2024-10-17

**Authors:** Melissa A. Boswell, Kris M. Evans, Disha Ghandwani, Trevor Hastie, Sean R. Zion, Paula L. Moya, Nicholas J. Giori, Jennifer L. Hicks, Alia J. Crum, Scott L. Delp

**Affiliations:** 1 Joe Gibbs Human Performance Institute, Huntersville, NC 28078 USA; 2 Department of Bioengineering, Stanford University, Stanford, CA 94305 USA; 3 Department of Psychology, Stanford University, Stanford, CA 94305 USA; 4 Department of Statistics, Stanford University, Stanford, CA 94305 USA; 5 Department of English, Stanford University, Stanford, CA 94305 USA; 6 Department of Orthopedic Surgery, Stanford University, Stanford, CA 94305 USA; 7 Department of Mechanical Engineering, Stanford University, Stanford, CA 94305 USA

**Keywords:** Patient education, Human behaviour

## Abstract

This randomized clinical trial evaluated the effectiveness of short, digital interventions in improving physical activity and pain for individuals with knee osteoarthritis. We compared a digital mindset intervention, focusing on adaptive mindsets (e.g., osteoarthritis is manageable), to a digital education intervention and a no-intervention group. 408 participants with knee osteoarthritis completed the study online in the US. The mindset intervention significantly improved mindsets compared to both other groups (*P* < 0.001) and increased physical activity levels more than the no-intervention group (mean = 28.6 points, *P* = 0.001), but pain reduction was not significant. The mindset group also showed significantly greater improvements in the perceived need for surgery, self-imposed physical limitations, fear of movement, and self-efficacy than the no-intervention and education groups. This trial demonstrates the effectiveness of brief digital interventions in educating about osteoarthritis and further highlights the additional benefits of improving mindsets to transform patients’ approach to disease management. The study was prospectively registered (ClinicalTrials.gov: NCT05698368, 2023-01-26).

## Introduction

Knee osteoarthritis is a leading cause of disability^1^, with symptoms including pain, stiffness, and functional impairment^2^. While physical activity has a host of benefits for individuals with knee osteoarthritis, such as improved knee pain and functioning^3^, quality of life^4^, cartilage health^5^, and overall cost-saving^6^, physical activity levels in individuals with knee osteoarthritis are lower than those without osteoarthritis^7^. Psychosocial variables, such as beliefs about pain and fear of further joint damage, are significant contributors to lower physical activity engagement^8^.

While psychological interventions for osteoarthritis delivered in person or via live video sessions, such as cognitive behavioral therapy, can ameliorate pain^9–11^, loss of function^11–13^, and depression^11^, they are time- and resource-intensive. A digital approach would offer a scalable and potentially cost-effective alternative. However, no such knee osteoarthritis program has been evaluated in a clinical trial^14–17^.

One promising but underutilized approach in psychological interventions for individuals with knee osteoarthritis is fostering adaptive mindsets. Mindsets, or core assumptions about a conceptual domain, shape our expectations, attributions, and goals, helping us make sense of complex information and decide our actions^18^. Mindsets about the process of engaging in exercise (e.g., “exercise is difficult, boring, stressful, and isolating” versus “exercise is easy, fun, relaxing, and social”) predict physical activity levels in individuals with knee osteoarthritis^19^. But modifying mindsets in knee osteoarthritis patients and the subsequent impact on activity levels and knee symptoms remains unexplored.

In this study, we designed and tested a digital mindset intervention for knee osteoarthritis patients. Our primary hypothesis was that the mindset intervention would improve physical activity and pain more than no intervention. We also hypothesized that the mindset intervention would, as an additional benefit, foster more adaptive mindsets about exercise and osteoarthritis than an education intervention and no intervention. We secondarily evaluated changes in knee osteoarthritis knowledge, symptoms, perceived need for surgery, fear of movement, self-efficacy, and physical and mental health.

## Results

**Fig. 1:**
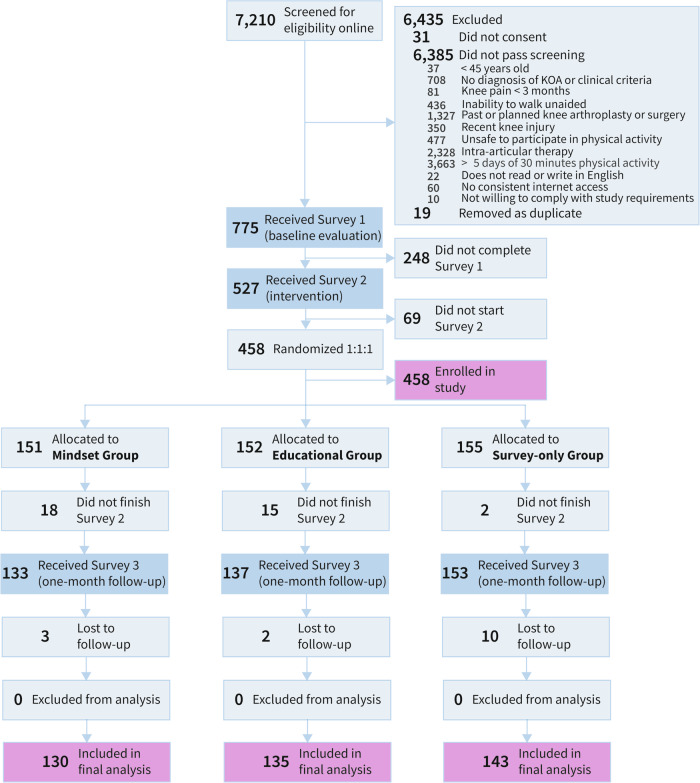
Study flow chart. The study flow chart depicts the progression of participants through the study, from initial screening to final analysis, including the number of participants at each stage and reasons for exclusion or dropout.

Of 7210 individuals who took the online screening survey, 775 qualified to participate in the study (Fig. 1). A total of 458 participants started Survey 2, were randomized, and enrolled in the study (Table 1). Outcomes were evaluated on the 408 people who completed Survey 3 (age, mean 63.6 [SD 8.8] years; *n* = 250 [61.3%] women). The average BMI of participants was 34.2 [8.5], which falls within the obese category. At baseline, the mindset intervention group reported less pain than the education and no-intervention groups (4.7 [1.9], 5.3 [1.7], 5.3 [1.6] points, respectively); however, after correcting for multiplicity, the differences were no longer considered significant. Thus, we did not control for any baseline differences across groups. A similar number of individuals completed the study across all three groups (mindset group: 86%, education group: 89%, no-intervention group: 92%). We found no significant differences between those who completed the study and those who withdrew after enrollment (Supplementary Table 8). None of the participants reported serious adverse events.

**Table 1:** Baseline descriptive statistics for participants who completed Survey 3 in each participant group

Characteristic	Mindset group *N* = 130	Education group *N* = 135	No-intervention group *N* = 143
Age, mean (SD), years	64.1 (8.0)	63.6 (9.2)	63.0 (9.0)
Sex, *n* (%)			
Male	51 (39.2)	54 (40.0)	58 (40.6)
Female	78 (60.0)	81 (60.0)	85 (59.4)
Nonbinary	1 (0.8)	0 (0.0)	0 (0.0)
Gender, *n* (%)			
Male	51 (39.2)	54 (40.0)	58 (40.6)
Female	77 (59.2)	81 (60.0)	85 (59.4)
Transgender male	1 (0.8)	0 (0.0)	0 (0.0)
Transgender female	1 (0.8)	0 (0.0)	0 (0.0)
Gender variant/non-conforming	0 (0.0)	0 (0.0)	0 (0.0)
Not listed	0 (0.0)	0 (0.0)	0 (0.0)
BMI, mean (SD), kg/m^2^	34.3 (10.0)	34.0 (7.9)	34.3 (8.2)
Race and Ethnicity (not mutually exclusive), *n* (%)			
American Indian or Alaskan Native	2 (1.5)	4 (3.0)	3 (2.1)
Asian	7 (5.4)	7 (5.2)	2 (1.4)
Black or African-American	4 (3.1)	6 (4.4)	9 (6.3)
Hispanic or Latino	2 (1.5)	2 (1.5)	10 (7.0)
Native Hawaiian or Pacific Islander	0 (0.0)	0 (0.0)	0 (0.0)
White	118 (90.8)	118 (87.4)	128 (89.5)
Other	4 (3.1)	3 (2.2)	1 (0.7)
Currently employed, *n* (%)	54 (41.5)	57 (42.2)	60 (42.2)
Comorbid conditions, *n* (%)			
Heart disease	9 (6.9)	13 (9.6)	13 (9.1)
High blood pressure	70 (53.8)	68 (50.4)	67 (46.9)
Lung disease	8 (6.2)	3 (2.2)	3 (2.1)
Diabetes	32 (24.6)	28 (20.7)	26 (18.2)
Ulcer or stomach disease	1 (0.8)	2 (1.5)	2 (1.4)
Kidney disease	8 (6.2)	10 (7.4)	7 (4.9)
Liver disease	2 (1.5)	4 (3.0)	2 (1.4)
Anemia or blood disease	4 (3.1)	4 (3.0)	2 (1.4)
Cancer	7 (5.4)	6 (4.4)	3 (2.1)
Depression	18 (13.8)	24 (17.8)	36 (25.2)
Back pain	47 (36.2)	49 (36.3)	54 (37.8)
Unilateral symptoms, *n* (%)	46 (35.4)	50 (37.0)	41 (28.7)
Time since pain started, mean (SD), years	12.0 (11.7)	10.4 (10.0)	12.6 (12.0)
Average pain in most painful knee, mean (SD), 0–10	4.7 (1.9)	5.3 (1.7)	5.3 (1.6)

*BMI* body mass index (calculated as weight in kilograms divided by height in meters squared).

### Primary outcomes

The digital mindset intervention improved all post-intervention primary outcome measures, including mindsets (Table 2) with large effect sizes, as well as follow-up knee pain and physical activity levels (Table 3) with small to medium effect sizes.

**Table 2:** Outcomes primarily evaluated at baseline and immediately following intervention (post-intervention) within and between groups

	Values, mean (SD); Changes, mean (95% CI)															
	Mindset group (1) *N* = 130				Education group (2) *N* = 140				No-intervention group (3) *N* = 155				Difference in change between groups			
	Baseline	Post-intervention	Within-group change	Cohen *d*	Baseline	Post-intervention	Within-group change	Cohen *d*	Baseline	Post-intervention	Within-group change	Cohen *d*	1 vs. 2	*P value*	1 vs. 3	*P value*
Primary mindsets																
The Process of Health Mindset – Exercise^a^	2.2 (0.5)	2.7 (0.5)	0.5 (0.4, 0.6)	1.04	2.2 (0.5)	2.5 (0.5)	0.3 (0.2, 0.3)	0.73	2.1 (0.5)	2.2 (0.5)	0.1 (0.0, 0.1)	0.30	0.2 (0.1, 0.3)	<0.001	0.4 (0.3, 0.5)	<0.001
Osteoarthritis is Castrophic Mindset^b^	3.9 (1.2)	2.8 (1.1)	−1.1 (−1.3, −1.0)	−1.13	4.1 (1.1)	3.6 (1.1)	−0.5 (−0.6, −0.3)	−0.63	4.1 (1.0)	3.9 (1.0)	−0.2 (−0.3, −0.1)	−0.24	−0.7 (−0.9, −0.5)	<0.001	−1.0 (−1.2, −0.7)	<0.001
Osteoarthritis is Manageable Mindset^b^	3.7 (0.8)	4.8 (0.8)	1.1 (0.9, 1.2)	1.19	3.7 (0.9)	4.3 (0.8)	0.7 (0.5, 0.8)	0.88	3.6 (0.9)	3.9 (0.9)	0.2 (0.1, 0.3)	0.42	0.4 (0.2, 0.6)	<0.001	0.8 (0.7, 1.0)	<0.001
Osteoarthritis is Opportunistic Mindset^b^	3.2 (0.8)	4.3 (0.9)	1.1 (1.0, 1.2)	1.35	3.2 (0.9)	3.9 (0.9)	0.7 (0.6, 0.8)	0.92	3.1 (0.8)	3.3 (0.8)	0.2 (0.0, 0.3)	0.27	0.5 (0.3, 0.7)	<0.001	1.0 (0.8, 1.1)	<0.001
Secondary mindsets																
Knee osteoarthritis knowledge^c^	33.3 (6.1)	43.8 (6.6)	10.5 (9.4, 11.6)	1.64	33.0 (5.9)	36.1 (5.5)	3.1 (2.2, 4.0)	0.59	32.8 (6.4)	33.4 (6.5)	0.6 (−0.1, 1.4)	0.15	7.4 (6.0, 8.8)	<0.001	9.9 (8.6, 11.2)	<0.001
The Body is Adversarial^d^	2.7 (1.0)	2.1 (1.0)	−0.6 (−0.8, −0.5)	−0.66	2.9 (1.0)	2.5 (1.0)	−0.4 (−0.5, −0.2)	−0.50	2.8 (1.0)	2.8 (1.0)	−0.1 (−0.1, 0.0)	0.10	−0.2 (−0.4, 0.0)	<0.001	−0.5 (−0.7, −0.4)	<0.001
The Body is Capable^d^	3.0 (0.8)	4.4 (0.9)	1.3 (1.2, 1.5)	1.32	3.1 (0.9)	3.7 (0.9)	0.6 (0.4, 0.7)	0.75	3.0 (0.9)	3.2 (0.9)	0.2 (0.0, 0.3)	0.22	0.8 (0.5, 1.0)	<0.001	1.2 (1.0, 1.4)	<0.001
The Body is Responsive^d^	4.1 (0.8)	4.8 (0.8)	0.7 (0.5, 0.8)	0.81	4.0 (0.9)	4.3 (0.9)	0.4 (0.2, 0.5)	0.47	4.0 (0.8)	4.1 (0.8)	0.2 (0.0, 0.2)	0.15	0.3 (0.1, 0.5)	<0.001	0.6 (0.4, 0.8)	<0.001
Adequacy of Activity Mindset^e^	3.0 (1.2)	3.3 (1.5)	0.3 (0.1, 0.5)	0.28	3.2 (1.3)	3.5 (1.5)	0.2 (0.1, 0.4)	0.28	2.9 (1.4)	3.0 (1.4)	0.0 (−0.1, 0.1)	0.04	0.1 (−0.2, 0.3)	0.288	0.3 (0.1, 0.5)	0.006

^a^The Process of Health Mindset – Exercise score range, 1 (low mindset) to 4 (high mindset).

^b^Osteoarthritis Mindsets score range, 1 (low mindset) to 6 (high mindset).

^c^Knee Osteoarthritis Knowledge range, 1 (low knowledge) to 55 (high knowledge).

^d^Body Mindsets score range, 1 (low mindset) to 6 (high mindset).

^e^Adequacy of Activity score range, 1 (low mindset) to 5 (high mindset).

**Table 3:** Outcomes primarily evaluated at baseline and one-month follow-up within and between groups

	Values, mean (SD); Changes, mean (95% CI)												Mean (95%CI)			
	Mindset group (1) *N* = 130				Education group (2) *N* = 135				No-intervention group (3) *N* = 143				Difference in change between groups			
	Baseline	One-month	Within-group change	Cohen *d*	Baseline	One-month	Within-group change	Cohen *d*	Baseline	One-month	Within-group change	Cohen *d*	1 vs. 2	*P value*	1 vs. 3	*P value*
Primary outcomes																
Knee pain^a^	4.7 (1.9)	4.3 (2.1)	−0.5 (−0.8, −0.1)	−0.25	5.3 (1.6)	5.1 (1.8)	−0.2 (−0.5, 0.0)	−0.17	5.2 (1.6)	5.1 (1.9)	−0.1 (−0.4, 0.2)	−0.07	−0.2 (−0.2, 0.6)	0.154	−0.3 (−0.7, 0.1)	0.054
Physical activity^b^	153.7 (88.8)	192.6 (106.4)	38.9 (24.3, 53.6)	0.46	157.2 (94.1)	189.8 (98.9)	32.6 (18.7, 46.3)	0.40	161.0 (109.5)	171.3 (113.5)	10.3 (−2.0, 22.6)	0.14	6.4 (−1.9, 22.6)	0.266	28.6 (24.2, 53.6)	0.001
Secondary outcomes																
Activity-related knee symptoms^c^																
Pain	7.9 (3.2)	7.1 (3.2)	−0.8 (−1.2, −0.4)	−0.37	8.6 (2.9)	8.1 (2.9)	−0.6 (−1.0, −0.2)	−0.27	8.7 (3.2)	8.3 (3.4)	−0.4 (−0.7, 0.0)	−0.17	−0.2 (−0.3, 0.8)	0.209	−0.4 (−1.0, 0.1)	0.049
Function	9.8 (4.9)	8.8 (5.4)	−1.0 (−1.5, −0.4)	−0.30	11.0 (4.3)	10.2 (4.5)	−0.7 (−1.3, −0.2)	−0.22	10.8 (4.2)	10.3 (4.9)	−0.5 (−1.1, 0.0)	−0.15	0.3 (−0.5, 1.1)	0.255	−0.5 (−1.3, 0.3)	0.118
Perceived need for surgery^d^	3.8 (1.1)	3.2 (1.3)	−0.6 (−0.7, −0.4)	−0.58	3.7 (1.1)	3.5 (1.3)	−0.2 (−0.4, 0,0)	−0.21	3.7 (1.2)	3.6 (1.2)	0.0 (−0.2, 0.1)	−0.06	−0.4 (−0.6, −0.1)	0.003	−0.5 (−0.7, −0.3)	<0.001
Symptom management, %																
Pain medication (1)	66.9 (47.2)	63.1 (48.4)	−3.8 (−12.5, 4.8)	0.08	66.7 (47.3)	71.1 (45.5)	4.4 (−0.1, 9.0)	0.16	71.3 (45.4)	62.2 (48.6)	−9.1 (−16.1, −2.1)	−0.21	−8.3 (−18.0, 1.4)	0.467	5.2 (−5.8, 16.3)	0.176
Injections (2)	29.2 (45.7)	23.1 (42.9)	−6.2 (−13.8, 1.5)	−0.14	28.9 (45.5)	32.6 (47.0)	3.7 (−4.1, 11.5)	0.08	37.8 (48.6)	38.5 (48.8)	0.7 (−5.9, 7.3)	0.02	−9.9 (−20.8, 1.1)	0.039	−6.8 (−16.9, 3.2)	0.091
Exercise (3)	63.8 (48.2)	84.6 (36.2)	20.8 (12.8, 28.7)	0.45	61.5 (48.8)	77.8 (41.7)	16.3 (7.2, 25.3)	0.30	60.8 (49.0)	67.1 (47.1)	6.3 (−1.5, 14.1)	0.13	4.5 (−7.6, 16.5)	0.233	14.5 (3.3, 25.6)	0.006
Supervised physical therapy (4)	26.9 (44.5)	24.6 (43.2)	−2.3 (−9.2, 4.6)	0.06	20.7 (40.7)	25.2 (43.6)	4.4 (−2.7, 11.5)	0.11	32.2 (46.9)	29.4 (45.7)	−2.8 (−10.3, 4.7)	0.06	−6.8 (−16.7, 3.1)	0.091	0.5 (−9.8, 10.8)	0.463
Rest (10)	80.0 (40.2)	78.5 (41.3)	−1.5 (−10.1, 7.0)	0.03	74.1 (44.0)	85.2 (35.7)	11.1 (3.2, 19.0)	0.24	82.5 (38.1)	81.1 (39.3)	−1.4 (−8.9, 6.1)	0.03	−12.6 (−24.3, −1.0)	0.016	−0.1 (−11.5, 11.2)	0.490
Imposing physical limitations (11)	74.6 (43.7)	55.4 (49.9)	−19.2 (−28.3, −10.1)	0.36	61.5 (48.8)	66.7 (47.3)	5.2 (−4.3, 14.7)	0.09	69.2 (46.3)	61.5 (48.8)	−7.7 (−16.8, 1.4)	0.14	−24.2 (−37.6, −11.2)	<0.001	−11.5 (−24.5, 1.4)	0.040
Diet or weight management (5)	54.5 (50.0)	72.3 (44.9)	17.7 (8.2, 2.7)	0.32	63.0 (48.5)	77.8 (41.7)	14.8 (7.0, 22.7)	0.32	51.7 (50.1)	62.9 (48.5)	11.2 (3.1, 19.2)	0.23	2.9 (−9.4, 15.1)	0.323	6.5 (−5.8, 18.8)	0.151
Self-soothing (6)	60.0 (49.2)	70.0 (46.0)	10.0 (1.0, 19.0)	0.19	71.9 (45.1)	72.6 (44.8)	0.7 (−8.6, 10.1)	0.01	70.6 (45.7)	68.5 (46.6)	−2.1 (−10.0, 5.8)	0.04	9.3 (−22.3, 37.5)	0.081	12.1 (0.1, 24.1)	0.024
Mindset (9)	47.7 (50.1)	55.4 (49.9)	7.7 (−1.5, 16.9)	0.14	44.4 (49.9)	42.2 (50.0)	−2.2 (−11.3, 6.9)	0.04	41.3 (49.4)	35.0 (47.9)	−6.3 (−14.8, 2.2)	0.12	9.9 (−3.0, 22.9)	0.067	14.9 (1.4, 26.5)	0.014
Nothing (7)	3.1 (17.3)	3.1 (17.3)	0 (−4.3, 4.3)	0	3.7 (19.0)	3.0 (17.0)	−.7 (−5.1, 3.6)	0.03	4.2 (20.1)	3.0 (14.4)	−2.1 (−5.2, 1.0)	0.11	0.7 (−5.4, 6.9)	0.406	2.1 (−3.1, 7.3)	0.214
Physical health^e^	3.2 (0.6)	3.4 (0.6)	0.2 (0.1, 0.2)	0.40	3.1 (0.6)	3.2 (0.6)	0.1 (0.0, 0.2)	0.29	3.1 (0.6)	3.1 (0.6)	0.0 (0.0, 0.1)	0.04	0 (0.0, 0.1)	0.169	0.1 (0.0, 0.2)	0.001
Mental health^f^	3.5 (0.9)	3.6 (0.9)	0.1 (0.0, 0.2)	0.17	3.4 (0.8)	3.5 (0.0, 0.1)	0.0 (0.5)	0.10	3.4 (0.9)	3.3 (0.9)	0.0 (−0.1, 0.06)	−0.04	0 (0.0, 0.1)	0.288	0.1 (0.0, 0.2)	0.043
Fear of movement^g^	2.3 (0.7)	1.9 (0.7)	−0.4 (−0.5, −0.3)	−0.68	2.4 (0.7)	2.1 (−0.3, −0.1)	−0.2 (0.5)	−0.43	2.4 (0.6)	2.4 (0.7)	0.0 (0.0, 0.1)	0.04	−0.2 (−0.3, 0.0)	0.011	−0.4 (−0.5, −0.3)	<0.001
Arthritis self-efficacy^h^																
Pain	5.8 (2.2)	7.2 (1.9)	1.4 (1.1, 1.7)	0.78	6.1 (2.0)	6.7 (2.1)	0.6 (0.4, 0.9)	0.43	5.5 (2.1)	5.8 (2.3)	0.2 (0.0, 0.5)	0.17	0.7 (0.3, 1.1)	<0.001	1.1 (0.7, 1.5)	<0.001
Other symptoms	5.9 (2.1)	7.0 (2.1)	1.1 (0.9, 1.4)	0.72	6.1 (1.9)	6.7 (2.2)	0.6 (0.3, 0.9)	0.37	5.5 (2.2)	6.1 (2.3)	0.5 (0.3, 0.8)	0.34	0.5 (0.2, 0.9)	0.002	0.6 (0.2, 1.0)	<0.001

^a^NRS pain score range, 0 (no pain) to 10 (worst pain).

^b^Physical Activity Scale for the Elderly score range, 0 (no physical activity) to 793 (very high physical activity).

^c^Short version of the Western Ontario and McMaster Universities Arthritis Index; Pain subscore range, 0 (low pain) to 20 (high pain); Function subscore range, 0 (low difficulty) to 28 (high difficulty).

^d^Perceived need for surgery score range, 1 (very unlikely) to 5 (very likely).

^e^PROMIS v.1.1 Global Health Short Form Physical Health subscore range, 1 (poor) to 5 (excellent).

^f^PROMIS v.1.1 Global Health Short Form Mental Health subscore range, 1 (poor) to 5 (excellent).

^g^Brief Fear of Movement Scale for Osteoarthritis score range, 1 (low fear) to 4 (high fear)

^h^Arthritis-Self Efficacy Scale score range, 1 (low efficacy) to 10 (high efficacy).

The exercise mindset measure improved from baseline to post-intervention in the mindset group (mean change, 0.5 [95% CI, 0.4–0.6]; *d* = 1.04). The mindset group showed a significantly greater increase in the exercise mindset compared to the education group (between-group difference, 0.2 [0.1, 0.3], *P* < 0.001; Table 2) and the no-intervention group (0.4 [0.3, 0.5], *P* < 0.001).

The osteoarthritis mindset measures also improved in the mindset group (Osteoarthritis is a Catastrophe Mindset: −1.1 [−1.3, −1.0]; *d* = −1.13; Osteoarthritis is Manageable Mindset: 1.1 [0.9, 1.2] *d* = 1.19; Osteoarthritis is an Opportunity Mindset: 1.1 [1.0, 1.2], *d* = 1.35). The mindset group showed a significantly greater improvement in osteoarthritis mindsets compared to the education group (Catastrophe: −0.7 [−0.9, −0.5], *P* < 0.001; Manageable: 0.4 [0.2, 0.6], *P* < 0.001; Opportunity: 0.5 [0.3, 0.7], *P* < 0.001) and the no-intervention group (Catastrophe: −1.0 [−1.2, −0.7], *P* < 0.001; Manageable: 0.8 [0.7, 1.0], *P* < 0.001; Opportunity: 1.0 [0.8, 1.1], *P* < 0.001).

Physical activity levels increased from baseline to one-month follow-up in the mindset group (38.9 [24.3, 53.6]; *d* = 0.46). The mindset group showed a significantly greater increase in physical activity compared to the no-intervention group (28.6 [24.2, 53.6], *P* = 0.001). The increase in activity levels in the mindset group was not significantly greater than in the education group (6.4 [−1.9, 22.6], *P* = 0.266).

Knee pain decreased from baseline to one-month follow-up in the mindset group (−0.5 [−0.8, −0.1]; *d* = −0.25). The difference in change in pain between the mindset group and the no-intervention group was close to significance (−0.3 [−0.7, 0.1], *P* = 0.054). Decreases in pain in the mindset group were not significantly greater than in the education group (−0.2 [−0.2, 0.6], *P* = 0.154).

The education group also showed improvements from baseline in the exercise mindset (*d* = 0.73), osteoarthritis mindsets (|*d*| = 0.63–0.92), and physical activity (*d* = 0.40), but not in knee pain (*d* = −0.17; Tables 2 and 3).

When we performed Bonferroni correction for our four primary statistical comparisons at follow-up (changes in pain and physical activity compared across mindset vs. education and mindset vs. no intervention), we adjusted the significance threshold to *P* = 0.0125. In this case, the interpretation of the between-group changes in the primary variables does not change.

### Secondary outcomes

The mindset group improved in all secondary outcomes from baseline to post-intervention (Table 2) and baseline to one-month follow-up (Table 3).

Compared to the no-intervention group, the mindset group showed significantly greater improvements in activity-related knee pain, physical and mental health, fear of movement, pain self-efficacy, other symptoms self-efficacy, reduced perceived need for surgery, knee osteoarthritis knowledge, and body mindsets (Tables 2 and 3). The mindset group also had a significantly greater increase in the number of individuals who reported using exercise, self-soothing, and their mindset and a greater decrease in individuals who reported imposing physical limitations as symptom management strategies compared to the no-intervention group.

Compared to the education group, the mindset group had significantly greater improvements in fear of movement, pain self-efficacy, other symptoms self-efficacy, knee osteoarthritis knowledge, and body mindsets, and reduced perceived need for surgery (Tables 2 and 3). The mindset group also had a significantly greater decrease in individuals who reported using injections, imposing physical limitations, and rest as symptom management strategies compared to the education group.

### Qualitative feedback

The mindset and education groups generally valued arthritis management information, responded positively to exercise emphasis, and sought specific action plans (Supplementary Tables 9 and 10). The education group expressed concerns about technical complexity and dissatisfaction with the lack of an osteoarthritis cure. Participants in the mindset group noted an increased motivation for physical activity and new perspectives on exercise. For example, one participant stated, “I realize that exercise will not further damage my joints, and by changing my mindset, I can exercise more and improve my strength, which will better support my joints.” Another expressed, “I find that I do enjoy exercise more when I stay mindful of how it makes me feel happier in general and how it empowers me to be able to take better care of my body.”

## Discussion

This study tested a novel digital mindset intervention for individuals with knee osteoarthritis, representing the first clinical trial to assess a short and entirely self-guided online intervention for this population. The intervention improved mindsets and physical activity within the intervention group and compared to a no-intervention control group, highlighting its potential as an effective, inexpensive, and scalable intervention for improving physical activity, mindsets, and symptom management in patients with knee osteoarthritis.

The increase in physical activity measured by the Physical Activity Scale for the Elderly within the mindset intervention group was twice as large as improvements found after a six-month physical therapy plus at-home exercise program with coaching^20^ (39 vs. 18 points on the Physical Activity Scale for the Elderly, respectively). The 29-point difference in the Physical Activity Scale for the Elderly between the mindset group and the no-intervention group met our 26-point goal, corresponding to increases in both walking and light exercise from “seldom” for <1 h per day to “sometimes” for 1–2 h per day. The mindset intervention also reduced pain, although the decrease was below the minimum clinically important difference^21^, potentially due to the short follow-up time.

Our findings underscore the importance of addressing mindsets in individuals with knee osteoarthritis, aligning with research in other domains, such as diet^22^, physical activity^23^, and stress^24^, and demonstrating that changes in mindset can lead to self-fulfilling effects. Unlike approaches that aim to convince individuals that a particular mindset is true, our intervention focused on making participants aware of their mindsets and their impact, empowering them to adopt more adaptive perspectives. This metacognitive approach proved effective in motivating individuals to change their mindset about knee osteoarthritis and exercise, even in the face of challenges (i.e., knee pain and stiffness), leading to positive behavioral changes. Furthermore, this study contributes to the current gap in digital interventions attempting to augment self-beliefs^25^.

A key strength of our study was the inclusion of an active comparison (education intervention) to assess the unique contribution of explicitly targeting mindset in a digital intervention. We observed benefits from the digital education intervention compared to no intervention, highlighting the value of sharing evidence-based online content about knee osteoarthritis and its management. Moreover, the mindset intervention resulted in additional benefits, such as increased arthritis self-efficacy and reduced fear of movement and perceived need for surgery. These findings align with previous research showing that educational interventions focusing on empowerment and participatory discourse can improve self-efficacy and fear of movement^26^. These outcomes are crucial for individuals with knee osteoarthritis, as a lower perception of requiring surgery can lead to significant reductions in healthcare costs associated with osteoarthritis^27^. Additionally, higher levels of self-efficacy predict reductions in pain and higher levels of physical activity over longer periods^28^, while fear of movement contributes to physical disability^29^.

The study’s generalizability may be limited by the voluntary nature of participation and the recruitment format, which targeted individuals with social media accounts. We acknowledge that our sample was predominantly white (90%), which limits the generalizability of our findings to more diverse populations. The demographics of our sample, including high average BMI, predominately white participants, and low employment rate (40%) despite pre-retirement age, highlight the need for future research to include more diverse populations and consider the broader impacts of osteoarthritis on individuals’ lives. Another limitation of this study is the one-month follow-up duration; longer follow-ups are needed to assess the durability of physical activity and pain improvements. Additionally, physical activity was self-reported rather than objectively measured. Future studies could evaluate how baseline characteristics and behaviors relate to the impact of mindset interventions, optimizing interventions for specific sub-groups within the knee osteoarthritis population. While our study did not include a formal cost analysis, future work should also assess the cost-effectiveness of this digital intervention compared to standard care and other osteoarthritis management strategies. Finally, participant blinding was not possible; however, informing patients of their participation in a mindset or education intervention could be considered part of the intervention itself, as this would likely occur in real-world settings. The digital, self-directed format enhances scalability and accessibility, warranting exploration of its effectiveness in diverse populations and healthcare settings.

While our protocol was originally registered as a double-blinded study, in practice, participants were aware of their group assignment (as described in Randomization and Blinding). Two outcomes, self-fulfilling meta-mindset and controllable meta-mindset, were registered as secondary rather than as measures intended for exploratory analysis and, thus, were not included in this analysis.

Our randomized controlled trial shows that a digital mindset intervention effectively enhances mindsets and increases physical activity in individuals with knee osteoarthritis compared to treatment as usual. The intervention also improves psychological outcomes beyond an education intervention. Future research could explore synergies of the mindset intervention with complementary behavioral strategies, such as structured exercise and behavioral feedback, to further enhance patient engagement and knee osteoarthritis management outcomes. This study underscores the potential of *Rethinking Osteoarthritis* as an accessible tool for improving knee osteoarthritis management.

## Methods

We designed this three-arm, parallel groups, randomized controlled trial (prospectively registered on ClinicalTrials.gov on 2023-01-26, NCT05698368) per the SPIRIT guidelines for randomized controlled protocols (Supplementary Tables 1, 2 and Supplementary Figs. 1–5)^30^. We reported the trial according to the Consolidated Standards of Reporting Trials (CONSORT) Outcomes 2022 Checklist^31^ (Supplementary Table 3). The Stanford University Institutional Review Board approved this study (Protocol ID: 69227).

### Study design and recruitment

We recruited participants via social media (Supplementary Fig. 6), flyers (Supplementary Fig. 7), email (Supplementary Note 2), and word of mouth. Interested individuals accessed study information and provided electronic informed consent (Supplementary Note 1) on REDCap^32^. After passing a qualification questionnaire, participants completed a baseline evaluation (Survey 1) one to two weeks later (Supplementary Note 3). A week after Survey 1, participants received a link to Survey 2. After reading the introductory text of Survey 2 and choosing to continue, participants were immediately randomized using a randomizer function to one of three conditions: mindset intervention, education intervention, or no intervention. The assigned condition was then presented within the survey. Participants were considered enrolled upon randomization. One month later, participants received a follow-up survey (Survey 3). All surveys and interventions were delivered online (Qualtrics, Provo, UT, USA), and all questions required a response.

### Participants

Individuals 45 years or older qualified for the study if they self-reported a clinical diagnosis of knee osteoarthritis or met clinical criteria^33^. We required that patients were not regularly physically active or undergoing other significant knee osteoarthritis treatments.

### Randomization and blinding

We randomly assigned participants (1:1:1) to each group. The consent form shared with participants that the purpose of the study was to evaluate a new online osteoarthritis program and that they would be randomized into one of three groups. After randomization, the survey informed participants of their group assignment (i.e., participants were not blinded) but not about the specific nature of the other groups. The research team was aware of the condition assignment but had minimal interaction with participants, which was primarily done through automated online surveys. The biostatistician performed data analysis blind to group labels.

### Rethinking Osteoarthritis mindset intervention

**Fig. 2:**
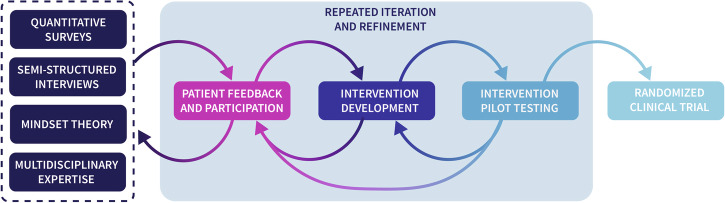
Mixed-methods approach for Rethinking Osteoarthritis. The mixed-methods approach to develop, pilot test, refine, and evaluate *Rethinking Osteoarthritis*, showcasing the integration of various research methods and feedback loops.

We deployed an iterative, mixed-methods approach to develop (Supplementary Note 4, Supplementary Table 4, and Supplementary Figs. 8, 9), pilot (Supplementary Note 5, Supplementary Tables 5–7, and Supplementary Fig. 10), and refine the digital mindset intervention, *Rethinking Osteoarthritis* (Fig. 2). This process incorporated mindset theory (Supplementary Fig. 9) and multidisciplinary expertise (i.e., bioengineering, psychology, narratology, and orthopedics). Knee osteoarthritis patients gave feedback throughout the design process and participated in the intervention videos.

**Fig. 3:**
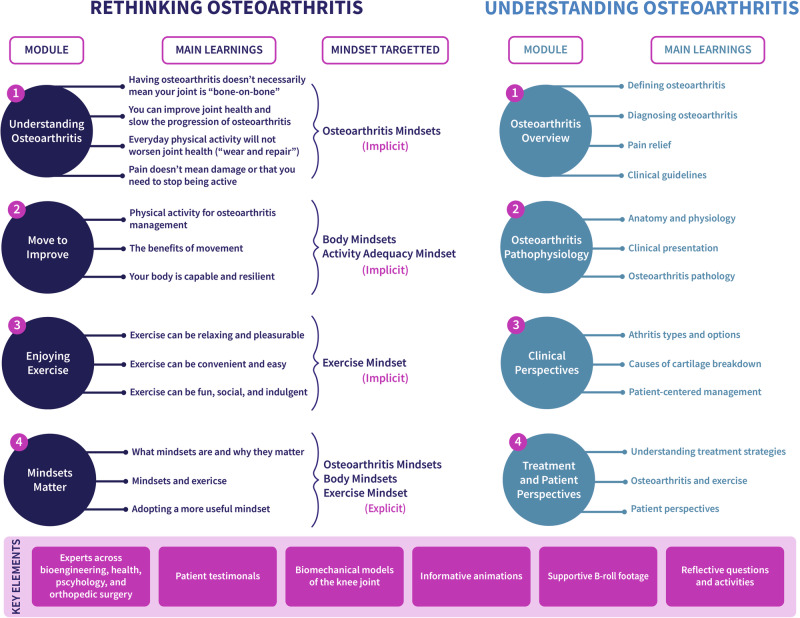
Comparison of intervention modules and their key elements. The modules of Rethinking Osteoarthritis (mindset intervention, left) and Understanding Osteoarthritis (education intervention, right) are paired with their associated main learnings and, if applicable, targeted mindsets. Below the targeted mindset indicates whether the module aimed to change the mindset implicitly or explicitly called out mindsets. Key intervention elements (bottom) exist throughout both interventions. Modules consist of videos and reflective questions featuring topic experts, interviews with individuals with osteoarthritis, and supplemental animations and b-roll footage. Both interventions total approximately 1 hour of videos and 30–60 minutes of reflective questions.

*Rethinking Osteoarthritis* has four modules, each featuring short films and reflective activities (Fig. 3; additional details in Supplementary Note 6). The videos, narrated by subject-matter experts, provide education on knee osteoarthritis and misconceptions surrounding it^34^, the benefits of exercise, and mindsets about osteoarthritis and physical activity. Reflective questions helped individuals think deeply about the content in the context of their lives, such as, “What mindset do you think would be helpful in overcoming your challenges with osteoarthritis and why?” Each module is approximately 10–20 minutes, with a total intervention length of less than 2 h. Participants had one week to complete the program at their own pace.

### Understanding Osteoarthritis education intervention

As an active comparison education intervention, we developed *Understanding Osteoarthritis* (Fig. 3; additional details in Supplementary Note 7)*. Understanding Osteoarthritis* consisted of educational videos and reflective questions of the same duration and required attention as *Rethinking Osteoarthritis*. We sourced YouTube^35–42^ videos on topics such as osteoarthritis pathology, risks, symptoms, and treatment strategies. The educational videos were verified for factual content and similarity to the mindset intervention videos, including teaching from live experts and patient testimonials.

### No intervention

This group completed the same surveys simultaneously as the other groups but received no additional video content or intervention-based reflective questions. We expected some benefit to participants in the education group; thus, the no-intervention group provided a comparison to quantify this effect.

### Data collection

Participants received $10 electronic gift cards after completing Surveys 1 and 2 and a $30 gift card after completing Survey 3. We reminded participants to complete the surveys via email, with up to three reminder emails to complete their surveys. Data collection was completed in September 2023.

### Participant characteristics

Survey 1 collected age, sex, gender, body mass index (BMI) from height and weight, race and ethnicity, education, employment, comorbid conditions, unilateral vs. bilateral osteoarthritis presentation, the time since knee pain started, and the time since knee osteoarthritis diagnosis.

### Primary outcomes

The primary outcomes assessed at the end of Survey 2 (post-intervention) included Exercise and Osteoarthritis mindsets. Exercise mindset was measured using the Mindset about the Process of Health – Exercise scale. This one-factor, 4-point scale was developed and validated^43^ to assess mindset about the process of engaging in physical activity (e.g., physical activity is difficult/easy, unpleasant/pleasurable, boring/fun), with a higher score reflecting a more appeal-focused mindset about exercise. Osteoarthritis mindsets were assessed using the Illness Mindset Inventory, which measures three mindsets about the nature and meaning of illness: that it is a catastrophe, manageable, or an opportunity, each on a 6-point scale, with a higher score indicating greater agreement with the mindset. This Illness Mindset Inventory is valid and reliable in individuals with knee osteoarthritis^44^. We adapted the Illness Mindset Inventory to focus on mindsets about “knee osteoarthritis” as opposed to “chronic disease.”

The primary outcomes assessed at one-month follow-up included knee pain and physical activity. Knee pain was assessed using the question, “*What was your average osteoarthritis-related pain over the past week?*” and measured on an 11-point Numeric Rating Scale from 0 (no pain at all) to 10 (the worst pain imaginable). We assessed physical activity using the Physical Activity Scale for the Elderly^45^. The scale is scored from 0 to 793, with higher scores indicating higher physical activity levels.

### Secondary outcomes

The secondary outcomes assessed post-intervention included the Knee Osteoarthritis Knowledge Scale^46^, the Illness Mindset Inventory for body mindsets^44^, and the Adequacy of Activity Mindset Measure^47^. The secondary outcomes assessed at one-month follow-up included (1) the knee pain and function subscales of the shortened version of the Western Ontario and McMaster Universities Arthritis Index, (2) the perceived need for surgery^26^, (3) chosen symptom management strategies^19^, (4) the Brief Fear of Movement Scale for Osteoarthritis^48^, (5) the pain and other symptoms subscales of the Arthritis-Self Efficacy Scale^49^, and (6) the physical and mental health subscales of the Patient-Reported Outcomes Measurement Information System v.1.1 Global Health Short Form^50^.

At the end of the study, we prompted qualitative insight as an exploratory measure: “We would like to learn more about your own experience with knee osteoarthritis and exercise since participating in this study. In your own words, please share your experience with knee osteoarthritis and exercise over the past three weeks.” Responses were reviewed to identify illustrative examples that contextualize the quantitative findings.

Additional details on measures are on ClinicalTrials.gov (NCT05698368).

### Sample size

The mindset intervention was considered beneficial if we found improvements in one or both of the primary outcomes of pain or physical activity level. An a priori power analysis was performed at a 5% significance level and effect size of 0.3 to achieve 80% power in detecting an improvement of 26 points in the Physical Activity Scale for the Elderly (additional details in the Supplementary Information – SPIRIT checklist). This analysis led to a sample size of 139 per group (417 total), which was adequately powered to detect a clinically relevant difference of 2 points in NRS pain^14^. We aimed to recruit 501 individuals to account for a 20% dropout rate.

### Statistical analysis

An author blinded to group assignment (D.G.) completed all analyses in R (v4.3.2)^51^. We summarized baseline characteristics with descriptive statistics. We used independent sample t-tests for continuous variables and Pearson χ2 or exact tests for categorical variables to test baseline group differences. We evaluated mean change scores within groups along with Cohen *d* (Eq. (1); where $\bar{{x}_{2}}$ denotes the mean of variable after intervention, $\bar{{x}_{1}}$ denotes the mean of variable before intervention, and $s$ denotes the standard deviation of the change in the variable due to intervention):

1 $${cohen\,d}=\frac{\bar{{x}_{2}}-\bar{{x}_{1}}}{s}$$

We evaluated mean differences in change scores between the groups using independent t-tests (Eq. (2); where $\bar{X}$ is the mean of changes in the first group and $\bar{Y}$ is the mean of the changes in the second group, ${s}_{{pooled}}$ is the pooled standard deviation from the two groups):

2 $$\frac{\bar{X}\,-\,\bar{Y}}{{s}_{{pooled}}\sqrt{\frac{1}{m}+\,\frac{1}{n}}}$$

We present the results with accompanying 95% confidence intervals (CIs) and *P* values, as well as with and without Bonferroni correction. Our analysis included only participants who completed all surveys in full, as each survey question required a response, ensuring no missing data within completed surveys.

## Supplementary information

Supplementary Information

## Data Availability

All data generated in this study are available open source on GitHub: https://github.com/melboswell/KOA-mindset-RCT/.

## Code availability

The custom R scripts used for data processing and analysis in this study are available open source on GitHub: https://github.com/melboswell/KOA-mindset-RCT/.
