# Supplementary material for: A randomized clinical trial testing digital mindset intervention for knee osteoarthritis pain and activity improvement
Source: NPJ Digit Med. 2024 Oct 17;7:285. doi: 10.1038/s41746-024-01281-8 (PMC11484881; doi:10.1038/s41746-024-01281-8)
Supplement: Supplementary file 1 — Supplementary Information [file 41746_2024_1281_MOESM1_ESM.pdf]

Supplementary Information

A Randomized Clinical Trial Testing Digital Mindset Intervention for Knee Osteoarthritis Pain and Activity Improvement

	Page
Trial Protocol and Consort Checklist	**3**
**Supplementary Table 1.** Study meta-data.	4
**Supplementary Table 2.** Study objectives.	6
**Supplementary Figure 1.** Study flow and timeline.	12
**Supplementary Figure 2.** Baseline vs. Follow-up PASE Scores with Difference Distribution.	15
**Supplementary Figure 3.** A Priori Sample Size Analysis for Outcome Variable PASE.	16
**Supplementary Figure 4.** Baseline vs. Follow-up Pain Scores with Change Distribution	17
**Supplementary Figure 5.** A Priori Sample Size Analysis for Outcome Variable Pain.	18
**Supplementary Table 3.** CONSORT Checklist.	23
**Supplementary Note 1.** Informed consent materials.	29
Recruitment Content	**38**
**Supplementary Figure 6.** Social media and online advertisement image.	38
**Supplementary Figure 7.** Printed newspaper advertisement image.	39

Supplementary Note 2. Recruitment email content. 40

Participant Email Communications **41**

Supplementary Note 3. Participant email communication content. 41

Intervention Design **47**

Supplementary Note 4. Interview guide 47

Supplementary Table 4. Participant characteristics of the semi-structured one-on-one interviews (N=10). 50

Supplementary Figure 8. Emergent themes from the semi-structured one-on-one interviews. 51

Supplementary Figure 9. A diagram theorizing osteoarthritis and exercise mindsets. 53

Intervention Pilot Testing

Supplementary Note 5. Open-ended feedback questions following each module and the entire program during the pilot study. 55

Supplementary Table 5. Participant characteristics of the semi-structured one-on-one interviews (N=21). 56

Supplementary Figure 10. Box-and-whisker plots of scores before (Pre) to after (Post) participating in the mindset intervention (N=21), as determined by a t-test. 57

Supplementary Table 6. Summary measures and estimated mean difference in change [95% CI]. 58

Supplementary Table 7. Summary scores of the program reaction and motivation-focused questions. 59

Intervention Content **61**

Supplementary Note 6. Mindset intervention content. 61

Supplementary Note 7. Educational intervention content. 76

Supplementary Results and Data **92**

Supplementary Table 8. Baseline descriptive statistics for participants who completed the study or withdrew or did not complete the study. 92

Supplementary Table 9. Three of the open-ended responses to *Rethinking Osteoarthritis* at the end of the program. 94

Supplementary Table 10. Three of the open-ended responses to *Understanding Osteoarthritis* at the end of the program. 107

Trial Protocol and Consort Checklist.

Protocol: A Digital Mindset Intervention to Improve Pain and Exercise Participation in Individuals With Knee Osteoarthritis: A Randomized Clinical Trial

SPIRIT Checklist

Investigators

Melissa Boswell
Kris Evans
Disha Ghandwani
Trevor Hastie
Sean Zion
Paula Moya
Nick Giori
Jennifer Hicks
Alia Crum
Scott Delp

Background and Significance

Osteoarthritis affects 7% of the global population and is a leading cause of disability globally. Physical activity improves health outcomes, weight management, and knee function for people with knee osteoarthritis and should be considered first-line treatment. Yet, physical activity levels in this population are low compared to those without knee osteoarthritis.

Emerging research has highlighted the powerful influence of mindsets about exercise on engagement in physical activity. Mindsets are core assumptions about a domain or category that orient individuals to a particular set of attributions, expectations, and

goals (a "meaning system"). In individuals with knee osteoarthritis, mindsets about the appeal of physical activity relate to future physical activity levels and one's chosen symptom management strategy, and mindsets about osteoarthritis relate to knee symptoms.

We developed a digital mindset intervention to improve mindsets about exercise and osteoarthritis in individuals with knee osteoarthritis. We piloted the intervention on 21 individuals with knee osteoarthritis throughout the United States. Participants improved in exercise and osteoarthritis mindsets. However, this was a small sample size, a control group was not used, and it was cross-sectional; thus, not able to evaluate changes in physical activity and osteoarthritis symptoms. A large randomized trial is therefore needed to evaluate if our mindset intervention leads to improvements in physical activity levels and osteoarthritis symptoms and, further, if these changes are due to more adaptive mindsets about exercise and osteoarthritis.

Supplementary Table 1. Study meta-data.

**Data category**	**Information**
Primary registry and trial identifying number	ClinicalTrials.gov: NCT05698368
Date of registration in primary registry	January 2023
Secondary identifying numbers	IRB-69227

Source(s) of monetary or material support	The Wu Tsai Human Performance Alliance at Stanford University and the Joe and Clara Tsai Foundation. The Stanford Catalyst for Collaborative Solutions; the Mobilize Center, which is supported by the National Institute of Biomedical Imaging and Bioengineering (NIBIB) and the Eunice Kennedy Shriver National Institute Of Child Health & Human Development (NICHD) of the National Institutes of Health (NIH) under Grant P41EB027060; and the Center for Reliable Sensor Technology-Based Outcomes for Rehabilitation (RESTORE), which is supported by the Eunice Kennedy Shriver National Institute Of Child Health & Human Development (NICHD) and the National Institute Of Neurological Disorders And Stroke (NINDS) of the National Institutes of Health (NIH) under Grant No. P2CHD101913.
Primary sponsor	Stanford University
Contact for public queries	*MB*, PHD<br>Stanford University<br>boswellm@stanford.edu
Contact for scientific queries	*MB*, PHD<br>Stanford University<br>boswellm@stanford.edu
Title	A Digital Mindset Intervention to Improve Pain and Exercise Participation in Individuals With Knee Osteoarthritis: A Randomized, Parallel-group Study
Countries of recruitment	USA
Health condition(s)	Knee osteoarthritis

or problem(s) studied	
Study type	Interventional<br>Allocation: randomized<br>Intervention model: parallel assignment<br>Masking: Subjects not blind; outcomes assessor blind<br>Primary purpose: Supportive Care
Date of first enrolment	April 2023
Target sample size	501

Choice of comparator

Education and exercise are recommended as first-line treatment for osteoarthritis, but often, “treatment as usual” is pain medication and, eventually, surgery. If patients want information about osteoarthritis, sometimes they are given a pamphlet about osteoarthritis by their doctor. Otherwise, they are left to research osteoarthritis on their own. Our comparator is education videos about osteoarthritis that one would typically find on the internet. While accurate, this information does not typically (or intentionally) target mindset and, as shown in the low physical activity levels and adherence to exercise, does not successfully support behavior change. As this does not put participants at risk, we expect that it will neither improve nor worsen their physical or psychological states.

Supplementary Table 2. Study objectives.

**Primary Objectives**	
Primary outcomes	Primary outcomes assessed at T2 (change from baseline):<br>(i) Exercise mindset using the Mindset about the Process of Health – Exercise scale. This one-factor scale was developed and validated[1] to assess mindset about the process of engaging in physical activity (e.g., physical activity is difficult/easy, unpleasant/pleasurable, boring/fun). The scale consists of 7 items and is measured on a 4-point scale and scored from 1 to 4, with a higher score reflecting a more appeal-focused mindset about exercise<br>(ii) Osteoarthritis mindsets using the Illness Mindset Inventory. The Illness Mindset Inventory measures three mindsets about

	the nature and meaning of illness: that it is a catastrophe, manageable, or an opportunity. The scale consists of 20 items measured on a 6-point scale and scored from 1 to 6, with 10 of those questions capturing mindsets about chronic illness. The extent to which a participant endorsed each mindset was obtained by calculating the respective mean scores. A higher score indicates greater agreement with the mindset. This Illness Mindset Inventory is valid and reliable in individuals with knee osteoarthritis[2]. We adapted the scale to focus on mindsets about "knee osteoarthritis" as opposed to "chronic disease."<br><br>Primary outcomes assessed at T3 (change from baseline):<br>(i) Knee pain using the question, "*What was your average osteoarthritis-related pain over the past week?*" and measured on an 11-point Numeric Rating Scale (NRS) from 0 (no pain at all) to 10 (the worst pain imaginable)<br>(ii) Physical activity using the Physical Activity Scale for the Elderly (PASE). The PASE asks respondents about the frequency of light, moderate, and strenuous work and leisure activities and is a validated measure of self-reported physical activity for individuals with osteoarthritis[3]. The scale is scored from 0 to 793 with higher scores indicating higher levels of physical activity.
**Secondary Objectives**	
Key secondary outcomes	Secondary outcomes assessed at T2 (change from baseline):<br>(i) Knee osteoarthritis knowledge by the Knee Osteoarthritis Knowledge Scale[4]. The Knee Osteoarthritis Knowledge Scale is scored from 11-55 and measures knowledge about osteoarthritis in individuals with knee or hip osteoarthritis<br>(ii) Body mindsets using the Illness Mindset Inventory. In addition to mindsets about chronic illness, the Illness Mindset Inventory measures three mindsets about the nature of the body in the context of a chronic illness: that it is adversarial, responsive, or resilient<br>(iii) Activity adequacy mindsets using the Adequacy of Activity Mindset Measure[5]. This scale was developed to assess mindsets about the adequacy and benefits of one's physical activity as it relates to health. The scale consists of 5 items measured on a 7-point scale and scored from 1 to 7, with a higher score reflecting a more adaptive mindset about the benefits and risks associated with current levels of physical activity.<br><br>Secondary outcomes assessed at T3 (change from baseline):

	(i) Knee pain and functioning using the Short version of the Western Ontario and McMaster Universities Arthritis Index (shortMAC). The shortMAC is a disease-specific 12-item measure of knee symptoms and has shown to be valid and reliable in patients with knee osteoarthritis[6]. We evaluated the measure divided into two subscales: pain and function<br>(ii) Perceived need for surgery using the single question, "*How likely do you think you are of needing knee replacement surgery in the future?*" It was answered on the Likert scale from 1 (very unlikely) to 5 (very likely)<br>(iii) Chosen symptom management strategies using the single question, "*Which of the following are ways in which you manage and/or improve your osteoarthritis symptoms? Please select all that apply.*" It was answered via multiple choice with a multiple-selection option. The options available were the most commonly identified responses as determined by a previous study[7]<br>(iv) Fear of movement using the Brief Fear of Movement Scale for Osteoarthritis[8]. The Brief Fear of Movement Scale for Osteoarthritis is a 6-item scale validated to assess fear of movement in individuals with osteoarthritis<br>(v) Arthritis self-efficacy using the Arthritis-Self Efficacy Scale. This scale is scored from 1 to 10, with higher scores indicating greater self-efficacy, and was divided into the "pain" and "other symptoms" subscales<br>(vi) Physical and Mental Health using the PROMIS v.1.1 Global Health Short Form[9]. This scale is a 10-item survey that measures overall physical function, fatigue, pain, emotional distress, and social health in healthy and clinical adult populations[10]. We evaluated the measure divided into its two subscales: physical health and mental health[11].

Key secondary outcomes	Secondary outcomes assessed at T2 (change from baseline):<br>(i) Knee osteoarthritis knowledge by the Knee Osteoarthritis Knowledge Scale[4]. The Knee Osteoarthritis Knowledge Scale is scored from 11-55 and measures knowledge about osteoarthritis in individuals with knee or hip osteoarthritis<br>(ii) Body mindsets using the Illness Mindset Inventory. In addition to mindsets about chronic illness, the Illness Mindset Inventory measures three mindsets about the nature of the body in the context of a chronic illness: that it is adversarial, responsive, or resilient

	(iii) Activity adequacy mindsets using the Adequacy of Activity Mindset Measure[5]. This scale was developed to assess mindsets about the adequacy and benefits of one's physical activity as it relates to health. The scale consists of 5 items measured on a 7-point scale and scored from 1 to 7, with a higher score reflecting a more adaptive mindset about the benefits and risks associated with current levels of physical activity.<br><br>Secondary outcomes assessed at T3 (change from baseline):<br>(i) Knee pain and functioning using the Short version of the Western Ontario and McMaster Universities Arthritis Index (shortMAC). The shortMAC is a disease-specific 12-item measure of knee symptoms and has shown to be valid and reliable in patients with knee osteoarthritis[6]. We evaluated the measure divided into two subscales: pain and function<br>(ii) Perceived need for surgery using the single question, "*How likely do you think you are of needing knee replacement surgery in the future?*" It was answered on the Likert scale from 1 (very unlikely) to 5 (very likely)<br>(iii) Chosen symptom management strategies using the single question, "*Which of the following are ways in which you manage and/or improve your osteoarthritis symptoms? Please select all that apply.*" It was answered via multiple choice with a multiple-selection option. The options available were the most commonly identified responses as determined by a previous study[7]<br>(iv) Fear of movement using the Brief Fear of Movement Scale for Osteoarthritis[8]. The Brief Fear of Movement Scale for Osteoarthritis is a 6-item scale validated to assess fear of movement in individuals with osteoarthritis<br>(v) Arthritis self-efficacy using the Arthritis-Self Efficacy Scale. This scale is scored from 1 to 10, with higher scores indicating greater self-efficacy, and was divided into the "pain" and "other symptoms" subscales<br>(vi) Physical and Mental Health using the PROMIS v.1.1 Global Health Short Form[9]. This scale is a 10-item survey that measures overall physical function, fatigue, pain, emotional distress, and social health in healthy and clinical adult populations[10]. We evaluated the measure divided into its two subscales: physical health and mental health[11].

Research Hypotheses

Hypothesis 1. A digital education and mindset intervention will significantly improve mindsets about the process of exercising and osteoarthritis among adults aged ≥ 45

years with knee osteoarthritis compared to an active comparison education intervention and no-intervention immediately post-intervention.

Hypothesis 2. A digital education and mindset intervention will significantly reduce knee pain and increase physical activity levels among adults aged ≥ 45 years with knee osteoarthritis compared to an active comparison education intervention and a no-intervention control group one-month post-intervention.

Hypothesis 3. A digital education and mindset intervention will significantly improve knee osteoarthritis knowledge and mindsets about activity adequacy and the body among adults aged ≥ 45 years with knee osteoarthritis compared to an active comparison education intervention and a no intervention control group immediately post-intervention.

Hypothesis 4. A digital education and mindset intervention will significantly improve knee pain and function, perceived need for surgery, symptom management, arthritis self-efficacy, fear of movement, physical health, mental health, among adults aged ≥ 45 years with knee osteoarthritis compared to an active-comparator education group and a no-intervention control group one-month post-intervention.

Key Personnel

Trial Coordinator
Dr. Melissa Boswell, boswellm@stanford.edu
Department of Bioengineering
Stanford University, Stanford, California, USA

Statisticians
Dr. Trevor Hastie
Department of Statistics
Stanford University, Stanford, California, USA

Disha Ghandwani
Department of Statistics
Stanford University, Stanford, California, USA

Trial Design

We will randomize 505 individuals aged ≥ 45 years with knee osteoarthritis into one of three groups with 1:1:1 group allocation: the Mindset Group (participants receive a digital mindset intervention to improve mindsets about osteoarthritis and exercise), the Education Group (Participants receive a series of osteoarthritis education videos and reflective questions that matches the digital mindset intervention in duration and attention), or the No-Intervention Group (participants take the same surveys as the other groups at the same time points, but do not receive any additional content). Participants

will complete an initial baseline survey and be randomized one week later. Those randomized to the Mindset and Eduction Groups will have one week to complete the self-paced online programs. All groups complete osteoarthritis knowledge and mindset surveys at this timepoint. One-month later, participants will receive a follow-up questionnaire.

Study Setting

The study will be conducted entirely online.

Eligibility Criteria

Inclusion Criteria:

- Over 45 years of age
- Self-reported doctor's diagnosis of knee osteoarthritis OR meets the National Institute for - - Health and Care Excellence osteoarthritis clinical criteria (activity-related knee pain and no knee morning stiffness lasting ≥ 30 minutes)
- Knee pain for at least 3 months
- Ability to walk unaided
- Can read and write in English
- Consistent internet access
- Willingness and ability to comply with the study requirements

Exclusion criteria:

- Past total knee arthroplasty or scheduled surgical procedure on any back or lower limb with osteoarthritis within the next 12 months
- Recent serious injury (within the past 2 months) on the knee(s) with osteoarthritis
- Any condition making it unsafe to participate in physical activity
- Intra-articular therapy within the past 6 months (e.g. injections such as corticosteroids and hyaluronic acid)
- Participates in physical exercise for 30 minutes or more 5 days per week

Interventions

Intervention(s)	Mindset Group:<br>Participants receive a digital mindset intervention to improve mindsets about osteoarthritis and exercise.<br><br>Education Group (active comparison):

	Participants receive a series of osteoarthritis education videos and reflective questions that matches the digital mindset intervention in duration and attention.<br><br>No-Intervention (control):<br>Participants take the same surveys as the other groups at the same time points, but do not receive any additional content.

Modifications

Discontinuation of the program due to psychological disturbance. We do not expect participants to have a psychological disturbance due to the mindset intervention or active comparator program. In the case that someone refuses to continue the study due to a psychological disturbance from participation, this should be reported as an adverse event.

Adherence

Adherence reminders will be in the form of emails. Three reminders will be sent out: once before the deadline, on the deadline, and the day after the deadline. Reminders will include the importance of completing the section in order to remain in the study. Reminders will also include a flow chart of the study timeline and components, with the relevant section highlighted. Participants will be able reply to the study coordinator with questions.

Concomitant Care

Participation is at the discretion of the participants. Thus, the other option for participants is to not participate in the study.

Prohibited Concomitant Treatment. Additional physical therapy treatment is not permitted during the study as it would interfere with the main outcomes of our study.

Study Flow and Timeline

Supplementary Figure 1. Study flow and timeline.

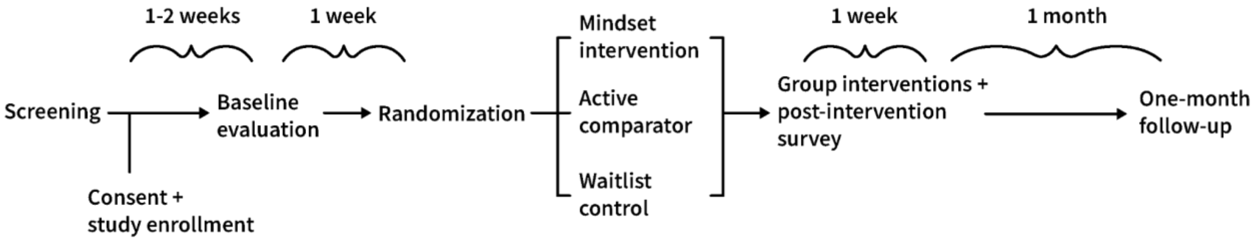

This figure illustrates the study's flow and timeline, from initial screening to the one-month follow-up. It shows key stages, including baseline evaluation, randomization, intervention periods, and assessment points.

Sample Size

The efficacy of the mindset intervention will be deemed successful if enhancements in either or both key outcomes, namely pain intensity (NRS) or physical activity levels (PASE), are observed. Anticipating modest effect sizes between the mindset and educational cohorts, we conducted a preliminary power analysis, similar to prior online education studies for knee osteoarthritis[12,13], using G*Power 3.1 with a presumed effect size of 0.3 (see below). This analysis involved comparing the mindset group against both the education and no-intervention groups, leading to four distinct comparisons. These assessments are planned to be executed at a 5% significance level, aiming for an 80% probability of discerning a noticeable improvement in delta. Based on Boswell et al.[7], we estimated the standard deviations for PASE and pain at 70.28 and 1.67, respectively. A 26-point difference in PASE was selected as the target delta. While no clinically significant benchmarks for PASE has been established, the intervention's focus on promoting walking and moderate exercise led us to define this delta as the equivalent shift in walking and light exercise frequency from “seldom” (<1 hour per day) to “sometimes” (1-2 hours per day), as per the PASE scale, giving a goal of 26 points. This determination necessitates a sample size of 139 participants per group, totaling 417. To compensate for an anticipated 20% dropout rate, we plan to enroll 501 participants. This sample size is adequately powered to detect a clinically relevant difference of 2 points in NRS pain[14].

A priori power analysis

Disha Ghandwani

11/29/2022

Let, y_i^b represents outcome at baseline and y_i^f represents outcome at follow-up for i^{th} person in treatment group, and $\bar{y}^b$ and $\bar{y}^f$ represents respective means. Similarly, x_i^b represents outcome at baseline and x_i^f represents outcome at follow-up for i^{th} person in control group, and $\bar{x}^b$ and $\bar{x}^f$ represents respective means.

Assumption: Let $x_i^f - x_i^b \sim \mathcal{N}(\Delta_1, \sigma^2)$ and $y_i^f - y_i^b \sim \mathcal{N}(\Delta_2, \sigma^2)$.

Let's say $\Delta = \Delta_2 - \Delta_1$, we want to test the hypothesis

$$H_0 : \Delta = 0$$

$$\text{v/s } H_1 : \Delta > 0$$

Under H_0, $T = (\bar{y}^f - \bar{y}^b) - (\bar{x}^f - \bar{x}^b) \sim \mathcal{N}(0, 2\sigma^2/n)$. We reject H_0 if $\frac{T}{\sqrt{2\sigma^2/n}} > z_{1-\alpha}$. We want n so that power at Δ is $1 - \beta$, i.e.,

$$P_\Delta\left(\frac{T}{\sqrt{2\sigma^2/n}} > z_{1-\alpha}\right) \geq 1 - \beta$$

which is equivalent to

$$n \geq \frac{2\sigma^2(z_{1-\alpha} + z_{1-\beta})^2}{\Delta^2}$$

For our analysis, we choose $\alpha = 0.05$, and try four different values of β,0.05,0.10, 0.15, and 0.20. We estimate σ from pilot data.

Supplementary Figure 2. Baseline vs. Follow-up PASE Scores with Difference Distribution.

Outcome variable, PASE

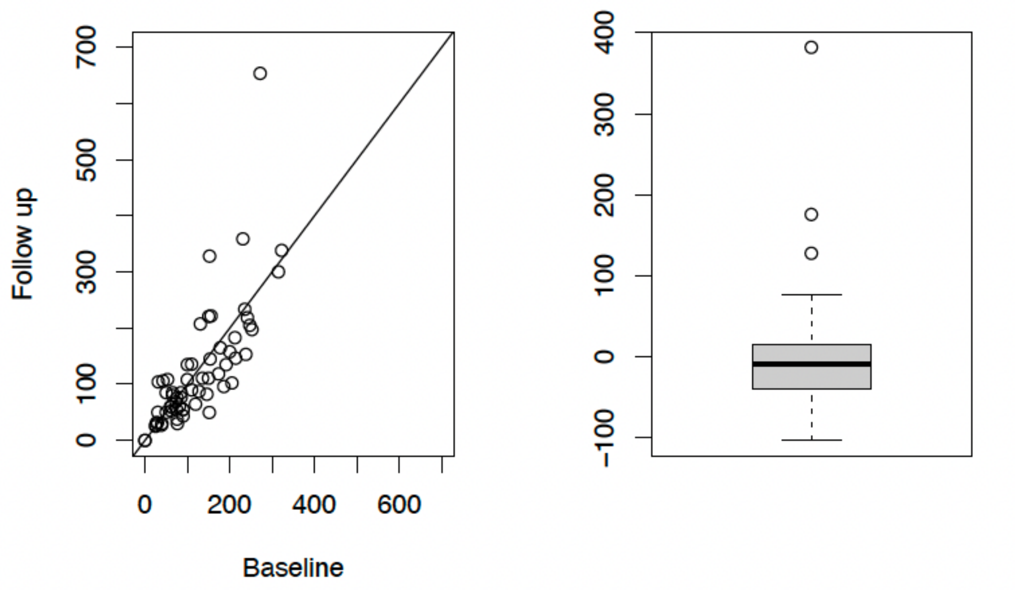


```
## sigma =  70.27939
```

This figure presents the relationship between baseline and follow-up scores for the Physical Activity Scale for the Elderly (PASE). The left panel shows a scatter plot of baseline scores (x-axis) versus follow-up scores (y-axis), with a reference line indicating no change. Each point represents an individual participant. The right panel displays a box plot of the differences between follow-up and baseline scores, providing a visual summary of the distribution of changes in PASE scores over the study period. The box plot shows the median, interquartile range, and potential outliers in score differences.

Supplementary Figure 3. A Priori Sample Size Analysis for Outcome Variable PASE.

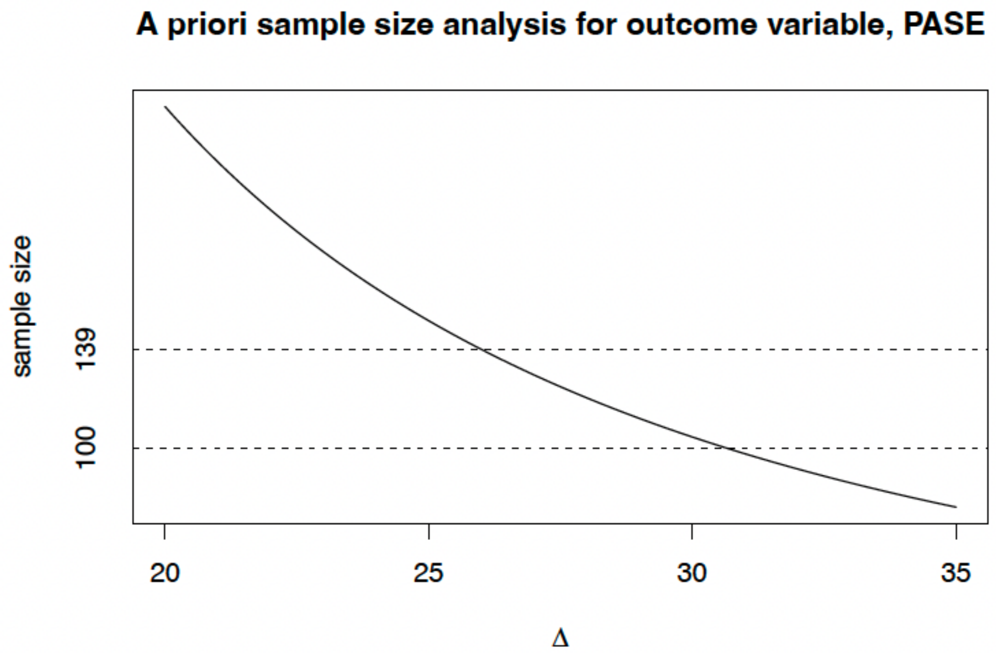

This figure illustrates the relationship between the effect size (Δ) and the required sample size for the Physical Activity Scale for the Elderly (PASE) outcome variable. The x-axis represents the effect size (Δ), ranging from 20 to 35, while the y-axis shows the corresponding sample size.

Supplementary Figure 4. Baseline vs. Follow-up Pain Scores with Change Distribution

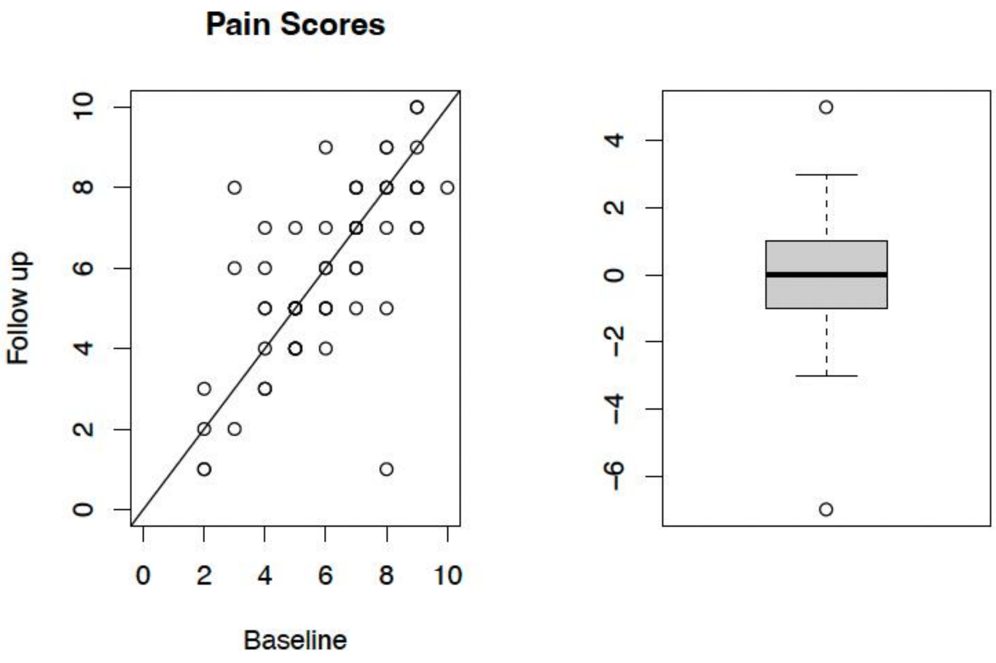


```
## sigma =  1.670426
```

This figure presents the analysis of pain scores at baseline and follow-up. The left panel shows a scatter plot comparing baseline pain scores (x-axis) to follow-up pain scores (y-axis). Each point represents an individual participant, with a reference line indicating no change. The right panel displays a box plot illustrating the distribution of changes in pain scores from baseline to follow-up. The box plot shows the median, interquartile range, and potential outliers in score differences.

Supplementary Figure 5. A Priori Sample Size Analysis for Outcome Variable Pain.

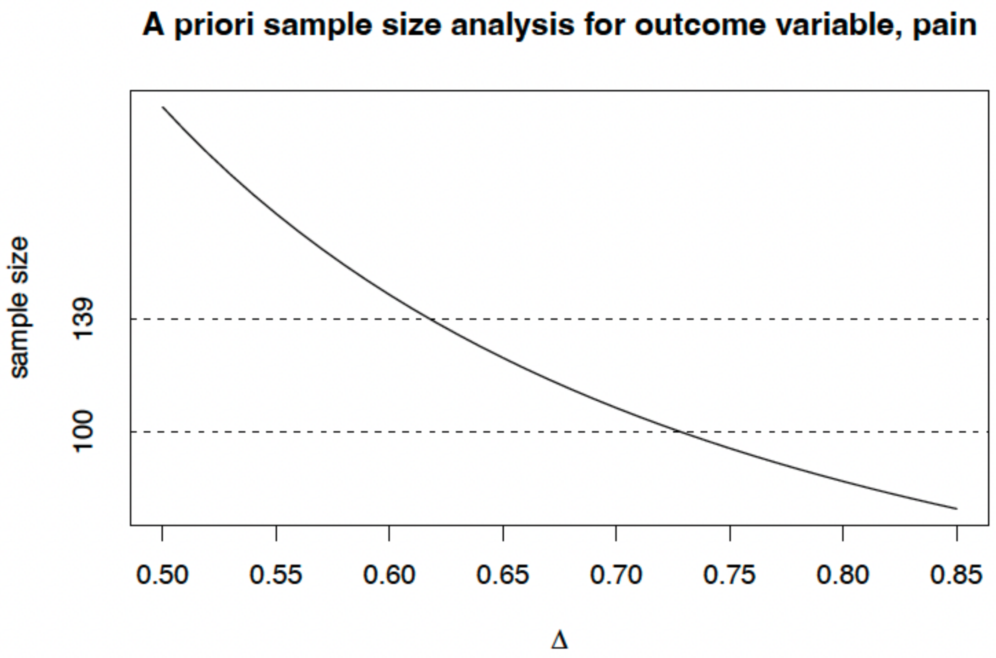

This figure illustrates the relationship between effect size (Δ) and required sample size for the pain outcome variable. The x-axis represents the effect size (Δ), ranging from 0.50 to 0.85, while the y-axis shows the corresponding sample size.

Recruitment

Participants will be recruited through paid online and social media advertising and local flyer postings.

Allocation

We will use the randomizer function in Qualitrics to randomize participants into one of three groups (1:1:1): the Mindset Group, the Education Group (active comparison), or the No–Intervention Group (control).

Blinding (Masking)

During the informed consent process, participants will be informed that the purpose of the study is to understand the effect of a new online osteoarthritis program and that they will be randomized to either a no-intervention group or one of two different sets of videos and reflective questions. Participants will be aware of whether they are randomized to an online program; however, the differences in content between the two programs and the hypotheses of the study will not be revealed. The biostatistician performing the data analysis will be blinded to the group labels.

Data Collection, Management, and Access

All data will be collected through the Qualitrics online survey software. Qualitrics encrypts all transmitted data using Transport Layer Security and hosts data on data centers that are independently audited using the industry standard SSAE-18 method. Stanford University has a Qualtrics license for research use, and data obtained from the Qualitric platform can only be accessed through a secure login. Data will be exported to Microsoft Excel and securely stored for use by the research team. Only after de-identification may the data be shared outside of the research team.

Missing Data

Participants who did not complete a survey entirely were removed from the study and not sent the follow-up survey. Each question of each survey was “force response,” meaning that if a participant completed the survey, no answers would be missing from the response. Thus, no methods for missing data were implemented.

Statistical Methods

Statistical analysis will be carried out in R[15] by a blinded statistician, Disha Ghandwani, with no knowledge of group allocation.

Standard descriptive statistics will be presented for all participant characteristic measures at baseline. Prior to analysis, t-tests for continuous variables and chi-square ($\chi 2$) tests for categorical variables will be conducted to test the equality of means of baseline characteristics. Additionally, baseline characteristic comparisons between participants who completed the study and those who dropped out will be reported.

Changes in primary and secondary mindsets post-intervention will be compared among the mindset intervention group versus the active attention control (education group) and the no-intervention control group. We will present standard descriptive statistics of and calculate changes from baseline to post-intervention for the mindsets and knee osteoarthritis knowledge and from baseline to one-month follow-up for all other outcome variables. Changes will be presented as the mean change with 95% confidence intervals, along with Cohen d, using the following formula:

$$cohen\ d\ = \frac{\bar{x_2} - \bar{x_1}}{s}$$

where $\bar{x_2}$ denotes the mean of variable after intervention, $\bar{x_1}$ denotes the mean of variable before intervention, and s denotes the standard deviation of the change in the variable due to intervention.

We will perform the t-test to compare the change in outcome variables post-intervention and at follow-up between the mindset group and the education and no-intervention groups. We assume the equality of variance for the changes. The t-statistic is given by:

$$\frac{\bar{X} - \bar{Y}}{s_{pooled}\sqrt{\frac{1}{m} + \frac{1}{n}}}$$

where $\bar{X}$ is the mean of changes in the first group and $\bar{Y}$ is the mean of the changes in the second group, s_{pooled} is the pooled standard deviation from the two groups. We assume that there were m people in the first group and n people in the second group. After calculating the t-statistics, the p-values will be computed to estimate the statistical significance of differences in changes between groups. We will perform Bonferonni correction for the four comparisons (e.g., the mindset group vs. the education group and the mindset group vs. the no-intervention group for pain and physical activity at one-month follow-up), which adjusts the significance threshold to $P = 0.0125$. The comparison of the education group to the no-intervention group will not be a primary comparison.

Data Monitoring

The trial coordinator will provide ongoing project oversight and will meet regularly with the senior investigators throughout the project period to provide study feedback including recruitment, retention, and adverse events. During the study, participants have access to email the study coordinator at any time to report adverse events.

Harms

The potential harms associated with this study include psychological complications resulting from the assessments or mindset and education programs or medical risks due to increasing physical activity levels. There is a small risk that individuals completing psychological questionnaires may become distressed, but there is no evidence of resulting psychological dysfunction. We performed pilot testing of the program and questionnaires in which there was no evidence of psychological complications or the risk of such complications. Further, while the program presents the benefits of physical activity, no specific programs are requested for participation and participants are encouraged to go slow and listen to their bodies.

Therefore, no harm or adverse events are foreseen in this study. However, if a complaint or adverse event arises, the research team will discuss and take appropriate action to manage the issue. Any serious adverse event or harm reported by participants will be recorded and reported.

Research Ethics Approval

The study protocol, consent form, and recruitment material will be approved by the Stanford University Research Compliance Office Institutional Review Board.

Protocol Amendments

Protocol amendments will be documented and reported.

Consent Or Assent

Participants will be directed from recruitment materials to a RedCap survey. The survey will first be provided with information on the purpose of the study and the requirements for participation. After that, participants will be given a survey to determine whether they can participate. If participants pass the qualifying survey, they will be directed to the online consent form, where they have the option to consent to participate in the study digitally.

Confidentiality

Personal or identifiable information obtained during this study is considered confidential. Participant confidentiality will be ensured by using identification code numbers corresponding to data across time points. Identifiable data will not be included in the dissemination of the manuscript and its accompanying data. See Data Management for additional information on the security of personal data.

Declaration Of Interests

None of the investigator team has any financial or other competing interests to declare.

Ancillary And Post-Trial Care

Participants who agree to be contacted for future research during the consent process may be contacted for ancillary studies. Any ancillary studies will be submitted to the Institutional Review Board for approval.

After study completion, all participants will be offered the mindset intervention program.

Dissemination Policy

The study will be submitted to peer-reviewed journals for publication. The results will be presented at national and international conferences. The results may also be shared in news outlets, such as online articles, newspapers, and magazines. Participant identity will not be disclosed when publishing or presenting the results. Participants will be informed of the results if requested.

Supplementary Table 3. CONSORT Checklist

Reporting checklist for a randomized trial.

		Reporting Item	Page Number
**Title and Abstract**			
Title	#1a	Identification as a randomized trial in the title.	1
Abstract	#1b	Structured summary of trial design, methods, results, and conclusions	3
**Introduction**			
Background and objectives	#2a	Scientific background and explanation of rationale	4
Background and objectives	#2b	Specific objectives or hypothesis	4
**Methods**			
Trial design	#3a	Description of trial design (such as parallel, factorial) including allocation ratio.	4
Trial design	#3b	Important changes to methods after trial commencement (such as eligibility criteria), with reasons	N/A

Participants	#4a	Eligibility criteria for participants	5
Participants	#4b	Settings and locations where the data were collected	5
Interventions	#5	The experimental and control interventions for each group with sufficient details to allow replication, including how and when they were actually administered	5-6
Outcomes	#6a	Completely defined prespecified primary and secondary outcome measures, including how and when they were assessed	6-7
Outcomes	#6b	Any changes to trial outcomes after the trial commenced, with reasons	N/A
Sample size	#7a	How sample size was determined.	7
Sample size	#7b	When applicable, explanation of any interim analyses and stopping guidelines	N/A
Randomization - Sequence generation	#8a	Method used to generate the random allocation sequence.	5

Randomization - Sequence generation	#8b	Type of randomization; details of any restriction (such as blocking and block size)	5
Randomization - Allocation concealment mechanism	#9	Mechanism used to implement the random allocation sequence (such as sequentially numbered containers), describing any steps taken to conceal the sequence until interventions were assigned	5
Randomization - Implementation	#10	Who generated the allocation sequence, who enrolled participants, and who assigned participants to interventions	5
Blinding	#11a	If done, who was blinded after assignment to interventions (for example, participants, care providers, those assessing outcomes) and how.	5
Blinding	#11b	If relevant, description of the similarity of interventions	5
Statistical methods	#12a	Statistical methods used to compare groups for primary and secondary outcomes	7
Statistical methods	#12b	Methods for additional analyses, such as subgroup analyses and adjusted analyses	7

Results

Participant flow diagram (strongly recommended)	#13a	For each group, the numbers of participants who were randomly assigned, received intended treatment, and were analysed for the primary outcome	7
Participant flow	#13b	For each group, losses and exclusions after randomization, together with reason	7
Recruitment	#14a	Dates defining the periods of recruitment and follow-up	7
Recruitment	#14b	Why the trial ended or was stopped	N/A
Baseline data	#15	A table showing baseline demographic and clinical characteristics for each group	7
Numbers analysed	#16	For each group, number of participants (denominator) included in each analysis and whether the analysis was by original assigned groups	7
Outcomes and estimation	#17a	For each primary and secondary outcome, results for each group, and the estimated effect size and its precision (such as 95% confidence interval)	8

Outcomes and estimation	#17b	For binary outcomes, presentation of both absolute and relative effect sizes is recommended	8
Ancillary analyses	#18	Results of any other analyses performed, including subgroup analyses and adjusted analyses, distinguishing pre-specified from exploratory	9
Harms	#19	All important harms or unintended effects in each group (For specific guidance see CONSORT for harms)	7
**Discussion**			
Limitations	#20	Trial limitations, addressing sources of potential bias, imprecision, and, if relevant, multiplicity of analyses	11
Generalisability	#21	Generalisability (external validity, applicability) of the trial findings	11
Interpretation	#22	Interpretation consistent with results, balancing benefits and harms, and considering other relevant evidence	10
Registration	#23	Registration number and name of trial registry	2,4
**Other information**			

Interpretation	#22	Interpretation consistent with results, balancing benefits and harms, and considering other relevant evidence	11
Registration	#23	Registration number and name of trial registry	2,4
Protocol	#24	Where the full trial protocol can be accessed, if available	4
Funding	#25	Sources of funding and other support (such as supply of drugs), role of funders	12

None The CONSORT checklist is distributed under the terms of the Creative Commons Attribution License CC-BY. This checklist can be completed online using https://www.goodreports.org/, a tool made by the EQUATOR Network in collaboration with Penelope.ai

Supplementary Note 1. Informed Consent Materials

FOR QUESTIONS ABOUT THE STUDY, CONTACT:
Melissa Boswell
Email: boswellm@stanford.edu

Are you participating in any other research studies? _____ Yes _____No

DESCRIPTION: You are invited to participate in a research study on the use of digital osteoarthritis management programs. The purpose of the study is to understand the effect of a new online osteoarthritis program.

PROCEDURES: If you choose to participate, you will be randomized to either a waitlist group or one of two different sets of videos and reflective questions. You have a one in three (33.3%) chance of being assigned to each group. Each group is equally important for the study. Regardless of which group you are assigned to, you will be asked to fill out surveys, watch videos, answer reflection questions, and provide feedback on the program content. You will also be given the option to perform a functional assessment consisting of moving from a seated position to standing 5 times, which you can record and upload within the online survey.

DURATION OF STUDY INVOLVEMENT: Each participant will be actively enrolled in the study for the one-month duration of the study.

Enrollment will occur throughout the United States. Stanford University expects to enroll 501 research study participants in this research study.

Identifiers might be removed from identifiable private information, and videos will be de-identified with face-blurring. After such removal, the information could be used for future research studies or distributed to another investigator for future research studies without additional informed consent from you.

RISKS AND BENEFITS: The risks associated with this study are minimal, but there is a small risk of injury consistent with increasing daily physical activity and performing the sit-to-stand movement. Such injuries are temporary and minor (i.e., muscle fatigue and soreness). We will do everything possible to maintain your confidentiality during the study, but

there is the potential risk of breach of confidentiality in which your video and survey results may be linked to your name. Study data, including videos, will be stored securely, in compliance with Stanford University standards, minimizing the risk of a confidentiality breach. Again, we will do our best to keep your data confidential.

The benefits which may reasonably be expected to result from this study are that you will contribute to improving the program for other people with osteoarthritis in the future. You may also benefit from an increased understanding of knee osteoarthritis. We cannot and do not guarantee or promise that you will receive any benefits from this study.

TIME INVOLVEMENT: Your participation will take approximately 4 hours over the course of five weeks. It will take around 30 minutes for the initial survey. You then may be asked to complete a program that will take around 3 hours over the course of one week. There will also be a follow-up survey one month after the start of the study at which you will be asked to take another 30-minute survey.

PAYMENTS: You will receive up to a total of $50 in gift cards as payment for your participation, split into three payments:

- $10 after the first survey
- $10 after the second survey
- $30 after the third and final survey

PARTICIPANT'S RIGHTS: If you have read this form and have decided to participate in this project, please understand your participation is voluntary and you have the right to withdraw your consent or discontinue participation at any time without penalty or loss of benefits to which you are otherwise entitled.

Your decision not to participate will not have any negative effect on you or your medical care. You have the right to refuse to answer particular questions. The alternative to participation in this study is to not participate.

The results of this research study may be presented at scientific or professional meetings or published in scientific journals. However, your identity will not be disclosed.

A description of this clinical trial will be available on http://www.ClinicalTrials.gov, as required by U.S. Law. This Web site will not include information that can identify you. At most, the Web site will include a summary of the results. You can search this website at any time.

Authorization To Use Your Health Information For Research Purposes
Because information about you and your health is personal and private, it generally cannot be used in this research study without your written authorization. If you sign this form, it will provide that authorization. The form is intended to inform you about how your health information will be used or disclosed in the study. Your information will only be used in accordance with this authorization form and the informed consent form and as required or allowed by law. Please read it carefully before signing it.

What is the purpose of this research study and how will my health information be utilized in the study?
The purpose of this study is to evaluate digital osteoarthritis management programs for individuals with knee osteoarthritis. Health information will be evaluated over the course of the study and the results may be published in a conference and/or research journal. As this is a clinical trial, the information provided will, in some form, be submitted to the sponsor and other federal agencies as required.

Do I have to sign this authorization form?
You do not have to sign this authorization form. But if you do not, you will not be able to participate in this research study. Signing the form is not a condition for receiving any medical care outside the study.

If I sign, can I revoke it or withdraw from the research later?
If you decide to participate, you are free to withdraw your authorization regarding the use and disclosure of your health information (and to discontinue any other participation in the study) at any time. After any revocation, your health information will no longer be used or disclosed in the study, except to the extent that the law allows us to continue using your information (e.g., necessary to maintain integrity of research). If you wish to revoke your authorization for the research use or disclosure of your health information in this study, you must write to: Melissa Boswell (boswellm@stanford.edu).

What Personal Information Will Be Obtained, Used or Disclosed?
Your health information related to this study, may be used or disclosed in connection with this research study, including, but not limited to: name; email; self-reported health and demographic information including age, height, weight, sex, gender, ethnicity, state of residence, education, employment status, marital status, medical issues, osteoarthritis status, physical activity level, mental health, and physical health; physical activity; video of the sit-to-stand test.

Who May Use or Disclose the Information?
The following parties are authorized to use and/or disclose your health information in connection with this research study:

- The Protocol Director, Melissa Boswell
- The Stanford University Administrative Panel on Human Subjects in Medical Research and any other unit of Stanford University as necessary
- Research Staff

Who May Receive or Use the Information?
The parties listed in the preceding paragraph may disclose your health information to the following persons and organizations for their use in connection with this research study:

- The Office for Human Research Protections in the U.S. Department of Health and Human Services

Your information may be re-disclosed by the recipients described above, if they are not required by law to protect the privacy of the information.

When will my authorization expire?
Your authorization for the use and/or disclosure of your health information will end on December 31, 2050 or when the research project ends, whichever is earlier.

Will access to my medical record be limited during the study?
To maintain the integrity of this research study, you may not have access to any health information developed as part of this study until it is

completed. At that point, you would have access to such health information if it was used to make a medical or billing decision about you (e.g., if included in your official medical record).

You give consent for your video recordings to be used for (describe proposed use of the recordings and what will happen to the recordings, e.g., shown at scientific meetings; and describe the final disposition of the tapes).(Please note, this option is also applicable if the recordings are used for purposes that are not part of this research project, e.g. future analysis, professional presentations, etc)

Please initial your choice: ___Yes ___No

_____________________________ _____________

Signature of Adult Participant Date

____

Print Name of Adult Participant

WITHDRAWAL FROM STUDY: The Protocol Director may also withdraw you from the study and the study medication may be stopped without your consent for one or more of the following reasons:

- o Failure to follow the instructions of the Protocol Director and study staff.
- o The Protocol Director decides that continuing your participation could be harmful to you.
- o The study is cancelled.
- o Other administrative reasons.
- o Unanticipated circumstances.

SPONSOR:

Stanford University is providing financial support for this study.

CONTACT INFORMATION:

Questions, Concerns, or Complaints: If you have any questions, concerns or complaints about this research study, its procedures, risks and benefits, or alternative courses of treatment, you should ask the Protocol Director, Melissa Boswell. You may contact her now or later at [phone number].

Injury Notification: If you feel you have been hurt by being a part of this study, please contact the Protocol Director, Melissa Boswell, at [phone number].

Independent Contact: If you are not satisfied with how this study is being conducted, or if you have any concerns, complaints, or general questions about the research or your rights as a participant, please contact the Stanford Institutional Review Board (IRB) to speak to someone independent of the research team at (650)-723-5244 or toll free at 1-866-680-2906. You can also write to the Stanford IRB, Stanford University, 1705 El Camino Real, Palo Alto, CA 94306.

EXPERIMENTAL SUBJECTS BILL OF RIGHTS: As a research participant you have the following rights. These rights include but are not limited to the participant's right to:

- be informed of the nature and purpose of the experiment;
- be given an explanation of the procedures to be followed in the medical experiment, and any drug or device to be utilized;
- be given a description of any attendant discomforts and risks reasonably to be expected;
- be given an explanation of any benefits to the subject reasonably to be expected, if applicable;
- be given a disclosure of any appropriate alternatives, drugs or devices that might be advantageous to the subject, their relative risks and benefits;
- be informed of the avenues of medical treatment, if any available to the subject after the experiment if complications should arise;
- be given an opportunity to ask questions concerning the experiment or the procedures involved;
- be instructed that consent to participate in the medical experiment may be withdrawn at any time and the subject may discontinue participation without prejudice;
- be given a copy of the signed and dated consent form; and

- be given the opportunity to decide to consent or not to consent to a medical experiment without the intervention of any element of force, fraud, deceit, duress, coercion or undue influence on the subject's decision.

May we contact you about future studies that may be of interest to you?
____ Yes ____ No

______________________________ ___________
Signature of Adult Participant Date

____
Print Name of Adult Participant

Please download the copy of your signed consent form below.

Supplementary References

1. Boles DZ, DeSousa M, Turnwald BP, et al. Can Exercising and Eating Healthy Be Fun and Indulgent Instead of Boring and Depriving? Targeting Mindsets About the Process of Engaging in Healthy Behaviors. *Frontiers in Psychology*. 2021;12. doi:10.3389/fpsyg.2021.745950

2. Zion S. *From Cancer to COVID-19: The Self-Fulfilling Effects of Illness Mindsets on Physical, Social, and Emotional Functioning*. Stanford University; 2021.

3. Smith RD, Healey EL, McHugh GA. Measurement Properties of Self-Reported Physical Activity Instruments Suitable for Osteoarthritis (OA) or Joint Pain Populations: A Systematic Review. *Annals of the*. Published online 2013. https://ard.bmj.com/content/72/Suppl_3/A700.1.abstract?casa_token=sxLNwI0e9OcAAAAA:uwTvM8u2yNPNSvw4JMJeR1jLD-GPEFlZQMjjZd150JYWEpWxbX5St0KbAWlXThiFIDst6znM

4. Darlow B, Abbott H, Bennell K, et al. Knowledge about osteoarthritis: Development of the Hip and Knee Osteoarthritis Knowledge Scales and protocol for testing their measurement properties. *Osteoarthritis and Cartilage Open*. 2021;3(2):100160.

5. Zahrt OH, Evans K, Murnane E, et al. Effects of Wearable Fitness Trackers and Activity Adequacy Mindsets on Affect, Behavior, and Health: Longitudinal Randomized Controlled Trial. *J Med Internet Res*. 2023;25:e40529.

6. Abbott JH, Hobbs C, Gwynne-Jones D, MOA Trial Team. The ShortMAC: Minimum Important Change of a Reduced Version of the Western Ontario and McMaster Universities Osteoarthritis Index. *J Orthop Sports Phys Ther*. 2018;48(2):81-86.

7. Boswell MA, Evans KM, Zion SR, et al. Mindset is associated with future physical activity and management strategies in individuals with knee osteoarthritis. *Ann Phys Rehabil Med*. 2022;65(6):101634.

8. Shelby RA, Somers TJ, Keefe FJ, et al. Brief Fear of Movement Scale for osteoarthritis. *Arthritis Care Res* . 2012;64(6):862-871.

9. Cella D, Riley W, Stone A, et al. The Patient-Reported Outcomes Measurement Information System (PROMIS) developed and tested its first wave of adult self-reported health outcome item banks: 2005-2008. *J Clin Epidemiol*. 2010;63(11):1179-1194.

10. Gregory JJ, Werth PM, Reilly CA, Jevsevar DS. Cross-specialty PROMIS-global health differential item functioning. *Qual Life Res*. 2021;30(8):2339-2348.

11. Parker DJ, Werth PM, Christensen DD, Jevsevar DS. Differential item functioning to validate setting of delivery compatibility in PROMIS-global health. *Qual Life Res*. 2022;31(7):2189-2200.

12. Bennell KL, Lawford BJ, Keating C, et al. Comparing Video-Based, Telehealth-Delivered Exercise and Weight Loss Programs With Online Education on Outcomes of Knee Osteoarthritis : A Randomized Trial. *Ann Intern Med*. 2022;175(2):198-209.

13. Egerton T, Bennell KL, McManus F, Lamb KE, Hinman RS. Comparative effect of two educational videos on self-efficacy and kinesiophobia in people with knee osteoarthritis: an online randomised controlled trial. *Osteoarthritis Cartilage*. Published online June 22, 2022. doi:10.1016/j.joca.2022.05.010

14. Salaffi F, Stancati A, Silvestri CA, Ciapetti A, Grassi W. Minimal clinically important changes in chronic musculoskeletal pain intensity measured on a numerical rating scale. *Eur J Pain*. 2004;8(4):283-291.

15. Core Team R. R: A language and environment for statistical computing. Version 3.6. 0. Vienna, Austria. */ra-language-and-environment-for-statistical-computing*.

16. Bunzli S, O'Brien P, Ayton D, et al. Misconceptions and the Acceptance of Evidence-based Nonsurgical Interventions for Knee Osteoarthritis. A Qualitative Study. *Clin Orthop Relat Res*. 2019;477(9):1975-1983.

17. Hendry M, Williams NH, Markland D, Wilkinson C, Maddison P. Why should we exercise when our knees hurt? A qualitative study of primary care patients with osteoarthritis of the knee. *Fam Pract*. 2006;23(5):558-567.

18. MacKay C, Jaglal SB, Sale J, Badley EM, Davis AM. A qualitative study of the consequences of knee symptoms: “It”s like you're an athlete and you go to a couch potato'. *BMJ Open*. 2014;4(10):e006006.

19. Darlow B, Brown M, Thompson B, et al. Living with osteoarthritis is a balancing act: an exploration of patients' beliefs about knee pain. *BMC Rheumatol*. 2018;2:15.

20. Crum AJ, Langer EJ. Mind-set matters: exercise and the placebo effect. *Psychol Sci*. 2007;18(2):165-171.

21. Crum AJ, Santoro E, Handley-Miner I, et al. Evaluation of the “rethink stress” mindset intervention: A metacognitive approach to changing mindsets. *J Exp Psychol Gen*. 2023;152(9):2603-2622.

Recruitment Content

Supplementary Figure 6. Social media and online advertisement image.

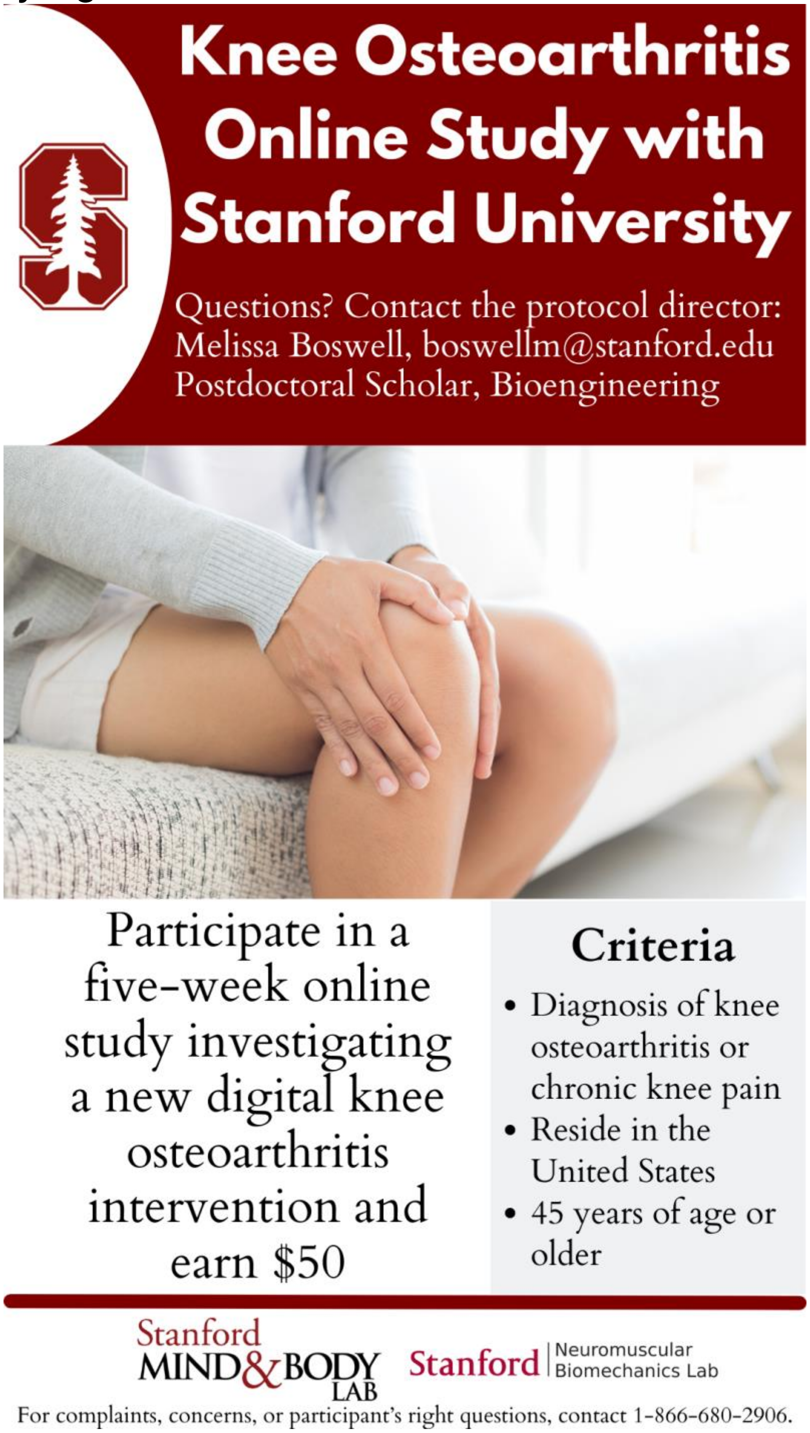

This recruitment image for a Stanford University online study on knee osteoarthritis outlines key details including the five-week duration, $50 compensation, and eligibility criteria, alongside contact information and relevant institutional logos. The central image of hands on a knee visually reinforces the study's focus on knee health.

Supplementary Figure 7. Printed newspaper advertisement image.

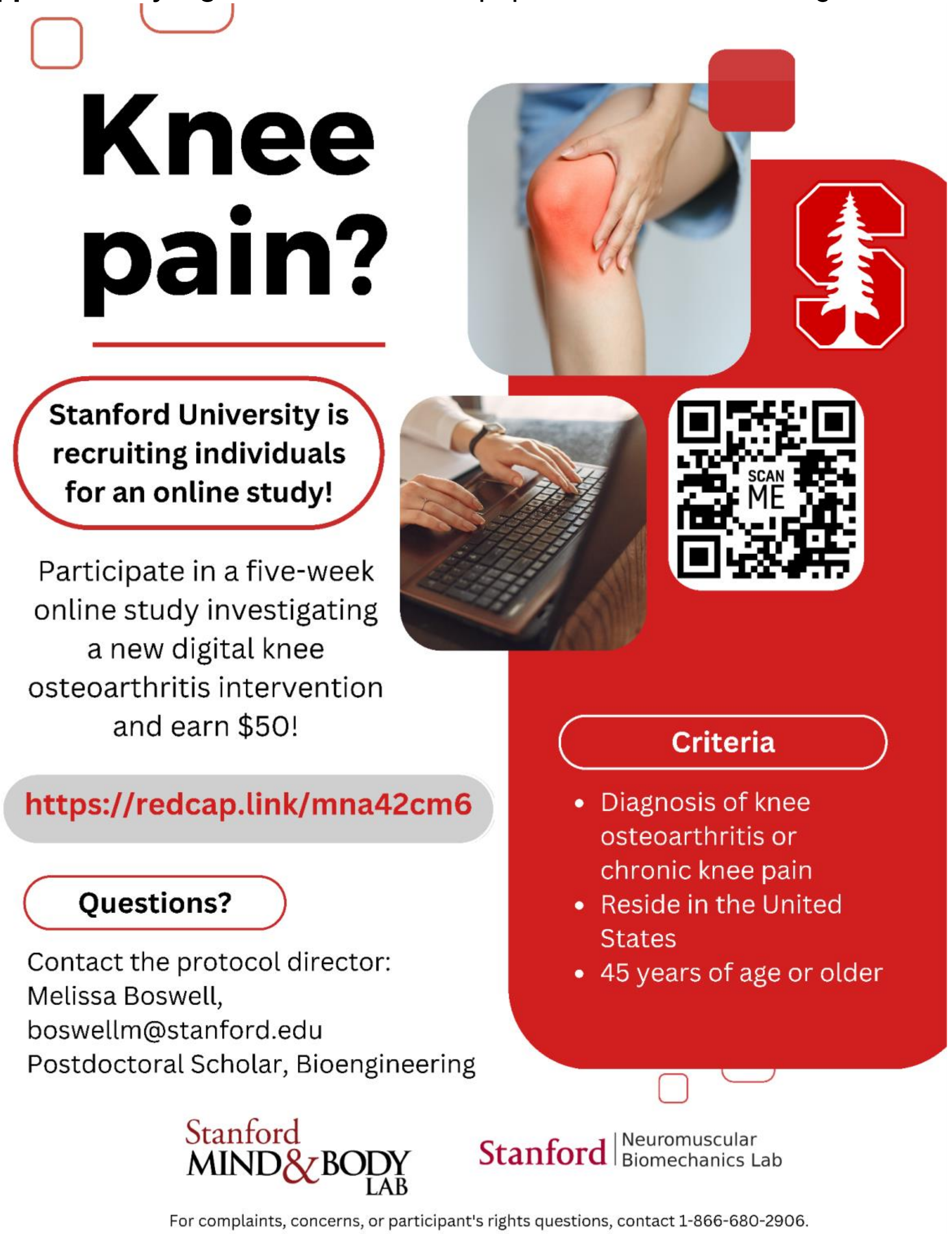

This advertisement for a Stanford University knee pain study includes key details such as study duration, compensation, eligibility criteria, and contact information, alongside images of a knee and a person using a computer. A QR code and website link are provided for easy access to more information about the online study.

Supplementary Note 2. Recruitment email content.

Researchers at Stanford University are recruiting participants with knee osteoarthritis to participate in an online study! Participants will be compensated with up to $50 in Amazon gift cards. Please go to the following link to learn more and see if you qualify: https://redcap.stanford.edu/surveys/?s=3FEY74N4N8NAHPMP

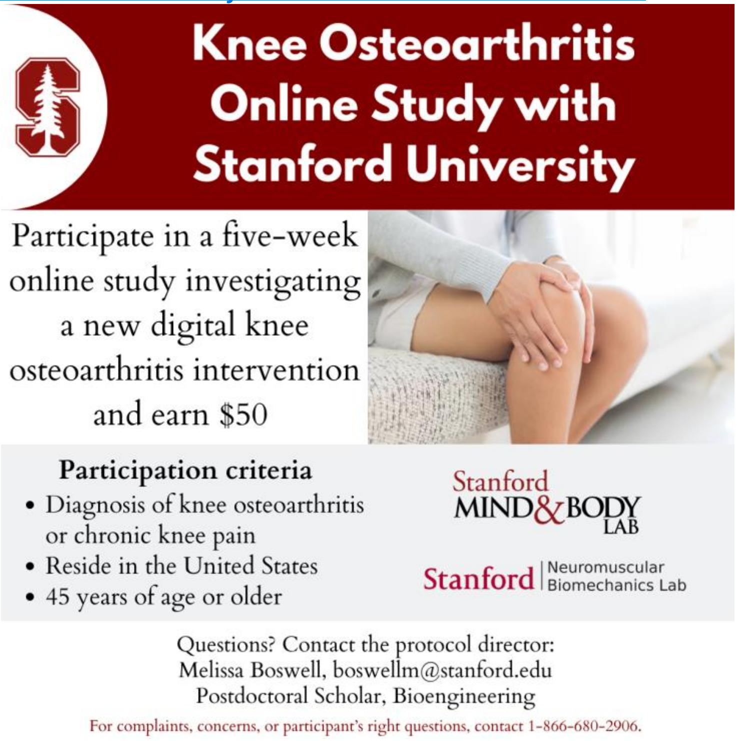

This study lasts five weeks and takes approximately 4 hours in total. During this time:

- You will complete an initial health and demographic survey.
- One week later, you will complete a second survey and may be offered a program including short videos and reflective questions about osteoarthritis.
- One month later, you will be asked to complete a final survey.

Please visit this page to learn more and see if you qualify:
https://redcap.stanford.edu/surveys/?s=3FEY74N4N8NAHPMP

Feel free to share with friends and family with knee osteoarthritis who may be interested in joining.

For study questions, contact Melissa Boswell at boswellm@stanford.edu.
For participant's rights questions, contact 1-866-680-2906.

Thank you for your time and consideration in contributing to research on osteoarthritis!

Supplementary Note 3. Participant Email Communications

Survey Ready Emails

Subject: Survey 1 Ready - Stanford Osteoarthritis Study!

Text:
Hi, ${m://FirstName}!

Welcome! The first part of the Stanford Osteoarthritis Study is ready for you to begin. You have three days to complete Survey 1. This survey will take approximately 15 minutes. Make sure to keep this email, so you can return to your survey at any time by clicking the link below. You can open the link on a computer, smartphone, or tablet.

Follow this link to the Survey:
${l://SurveyLink?d=Take the Survey}

Or copy and paste the URL below into your internet browser:
${l://SurveyURL}

Completing this level will earn you your first $10 gift card. We appreciate your dedication! The study flow below highlights your current study status.

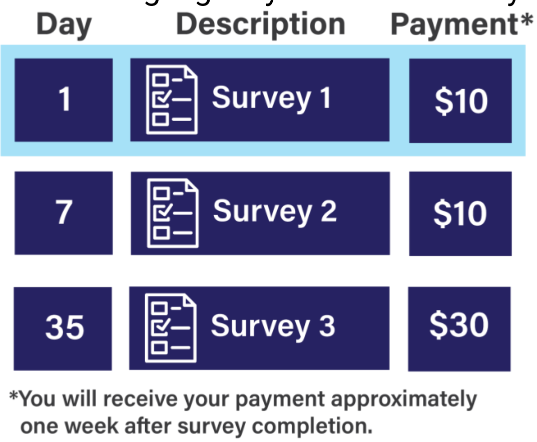

If you have any questions or if you would no longer like to participate in the study, please email the study director, Melissa Boswell, at [email].

Thank you for your time and contributions to osteoarthritis research!
The Stanford Mind & Body Lab

For participant's rights questions, contact [phone].
Follow the link to opt out of future emails:
${l://OptOutLink?d=Click here to unsubscribe}

Subject: Survey 2 Ready - Stanford Osteoarthritis Study!

Text:
Hi, ${m://FirstName}!

Welcome! The second part of the **Stanford Osteoarthritis Study** is ready for you to begin. You have **one week** to complete Survey 2. Make sure to keep this email, so you can return to your survey at any time by clicking the link below. If you close the survey, you will pick back up where you left off by clicking on the link below. You can open the link on a computer, smartphone, or tablet.

Follow this link to the survey:
${l://SurveyLink?d=Take the Survey}

Or copy and paste the URL below into your internet browser:
${l://SurveyURL}

Completing this level will earn you another $10 gift card. We appreciate your dedication! The study flow below highlights your current study status.

Day	Description	Payment*
1	Survey 1	$10
7	Survey 2	$10
35	Survey 3	$30

*You will receive your payment approximately one week after survey completion.

If you have any questions or if you would no longer like to participate in the study, please email the study director, Melissa Boswell, at [email].

Thank you for your time and contributions to osteoarthritis research!

The Stanford Mind & Body Lab

For participant's rights questions, contact [phone].
Follow the link to opt out of future emails:
${l://OptOutLink?d=Click here to unsubscribe}

Subject: Survey 3 Ready - Stanford Osteoarthritis Study!

Text:
Hi ${m://FirstName},

Congratulations! The next part of the Stanford Osteoarthritis Study is ready for you to begin. You will have **three days** to complete Survey 3. This survey will take approximately 15 minutes. Make sure to keep this email, so you can return to your survey at any time by clicking the link below. You can open the link on a computer, smartphone, or tablet.

Follow this link to the Survey:
${l://SurveyLink?d=Take the Survey}

Or copy and paste the URL below into your internet browser:
${l://SurveyURL}

Completing this level will earn your final $30 gift card. We appreciate your dedication! The study flow below highlights your current study status.

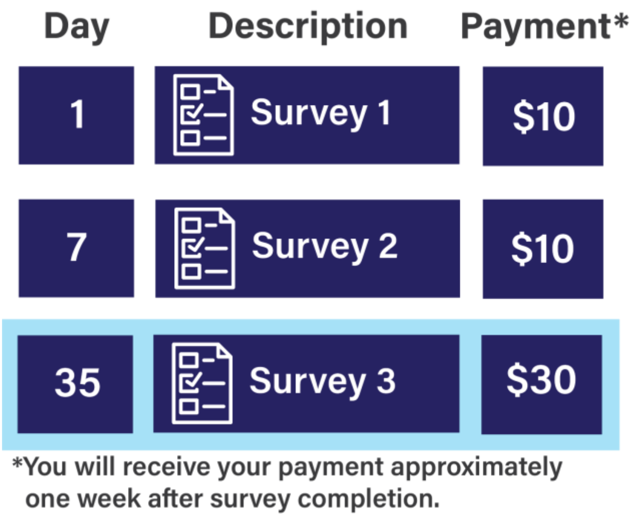

If you have any questions or if you would no longer like to participate in the study, please email the study director, Melissa Boswell, at [email].

Thank you for your time and contributions to osteoarthritis research!

The Stanford Mind & Body Lab

For participant's rights questions, contact [phone].
Follow the link to opt out of future emails:
${l://OptOutLink?d=Click here to unsubscribe}

Survey Reminder Emails

Subject: Survey # Reminder - Stanford Osteoarthritis Study

Text:
Hello, ${m://FirstName}!

This is a friendly reminder to complete Survey [#] of the Stanford Osteoarthritis Study by **tomorrow**.

Follow this link to the Survey:
${l://SurveyLink?d=Take the Survey}

Or copy and paste the URL below into your internet browser:
${l://SurveyURL}

Completing this level will earn you a [$] gift card. Staying on track will keep you progressing and earning additional payments.

The study flow below highlights your current study status:

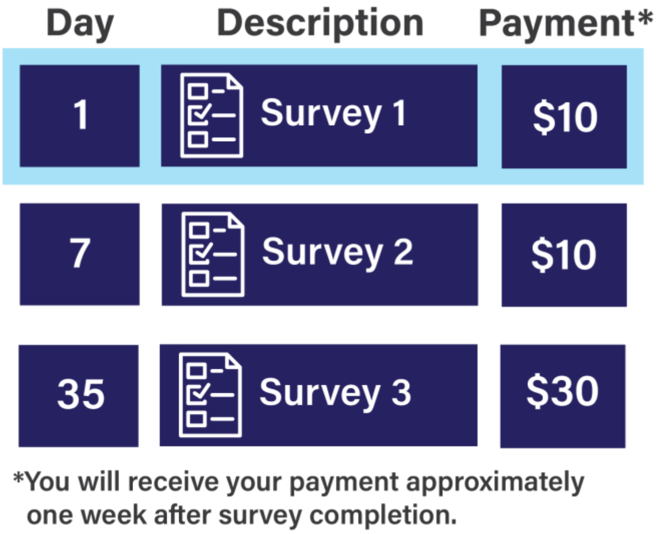

If you have any questions or if you would no longer like to participate in the study, please email the study director, Melissa Boswell, at [email].

Thank you for your time and contributions to osteoarthritis research!
The Stanford Mind & Body Lab

For participant's rights questions, contact [phone].
Follow the link to opt out of future emails:
${l://OptOutLink?d=Click here to unsubscribe}

Subject: Survey # Reminder - Stanford Osteoarthritis Study

Text:
Hi, ${m://FirstName}!

This is a friendly reminder to complete Survey # of the Stanford Osteoarthritis Study by **today**.

Follow this link to the Survey:
${l://SurveyLink?d=Take the Survey}

Or copy and paste the URL below into your internet browser:
${l://SurveyURL}

Completing this level will earn you a [$] gift card. Please note that if you do not complete this survey, you will be removed from the study and will not receive the additional payments.

The study flow below highlights your current study status:

Day	Description	Payment*
1	Survey 1	$10
7	Survey 2	$10
35	Survey 3	$30

*You will receive your payment approximately one week after survey completion.

If you have any questions or if you would no longer like to participate in the study, please email the study director, Melissa Boswell, at [email].

Thank you for your time and contributions to osteoarthritis research!

The Stanford Mind & Body Lab

For participant's rights questions, contact [phone].

Follow the link to opt out of future emails:
${l://OptOutLink?d=Click here to unsubscribe}

Subject: Survey # Final Reminder - Stanford Osteoarthritis Study

Text:
Dear ${m://FirstName},

This is your final reminder to complete Survey # of the Stanford Osteoarthritis Study by today. If you cannot complete the survey by today and want to continue in the study, please let us know by replying to this email. Otherwise, not completing the survey will remove you from the study, and you will not unlock the next surveys or receive gift card compensation.

Follow this link to the Survey:
${l://SurveyLink?d=Take the Survey}

Or copy and paste the URL below into your internet browser:
${l://SurveyURL}

Completing this level will earn you your first $ gift card. Please note that if you do not complete this survey, you will be removed from the study and will not receive the additional payments.

The study flow below highlights your current study status:

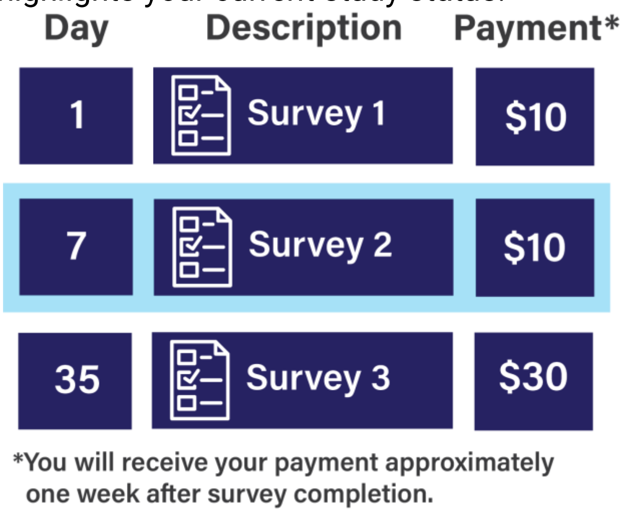

If you have any questions or if you would no longer like to participate in the study, please email the study director, Melissa Boswell, at [email].

Thank you for your time and contributions to osteoarthritis research!

The Stanford Mind & Body Lab

For participant's rights questions, contact [phone].

Follow the link to opt out of future emails:
${l://OptOutLink?d=Click here to unsubscribe}

Intervention Design

Semi-Structured Interviews

We conducted one-on-one semi-structured interviews to uncover narratives that individuals with osteoarthritis hold about osteoarthritis and exercise and how these narratives may relate to their mindsets. Previous research has examined the beliefs individuals with osteoarthritis hold about osteoarthritis, physical activity, and nonsurgical intervention[16–18], including how they are formed and their impact [19]. As these studies primarily focus on misconceptions about osteoarthritis and the barriers to physical activity, a gap remains in understanding the views and beliefs of individuals with osteoarthritis who still enjoy physical activity. This understanding is critical to influencing mindsets; we must offer more beneficial ways to view osteoarthritis and exercise rather than simply addressing problematic views. Thus, our goal was to acquire tangible takeaways, such as key language and themes, that could be directly implemented in and shape the narrative of our intervention.

We recruited participants from a convenience sample of patients with knee osteoarthritis involved in a large clinical trial on campus and through word-of-mouth. We selectively invited individuals to interview who participated in regular physical activity and whom we hypothesized to have an adaptive mindset about exercise (i.e., they enjoy exercise). The first author (MAB) worked with PLM, an expert in narrative theory, to develop an interview guide with prompts to elaborate responses (**Supplementary 4 Note 4**). The guide consisted of questions about osteoarthritis diagnosis, the impact and management of osteoarthritis, and experience with exercise. MAB carried out the interviews via Zoom and had neither directly met nor had previous conversations with the participants before the interview. The interviews were audio recorded and transcribed verbatim for analysis.

Supplementary Note 4. Interview guide

Diagnosis

1. **Think about the first time you suspected that something was wrong with your knee.**
    a. When was it?
    b. Where was it?
    c. Who were you with?
    d. How did it make you feel?
    e. How did you make sense of it at the time?
2. **Recall the first time you were told by a healthcare professional that you had knee osteoarthritis.**
    a. When did it take place?
    b. Where did it take place?

c. What kind of health professional (general practitioner, specialist, nurse practitioner) gave you the diagnosis?
d. Did you trust him/her/them?
e. How did it make you feel?

3. **Think about how the healthcare professional who diagnosed your osteoarthritis presented it to you.**
    a. Did they use any specific images to explain the disease to you?
    b. Do you still think of it in those images or terms or have you found a better way to explain it to yourself?
    c. Had you heard about osteoarthritis before you were diagnosed?
    d. From who/where?
    e. What did you know about it/how did you view it before you were diagnosed?
    f. What do you think is the cause or reason for your osteoarthritis, if any?

Impact and Management

1. **How has osteoarthritis affected your life?**
    a. Does it keep you from doing anything you used to do?
    b. Has it motivated you to do anything you didn't do before?
    c. Have your experiences with osteoarthritis gotten worse or better over time?
2. **Do you talk with your friends or family about osteoarthritis?**
    a. How, if at all, does what they say influence how you think about osteoarthritis?
3. **What things (medication, meditation, exercise, resting, etc.) improve osteoarthritis symptoms in general?**
    a. Which of these (if any) do you use to try to cope with or manage your osteoarthritis?
    b. Do you think these impact your symptoms?

Exercise

1. **How much exercise, in general, do you think you should be getting?**
    a. How do you think the amount of exercise you are getting compares to what you just described?
2. **What types of exercise should you be doing to help your osteoarthritis?**

   a. How do you think the exercises you do compare to what you just described?

3. **How would you describe exercising?**
   a. What do you enjoy about exercising?
4. **What do you think are your most significant barriers to exercising?**
   a. Do you think having osteoarthritis has affected your willingness to be physically active?

Before the interviews, participants completed a questionnaire including demographics and the Process of Health Mindset -- Exercise survey[1]. The Process of Health Mindset -- Exercise (referred to as the "exercise mindset") is a 7-item scale measured on a 4-point scale and scored from 1 to 4, with a higher score reflecting a more appeal-focused mindset about physical activity.

Ten participants with a self-reported diagnosis of knee osteoarthritis and no history of knee surgery were interviewed (**Supplementary Table 5**). Confirming our hypothesis, the average exercise mindset of participants was over one standard deviation greater than the average exercise mindset of individuals with knee osteoarthritis (mean 3.0, standard deviation (SD) 0.4 vs. mean 2.2, SD 0.7, respectively). MAB and PLM became independently familiar with transcripts. They agreed upon a coding framework comprising three themes (patterns associated with three or more interviews) regarding the cause and prognosis of osteoarthritis, five regarding impact and management, and seven regarding exercise (**Supplementary Figure 8**). MAB applied this coding framework to all transcripts, and the discovered patterns and themes were discussed among the MAB, PLM, KME, and AJC.

Supplementary Table 4. Participant characteristics of the semi-structured one-on-one interviews (N=10).

Characteristics	Value
Age (years), mean (SD; range)	62.2 (6.6; 51-73)
BMI (kg/m^2), mean (SD; range)	26.8 (2.8; 24.0-34.0)
Sex, n (%)	
Male	6 (60%)
Female	4 (40%)
Ethnicity, n (%)	
Hispanic or Latinx	0 (0%)
Not Hispanic or Latinx	10 (100%)
Race, n (%)	
White	10 (100%)
Education, n (%)	
High school graduate	1 (10%)
College degree or higher	9 (90%)
Employment, n (%)	
Employed full-time	4 (40%)
Employed part-time	1 (10%)
Retired	5 (50%)
Marital Status, n (%)	
Married	8 (80%)
Widowed	2 (20%)
Time since KOA diagnosis (years), mean (SD; range)	6.0 (7.4; 1-25)
KOA symmetry, n (%)	
Unilateral	3 (30%)
Bilateral	7 (70%)

Supplementary Figure 8. Emergent themes from the semi-structured one-on-one interviews.

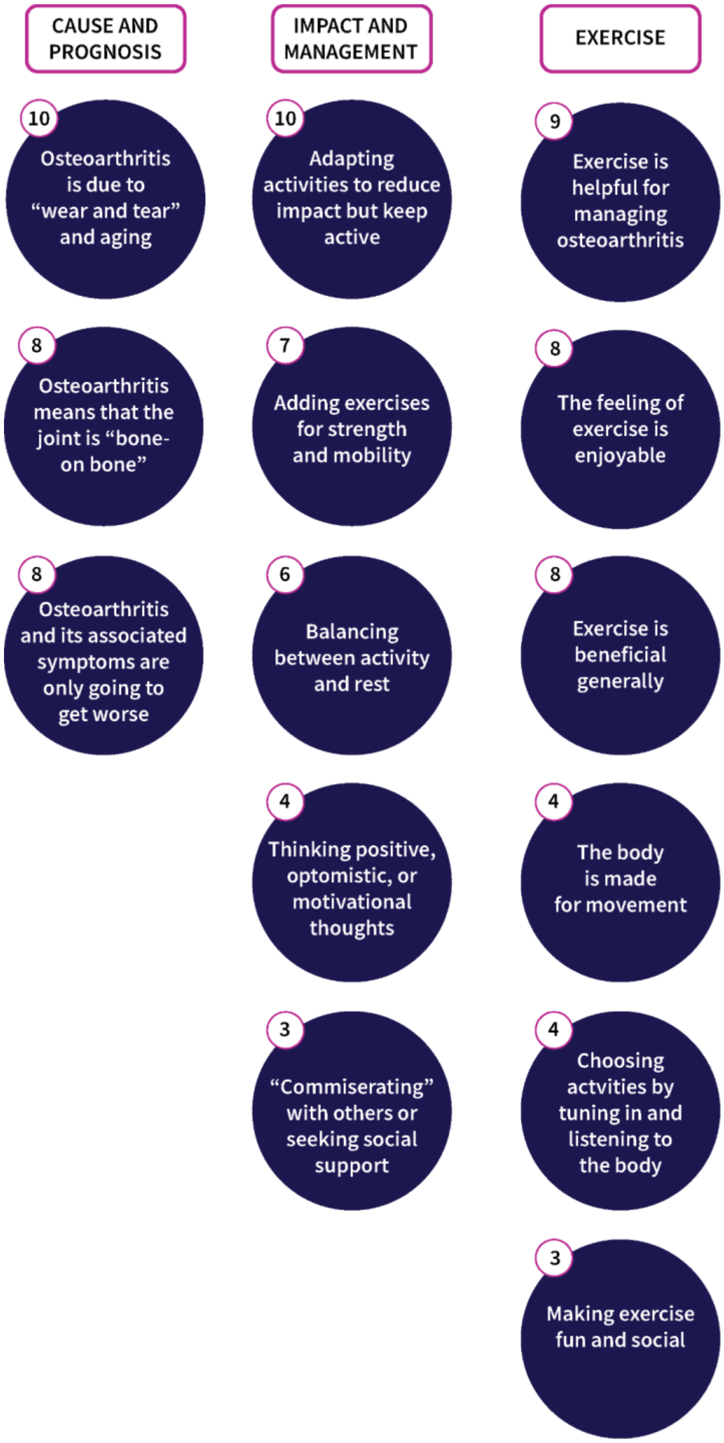

In this figure, each column represents a specific area with each blue circle as an emerging theme. The pink circle indicates the number of participants (out of 10) who expressed sentiments aligned with the determined theme.

Similar to previous studies[16,19], all participants noted “wear and tear” and aging as the cause of osteoarthritis, most (8 participants) believed that osteoarthritis means the knee is “bone-on-bone,” and most (8 participants) expressed worry of inevitable degradation and worsening symptoms. Also consistent with previous studies, participants highlighted managing osteoarthritis through a balance of movement and rest[19] and altering their activity amount and type[18]. Participants also mentioned positive thinking, leaning on social support, and ignoring the pain.

We uncovered several themes about exercise that contribute to ongoing participation. Participants mentioned the benefits of exercise generally (e.g., healing and building confidence) and specifically for osteoarthritis (e.g., improving strength and mobility). They highlighted enjoying the feeling of exercise (e.g., fun, social, playful, meditative, and euphoric). Participants also noted the importance of tuning into the body and how the body is meant for movement. A couple of participants (though not considered a “theme” amongst participants) recognized the difficulty in getting started with exercise despite the benefits and enjoyable feeling after getting started, which was consistent with previous studies[17].

Overall, while these active individuals had common misconceptions about osteoarthritis, their views and beliefs about exercise as enjoyable and beneficial seemed to have a greater effect on their continued participation in exercise. These patient views and beliefs, and their associated language, were used to inform mindset theory in the context of osteoarthritis and for intervention development.

Mindsets Related to Osteoarthritis

Using knowledge about beliefs about osteoarthritis and exercise and how they are shaped[16–19], along with our semi-structured interviews, we developed a theory of mindsets in the context of having osteoarthritis (**Supplementary 4 Figure 9**). This theory includes how mindsets are shaped, the mindset itself, and how mindsets influence behavior, attention, feelings, and physiology. One strategy to intervene upon mindsets is to attempt to convince individuals to adopt a more beneficial mindset because the beliefs surrounding that mindset are true, while not necessarily teaching about mindsets[20]. A second strategy, the meta-mindset approach, is to explicitly teach individuals about the power of mindsets and how to harness them in the way that best serves their lives[21]. Interventions lacking this meta-cognitive element may be less likely to have long-term effects in individuals with osteoarthritis as this population is faced with challenges like knee pain, which may feel in opposition to the belief that exercise is fun or osteoarthritis is manageable. Thus, our intervention takes a meta-mindset approach, which we hypothesized would empower individuals to adopt mindsets that benefit their osteoarthritis status and overall health and well-being.

Supplementary Figure 9. A diagram theorizing osteoarthritis and exercise mindsets.

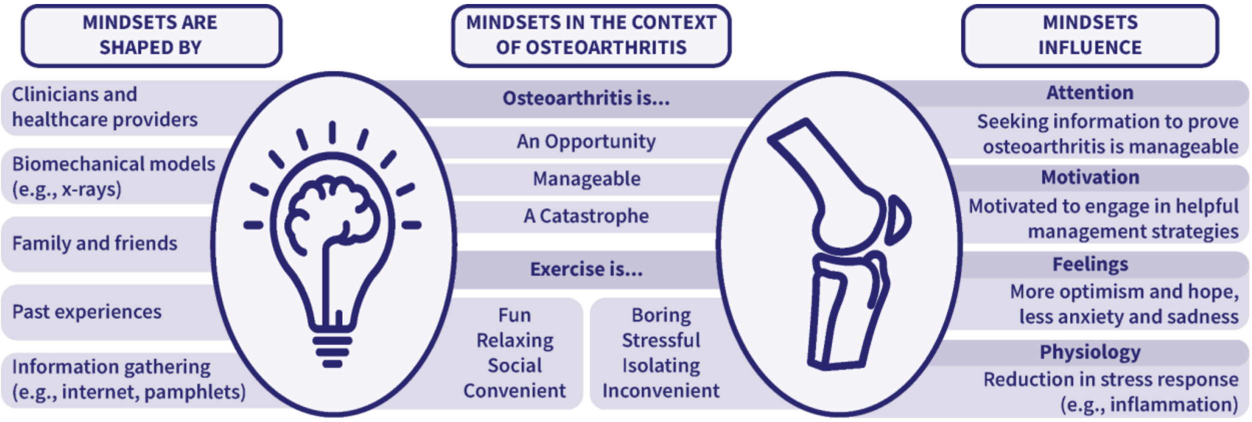

The mindsets are presented in the context of osteoarthritis, how those mindsets are shaped, and how they may influence an individual. The examples of influence are based on the mindset that osteoarthritis is manageable.

Multidisciplinary Expertise

The development of our intervention video scripts went through many cycles of iteration with feedback from a set of multidisciplinary experts. Their expertise spanned bioengineering, biomechanics, mechanical engineering, psychology, narratology, and orthopedic surgery. A subset of these experts participated in the recording of our intervention. We also asked patients to review the scripts, input their own experiences, and participate in the recording of the films.

Intervention Pilot Testing

Overview
The pilot study aimed to collect feedback about our mindset intervention, *Rethinking Osteoarthritis*, from individuals with knee osteoarthritis. While we did not expect to have enough individuals to power detecting significant changes in mindset, we aimed to verify that changes in mindset were trending in a positive direction before launching a larger trial.

Methods and Participants
We recruited participants for pilot testing via social media advertising. The advertisement link directed individuals to an online survey platform (Qualtrics, Provo, UT, USA), where they received an overview of the study, screened for qualification, and provided electronic informed consent if they qualified. Participants had one week to complete the *Rethinking Osteoarthritis* program at their own pace.

Measures
Immediately before and after the program, participants completed the following measures: (i) Exercise mindset using the Mindset about the Process of Health – Exercise scale; (ii) Osteoarthritis and body mindsets using the Illness Mindset Inventory; (i) Knee osteoarthritis knowledge by the Knee Osteoarthritis Knowledge Scale. Participants provided the following characteristics prior to the program: age; sex; gender; BMI from height and weight; race; education; employment; comorbid conditions; unilateral vs. bilateral osteoarthritis presentation; the time since their knee pain started; and the time since they were diagnosed with knee osteoarthritis. Inclusion and exclusion criteria were consistent with the criteria described for the clinical trial. Participants were also asked open-ended and Likert Scale feedback questions following each module and the entire program (**Supplementary Note 5**).

Supplementary Note 5. Open-ended feedback questions following each module and the entire program during the pilot study.

Open-Ended Feedback Questions

1. What, if anything, did you find confusing or unhelpful about this [module/program] as a whole?
2. What, if anything, did you find exciting or helpful about this [module/program] as a whole?
3. Do you have any suggestions for improving this [module/program] or making it more helpful?
4. Do you have any additional comments about this [module/program]?

Likert Scale Feedback and Motivational Questions

Answer options: Strongly disagree (1), Disagree (2), Neither agree nor disagree (3), Agree (4), or Strongly agree (5)

1. The information and questions presented in this program were:
    a. Enjoyable
    b. Helpful
    c. Relevant
    d. New information
    e. Accurate
2. I would recommend this program to friends and family with osteoarthritis
3. I find mindsets to be a valuable tool for improving my experience with osteoarthritis
4. I am motivated to find ways to manage my osteoarthritis after watching this program
5. I am motivated to increase my physical activity after this program
6. I am motivated to find ways of making physical activity more enjoyable after this program
7. I am motivated to adopt the mindset that osteoarthritis is manageable
8. I am motivated to adopt the mindset that exercise is enjoyable

Statistical Analysis
Summary scores were calculated for each measure before and after the intervention. Pre- and post-intervention differences were assessed with *t*-tests.

Results
A total of 21 participants completed the online program **(Supplementary Table 5)**. An additional three participants started the program but did not finish it. Post-intervention, participants had higher scores in knee osteoarthritis knowledge (P<0.001), and all mindset measures changed in the anticipated direction (P<0.001; **Supplementary Figure 10**; **Supplementary Table 6**). Furthermore, participants indicated high scores across the program feedback and motivational Likert-scale questions (**Supplementary Table 8**).

Supplementary Table 5. Participant characteristics of the semi-structured one-on-one interviews (N=21).

Characteristic	mean ± SD (min, max)
Age (years)	63.52 ± 5.4 (53, 71)
Sex, n (%) female	19 (90%)
BMI (kg/m2)	29.0 ± 6.4 (20.1, 44.0)
Ethnicity, n (%)	
White	17 (81%)
Black or African American	1 (5%)
Hispanic or Latino or Spanish Origin	2 (9%)
Mixed race	1 (5%)
Time since KOA diagnosis (years), mean (SD; range)	4 (0.5; 19)
Abbreviations: BMI, Body Mass Index; KOA, Knee osteoarthritis	

Supplementary Figure 10. Box-and-whisker plots of scores before (Pre) to after (Post) participating in the mindset intervention (N=21), as determined by a t-test.

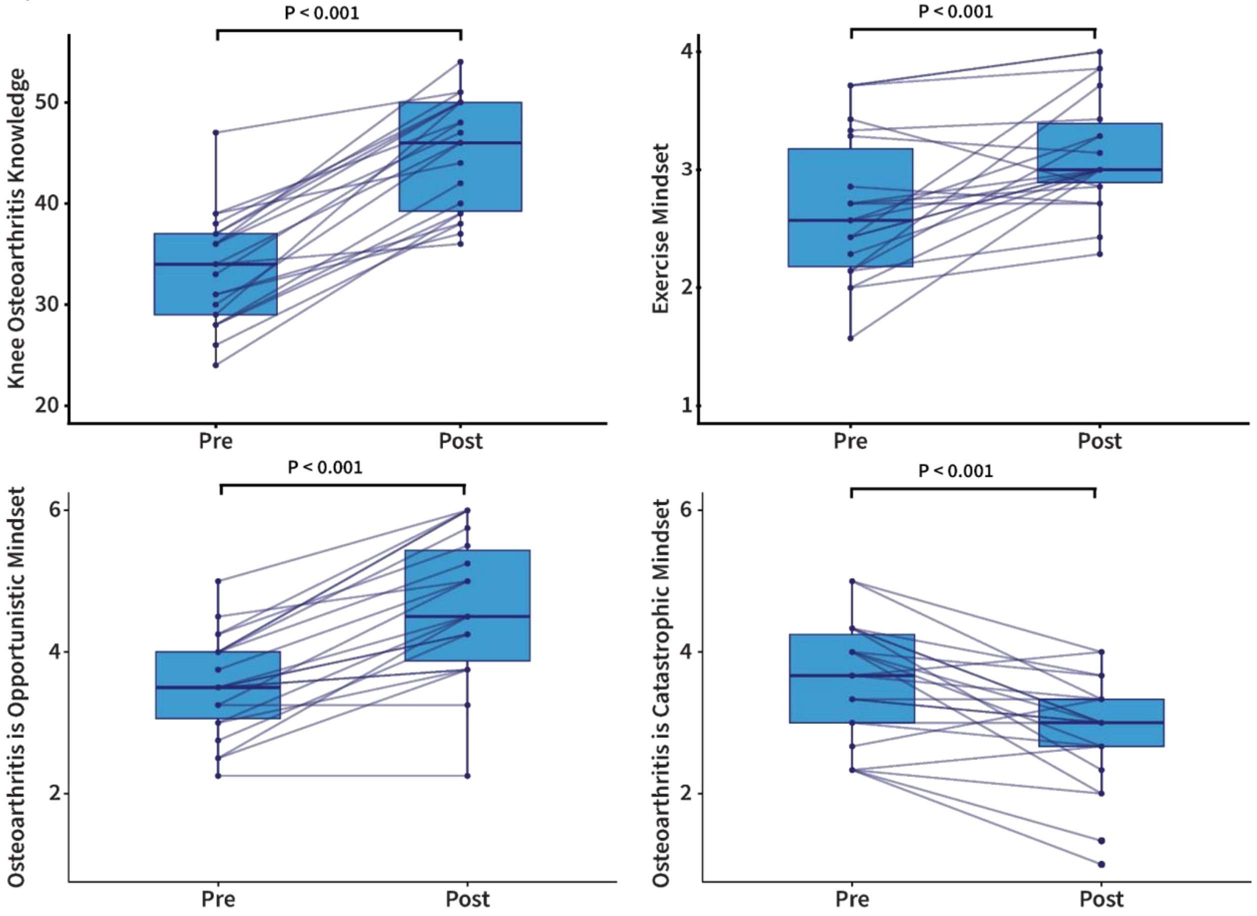

In the box-and-whisker plots, the top and bottom lines of the boxes (hinges) are the first and third quartiles, respectively. The horizontal line is the median, and the whiskers extend from each hinge to the minimum and maximum values.

Supplementary Table 6. Summary measures and estimated mean difference in change [95% CI].

Measure	Pre-Intervention mean (SD)	Post-Intervention mean (SD)	Mean difference [95% CI]	P-value
Knee Osteoarthritis Scale	33.4 (5.4)	45.0 (5.5)	11.6 [9.4, 13.2]	<0.001
Exercise Mindset	2.7 (0.6)	3.2 (0.5)	0.5 [0.2, 0.7]	<0.001
Osteoarthritis is Catastrophic Mindset	3.6 (0.8)	2.9 (0.8)	-0.7 [-1.0, -0.4]	<0.001
Osteoarthritis is Manageable Mindset	3.9 (0.7)	5.0 (0.8)	1.3 [0.8, 1.5]	<0.001
Osteoarthritis is Opportunistic Mindset	3.5 (0.7)	4.7 (1.0)	1.1 [1.4, 0.8]	<0.001
The Body is Adversarial Mindset	3.0 (1.0)	2.2 (0.9)	-0.8 [-1.1, -0.4]	<0.001
The Body is Capable Mindset	3.4 (0.6)	4.6 (0.9)	1.2 [0.8, 1.6]	<0.001
The Body is Resilient Mindset	4.2 (0.7)	4.7 (0.8)	0.6 [0.3, 0.9]	<0.001

Supplementary Table 7. Summary scores of the program reaction and motivation-focused questions. Questions are ranked on the Likert scale of strongly disagree (1) to strongly agree (5) unless otherwise indicated.

Question	Mean (SD)
The information and questions presented in this program were accurate.	4.6 (0.7)
The information and questions presented in this program were enjoyable.	4.7 (0.5)
The information and questions presented in this program were helpful.	4.7 (0.5)
The information and questions presented in this program were new information.	4.4 (0.9)
The information and questions presented in this program were relevant.	4.5 (0.8)
I would recommend this program to friends and family with osteoarthritis.	4.8 (0.5)
I find mindsets to be a valuable tool for improving my experience with osteoarthritis.	4.6 (0.8)
I am motivated to find ways to manage my osteoarthritis after watching this program.	4.7 (0.7)
I am motivated to increase my physical activity after this program.	4.8 (0.5)
I am motivated to find ways of making physical activity more enjoyable after this program.	4.7 (0.6)
I am motivated to adopt the mindset that osteoarthritis is manageable.	4.7 (0.5)
I am motivated to adopt the mindset that exercise is enjoyable.	4.7 (0.5)
Overall, how would you rate the quality of this program?<br>(1 = very poor, 5 = very high)	4.6 (0.6)

Open-ended participant feedback was overall positive (e.g., “I'm excited to get started with changing my mindset and with physical activity. I want to see if it will truly make a difference. I would rather start this way than with surgery the doctor recommends,” “A new way of thinking and living! I am in awe of the presentations and new way of thinking about both exercise and OA! Wow!!”). Most participants stated, “Nothing” when asked if anything was unhelpful or confusing. Two participants mentioned that one of the speakers (an individual with knee osteoarthritis) was sometimes confusing. Because of this, we trimmed that speaker’s lines in the final videos. No further refinement or changes were made.

Intervention Content

Supplementary Note 6. Mindset Intervention Content*

***Note: Associated videos are available upon request.**

Program Introduction

Welcome to **Rethinking Osteoarthritis**!

In this program, we will offer important insights into what it means to have osteoarthritis, exercise with osteoarthritis, and use the power of your mind and body. Throughout the program, we will introduce you to world-leading experts in orthopedic surgery, osteoarthritis, physical activity, and psychology at Stanford University, including:

- **Bioengineering researcher**, Melissa Boswell, PhD
- **Orthopedic surgeon**, Nick Giori, MD, PHD
- **Bioengineering professor and researcher**, Scott Delp, PhD
- **Psychologist and professor**, Alia Crum, PhD
- **Health Researcher and Coach**, Kris Evans

We will start by breaking down the common view of osteoarthritis as a limiting disease. Then, we will rebuild a view of having osteoarthritis as a manageable, even empowering, experience. After, we will explore how physical activity can be helpful and enjoyable. Finally, we will share how your mindset plays an influential role in your decisions to manage your osteoarthritis and your health. By the end of this program, we hope you'll have tangible and meaningful ways to cultivate a mindset that supports you!

This program has **four modules**, each with a series of short films and reflective activities. Each module will take approximately **20-60 minutes** to complete. You have **one week** to complete all modules of the program at your own pace. Our suggestion is to complete one module per day, but we encourage you to go at the pace that works best for you – whether that's faster or slower!

You will have access to the content in this program at any time. Feel free to discuss the content with friends and family or watch the videos with them!

We hope that **Rethinking Osteoarthritis** will positively impact your experience with osteoarthritis and your life.

Continue to begin Module 1!

Module 1: Understanding Osteoarthritis

Introduction

Welcome to Module 1 of **Rethinking Osteoarthritis**!

In this module, we will introduce new research that helps us better understand osteoarthritis and common misconceptions. In the end, you will have new and beneficial ways to view osteoarthritis and your own experience with it. We hope this knowledge will help you feel more empowered to manage your osteoarthritis.

This module will be guided by **bioengineering researcher**, Melissa Boswell, MS, and **orthopedic surgeon**, Nick Giori, MD, PhD. It consists of four short films, followed by reflection questions on what you've just learned.

Part 1: Fact #1: Having osteoarthritis doesn't necessarily mean your joint is "bone-on-bone"

VIDEO: Rethinking Osteoarthritis: Module 1 | Part 1

Reflection Questions

1. Every diagnosis of osteoarthritis means that there is bone-on-bone contact in the joint. (T/F)
    a. True: Not exactly! In this video, we explained that osteoarthritis is a diagnosis that covers a spectrum of changes in one's cartilage and doesn't necessarily mean bone-on-bone. Remember that regardless of the state of your osteoarthritis, you can still slow additional wear!
    b. False: That's right! In this video, we explained that osteoarthritis is a diagnosis that covers a spectrum of changes in one's cartilage and doesn't necessarily mean bone-on-bone. Remember that regardless of the state of your osteoarthritis, you can still slow additional wear!

2. What images came to mind when thinking about or visualizing your joint **before** watching this video?

3. What images or visualizations of your joint do you think will be most helpful **after** watching this video?

Part 2: Fact #2: You can improve joint health and slow the progression of osteoarthritis

VIDEO: Rethinking Osteoarthritis: Module 1 | Part 2

Reflection Questions

1. Osteoarthritis means a progressive decline in health. (T/F)
    a. True: Not quite! You can do many things to improve your symptoms, joint function, and overall health - all of which slow osteoarthritis progression!
    b. False: That's correct! You can do many things to improve your symptoms, joint function, and overall health - all of which can slow osteoarthritis progression!

2. What is one thing you could try in your life to improve your joint health?

Part 3: Fact #3: Everyday physical activity will not worsen your joint health

VIDEO: Rethinking Osteoarthritis: Module 1 | Part 3

Reflection Questions

1. Because osteoarthritis is due to wear and tear, it will worsen with everyday activity. (T/F)
    a. True: Not quite! You can do many things to improve your symptoms, joint function, and overall health - all of which help to slow osteoarthritis progression!
    b. False: That's correct! You can do many things to improve your symptoms, joint function, and overall health - all of which help to slow osteoarthritis progression!

2. How does reframing "wear and tear" to "wear and repair" change the way you view physical activity as it relates to your joint health and osteoarthritis?

Part 4: Fact #4: Pain doesn't mean damage or that you need to stop being active

VIDEO: Rethinking Osteoarthritis: Module 1 | Part 4

Reflection Questions

1. Pain due to osteoarthritis means I should stop moving. (T/F)

    a. True: That’s not exactly right. Pain is complex and due to many factors, not just the physical state of your joint. Movement actually improves the strength, mobility, and function of your joints, so you should make sure to stay active!
    b. False: You got it! Pain is complex and due to many factors, not just the physical state of your joint. Movement actually improves the strength, mobility, and function of your joints, so you should make sure to stay active!

2. Some people with osteoarthritis find it helpful to repeat affirmations about their pain when they experience it. For example, one thing you might tell yourself when experiencing pain is, “Sometimes I experience pain, but it doesn’t have control over me. I have moved through pain in the past and can do it again.”

   What is an affirmation you can tell yourself that will help you get through times where you may feel pain?

3. As we mentioned in the video, activities that reduce your stress will also reduce your inflammation, even in your joints! What is an activity that brings you joy that can help you relax in times of pain?

Closing

In this module, we learned:

1. Having osteoarthritis doesn’t necessarily mean your joint is “bone-on-bone.”
2. You can improve joint health and slow the progression of osteoarthritis.
3. Everyday physical activity will not worsen your joint health. Think wear and repair!
4. Pain doesn’t always mean damage or that you need to stop being active.

Instead of focusing on the structure of the cartilage in your joints, which you can’t control, try to focus on the parts of your joint health that can be improved, like muscle strengthening and mobility, along with other things that influence pain. That way, you can use your energy to improve what you can change, which will help you recover and improve your life, even with joint changes.

Module 1 References

1. Odding, E., Valkenburg, H. A., Algra, D., Vandenouweland, F. A., Grobbee, D. E., & Hofman, A. (1998). Associations of radiological osteoarthritis of the hip and knee with locomotor disability in the Rotterdam study. Annals of the Rheumatic Diseases. https://doi.org/10.1136/ard.57.4.203
2. Butler, D.S. & Mosely, G.L. (2013). Explain Pain. Adelaide: Noigroup Publications.

Module 2: Move to Improve

Introduction

Welcome to Module 2 of **Rethinking Osteoarthritis**! So far, we debunked common misconceptions about osteoarthritis and learned the facts. In this module, we'll dive deeper into physical activity. You'll learn what type of exercise is beneficial for improving osteoarthritis symptoms and joint health and the many benefits of movement.

In this module, we'll provide simple yet tailored physical activity guidelines. We'll explore the most recent scientific evidence about the many benefits of exercise. Finally, we will highlight how your body is capable and resilient and how movement supports this. The purpose of this module is to stimulate a new appreciation for your body and the positive effects of physical activity.

This module will be guided by **bioengineering researcher**, Melissa Boswell, MS, and **bioengineering professor and researcher**, Scott Delp, PhD. It consists of three short films, followed by reflection questions on how the learnings apply to your life.

Part 1: Physical Activity for Osteoarthritis Management

VIDEO: Rethinking Osteoarthritis: Module 2 | Part 1

Reflection Questions

1. What type of activities are most helpful for improving osteoarthritis symptoms?
    a. [Open Response]
    b. Awesome! While any activity is better than none, low to moderate exercise is best for improving symptoms and slowing osteoarthritis progression. Low to moderate activity can include:
        i. Walking, gardening, biking, stretching, yoga, and strength training.

2. In this module, we learned about the benefits of incorporating short bouts of physical activity throughout your day. Some ideas for this include:
    a. Taking a walk around the block after a meal
    b. Parking a little farther from the store to get in some extra steps
    c. Doing some stretching while listening to the news or watching TV

What is one way that you could try to add a short bout of physical activity to your day?

Part 2: The Benefits of Movement

VIDEO: Rethinking Osteoarthritis: Module 2 | Part 2

Reflection Questions

1. What advice would you give someone with osteoarthritis who believes that to "protect" their joints, they should stop being physically active?
    a. Great! The best way to protect your joints is to keep them strong and mobile through movement.

2. List one or more benefits of physical activity that are surprising or exciting to you.
    a. Fantastic! We mentioned many benefits, including:
        i. Improved pain and stiffness
        ii. Improved joint strength and mobility
        iii. Improved balance and stability
        iv. Improved protection against many diseases
        v. Improved mood and mental health
        vi. Improved creativity and social interactions

Part 3: Your Body is Capable and Resilient

VIDEO: Rethinking Osteoarthritis: Module 2 | Part 3

Reflection Questions

Let's reflect on how your body is capable and resilient. Our bodies adapt with movement and challenges such as improvements in strength, flexibility, balance, or endurance. This adaptation happens no matter our age or fitness level! Can you think of a time your body positively adapted or responded to challenges it was faced with?

Closing

In this module, we learned that:

- Low to medium impact activities and shorter bouts of exercise spread throughout the day are well-suited for people living with osteoarthritis.
- Movement improves osteoarthritis symptoms and overall health and wellbeing, including physical, mental, and emotional benefits.
- Our bodies are capable and resilient, and movement amplifies this, no matter our bodies' shape, size, or condition.

Module 2 References

1. McGonigal, Kelly. 2019. The Joy of Movement. Book. Penguin Publishing Group.

2. Oppezzo, Marily, Schwartz, Daniel L. 2014. Give your ideas some legs: The positive effect of walking on creative thinking. Journal of Experimental Psychology: Learning, Memory, and Cognition.
3. Maher et al. 2014. Daily satisfaction with life is regulated by both physical activity and sedentary behavior. Journal of Sport and Exercise Psychology.
4. Young et al. 2018. The Cascade of Positive Events: Does Exercise on a Given Day Increase the Frequency of Additional Positive Events?” Personality and Individual Differences.
5. Puterman et al. 2017. Physical Activity and Negative Affective Reactivity in Daily Life. Health Psychology

Module 3: Enjoying Exercise

Introduction

Welcome to Module 3 of **Rethinking Osteoarthritis**! In the previous modules, we broke down misconceptions about osteoarthritis. We also learned about the amazing benefits of physical activity, including how it helps with osteoarthritis and challenges our bodies to be even more capable and resilient. While all of this is useful, sometimes exercise turns into something you feel you *have* to do rather than something you *want* to do.

In this module, we will provide an opportunity for you to rethink physical activity as something that you want to do because it is fun, social, and relaxing as opposed to boring or stressful. We hope you feel encouraged to view physical activity in a more nourishing and enjoyable light by the end of this module!

This module will be guided by bioengineering researcher, Melissa Boswell, MS, and health researcher and coach, Kris Evans. It consists of three short films, followed by reflection questions on how the learnings apply to your life.

Part 1: Exercise can be relaxing and pleasurable

VIDEO: Rethinking Osteoarthritis: Module 3 | Part 1

Reflection Questions

1. Let's reflect on what came to mind when you thought about exercise **before** watching this video. What activities came to mind? How did you expect it should feel?

2. How have your ideas of exercise changed **after** watching this video?

3. What are three ways that you can make physical activity more pleasurable or relaxing?

Part 2: Exercise can be convenient and easy

VIDEO: Rethinking Osteoarthritis: Module 3 | Part 2

Reflection Questions

1. What advice would you give someone with osteoarthritis who believes that vigorous exercise, such as running and jump roping, is the only type of physical activity that "counts" as exercise?

2. What is one way you are getting physical activity in your daily life that you might not have considered previously as a form of exercise?

3. It can also be important to notice and appreciate the exercise you already do. Try to think of some of the activities you already do on a daily or weekly basis that you can think about as exercise (e.g., gardening, walking, shopping, etc.).

4. What are three ways that you can make physical activity more easy or convenient?

Part 3: Exercise can be fun, social, and indulgent

VIDEO: Rethinking Osteoarthritis: Module 3 | Part 3

Reflection Questions

1. What is one way you can make your exercise more fun?

2. What is one way you can make your exercise more social?

3. What is one way you can make your exercise more indulgent?

Great! Over the next few days, challenge yourself to do one or more of your ideas to make exercise more pleasurable, relaxing, easy, convenient, fun, social, or indulgent.

Closing

In this module, we encourage you to explore exercising in a way that feels:

- Pleasurable and relaxing
- Easy and convenient
- Fun, social, and indulgent

Finding ways to make exercise an enjoyable and valuable part of your life can help you take advantage of it as a way to improve your osteoarthritis symptoms and your overall mental and physical health so that you can continue to live an active and fulfilling life.

We hope that this module empowers you to be creative with exercise and find ways to move that feel meaningful to you.

Module 3 References

1. McGonigal, Kelly. 2019. The Joy of Movement. Book.

Module 4: Mindsets Matter

Introduction

Welcome to Module 4 of **Rethinking Osteoarthritis**! In the previous modules, we broke down misconceptions about osteoarthritis, shared the benefits of physical activity, and provided new ways to view exercise as enjoyable. Ultimately, we were considering the ways we think about osteoarthritis and exercise. These thoughts and beliefs make up your *mindset*.

In this final module, we will talk about what mindsets are, why they are important, and how you can adopt more helpful mindsets about osteoarthritis and exercise. Since you can control your mindset, it makes it one of the most helpful tools for managing osteoarthritis - or any challenge in your life!

This module will be guided by **bioengineering researcher**, Melissa Boswell, MS, **psychologist and professor**, Alia Crum, PhD, and **health researcher and coach**, Kris Evans. It consists of two short films, followed by reflection questions on how the learnings apply to your life.

Part 1: What are mindsets and why do they matter?

VIDEO: Rethinking Osteoarthritis: Module 4 | Part 1

Reflection Questions

1. What are the features of mindsets? Please check all of the following features that apply.
    - Every person has mindsets
    - Mindsets are simplified versions of reality
    - Mindsets can shape our well-being, health, and behavior
    - Mindsets are influenced by our culture
    - Mindsets don't matter at all

A: Mindsets have many of the features that were listed, including:

- Every person has mindsets
- Mindsets are simplified versions of reality
- Mindsets can shape our well-being, health, and behavior
- Mindsets are influenced by our culture

2. In which ways might the mindset "osteoarthritis is manageable" influence a person? Please check all that apply.
    - Attention: They pay more attention to things they can do to improve their

osteoarthritis symptoms
- Motivation: They are more motivated to take care of their health
- Feelings: They feel less anxious
- Physiology: They experience reductions in inflammation

A: As we mentioned in the past two videos, mindsets can influence a person's behavior, well-being, and health through all of the ways mentioned - attention, motivation, feelings, and physiology. These are a few examples of the ways in which that can happen.

3. In the first module, we learned about some of the most common misconceptions about osteoarthritis, and more encouraging ways to view it instead. How do you think that this relates to your mindset about osteoarthritis?

4. What challenges do you face with osteoarthritis? What mindset do you think would be helpful in overcoming these challenges and why?

Part 2: Mindsets and Exercise

VIDEO: Rethinking Osteoarthritis: Module 4 | Part 2

Reflection Questions

1. Take a minute to reflect on the mindset you currently hold about exercise. What images, thoughts, and beliefs come to mind? Has this changed over your lifetime?

2. In these videos, you learned that your mindsets can be self-fulfilling. This means that your mindset about the process of exercising can influence your health.
   - In light of this fact, how do you think your exercise mindset might be influencing your health and wellbeing?
3. Knowing all that you do about the power of exercise mindsets, what mindset do you want to adopt about physical activity and why? How does this compare to the mindset you currently hold that you just described?

Part 3: Adopting a More Useful Mindset

VIDEO: Rethinking Osteoarthritis: Module 4 | Part 3

Reflection Questions
You have just learned a lot about the power of mindsets generally, and about osteoarthritis and exercise mindsets in particular. In this video, we shared the following steps as a strategy for choosing your mindset:

1. Recognize your mindset
2. Remember other mindsets exist
3. Commit to one small step

When you start to notice your mindset, what is one question you can ask yourself to help consider how your mindset may be affecting and what mindset you may want to choose?

There are many ways to cultivate an adaptive mindset about osteoarthritis, including:
- Remember all the times you have successfully managed a challenge in the past or your body surprised you with what it is capable of doing
- Focus on the many strategies available to help you improve your symptoms and your health
- Talk to a loved one or a support group about what you have learned and how they can help support you in adopting a more helpful mindset

What are some ways you can practice adopting the osteoarthritis mindset you want to hold?

Ideas for cultivating an adaptive mindset about exercise may include:
- Focus on the social nature of exercise by going for a walk with a friend or loved one
- Remember the many ways in which exercise is improving your mind and body, such as boosting your mood and creativity
- Think of a way you can move that is fun, like playing charades or dancing to your favorite song
- Try a new way to move, join a new class, or move in a new location

What are some ways you can practice adopting the exercise mindset you want to hold?

Obstacles and challenges in life are inevitable and may challenge your mindset. What challenges might you face in adopting the mindsets you want? What are three things you can do to support yourself in cultivating an empowering mindset, even while facing a challenge?

Closing

In this module, we learned:
- What mindsets are, how they work, and why they matter

- The impact of mindsets in the context of osteoarthritis and exercise
- Strategies for choosing empowering mindsets
    - Recognize your mindset
    - Remember other mindsets exist
    - Commit to one small step

The mindsets we hold are not right or wrong, but they do have an impact. We hope that this module helps you bring awareness to the mindsets you hold and how they might be impacting your experience with osteoarthritis and exercise. Remember that you have the power to cultivate the mindsets that are most helpful to you and your health and well-being!

Module 4 References

1. Boles DZ, DeSousa M, Turnwald BP, et al. Can Being Healthy Be Fun and Indulgent Instead of Boring and Depriving? The Role of Mindsets in Motivating Healthy Behaviors.; 2020.
2. Crum AJ. Mindsets and health. (Under review).
3. Crum AJ, Langer EJ. Mindset matters: Exercise and the placebo effect. Psychol Sci 2007;18:165–71.
4. Elaine A. Rose and Gaynor Parfitt, “Pleasant for Some and Unpleasant for Others: A Protocol Analysis of the Cognitive Factors That Influence Affective Responses to Exercise.” International Journal of Behavioral Nutrition and Physical Activity 7 (2010): 1–15.
5. Zahrt, O. H., & Crum, A. J. (2017). Perceived Physical Activity and Mortality: Evidence from Three Nationally Representative U.S. Samples. Health Psychology.
6. Zahrt OH, Evans, K., Landay, J., Baiocchi, M., Murnane, L., & Crum, A. J. (in prep). Leveraging Wearable Fitness Trackers to Foster Adaptive Mindsets, Behavior Change, and Health Improvements.

Program Closing

Congratulations! You have completed the **Rethinking Osteoarthritis** program!

Throughout the program, we offered insights into what it means to have osteoarthritis, exercise with osteoarthritis, and use the power of your mind and body. You met world-leading experts in orthopedic surgery, osteoarthritis, physical activity, and psychology at Stanford University, including:

- **Bioengineering researcher**, Melissa Boswell, PhD
- **Orthopedic surgeon**, Nick Giori, MD, PhD
- **Bioengineering professor and researcher**, Scott Delp, PhD
- **Psychologist and professor**, Alia Crum, PhD
- **Health Researcher and Coach**, Kris Evans

We started by breaking down the common misconceptions about osteoarthritis and rebuilding more manageable and empowering views. We explored how physical activity can be enhancing and enjoyable. Finally, we shared how your mindset plays an influential role in your decisions to manage your osteoarthritis and your health.

You now have tangible and meaningful ways to cultivate a mindset that supports you!

Feel free to revisit the content in this program at any time and discuss it with friends and family or watch the videos again with them!

We hope that **Rethinking Osteoarthritis** has, and will continue to, positively impact your experience with osteoarthritis and your life.

Supplementary Note 7. Educational Intervention Content*

***Note: Associated videos are available upon request.**

Program Introduction

Welcome to **Understanding Osteoarthritis**!

This program will offer insights into what osteoarthritis is, clinical perspectives, and treatment strategies. The videos shared in this program were developed by world-leading orthopedic surgeons, physical therapists, university researchers, and arthritis organizations.

This program has four modules, each with a series of short films and reflective activities. Each module will take approximately 20-60 minutes to complete. You have one week to complete all modules of the program at your own pace. Our suggestion is to complete one module per day, but we encourage you to go at the pace that works best for you – whether that's faster or slower!

We hope that Understanding Osteoarthritis will help you improve your knowledge about osteoarthritis and learn strategies for osteoarthritis management.

Continue to begin Module 1!

Module 1: Understanding Osteoarthritis

Introduction

Welcome to Module 1 of **Understanding Osteoarthritis**!

In this module, we share a video created by Dr. David Halsey, an orthopedic surgeon at the University of Vermont Medical Center. Dr. Halsey talks about how to care for an arthritic knee or hip.

This module consists of four short films, followed by reflection questions on what you've just learned.

Part 1: Defining Osteoarthritis

VIDEO: Understanding Osteoarthritis: Module 1 | Part 1

Reflective Questions

1. What is the name of the tissue at the ends of the bones in joints?
   - Muscle
   - Tendon
   - Cartilage
   - Ligaments

   A: Cartilage is the tissue at the ends of the bones in joints.

2. What might someone with arthritis experience? Please select all that apply.
   - Pain
   - Avoidance of motion
   - Increased muscle tightness
   - Loss of motion

   A: Someone with arthritis might experience any of the following:
   - Pain
   - Avoidance of motion
   - Increased muscle tightness
   - Loss of motion

3. The articular cartilage is the only structure that supports shock absorption in the knee (T/F).

T: Not exactly! The synovial capsule, synovial fluid, and the articular cartilage all work together to provide the shock absorption that we need to carry out activities of daily living and recreation.

F: That's right! The synovial capsule, synovial fluid, and the articular cartilage all work together to provide the shock absorption that we need to carry out activities of daily living and recreation.

Part 2: Diagnosing Osteoarthritis

VIDEO: Understanding Osteoarthritis: Module 1 | Part 2

Reflective Questions

1. Which of these can contribute to osteoarthritis? Please select all that apply.
    - ○ Loss of the balance between cartilage breakdown
    - ○ Increase in cartilage components
    - ○ Loss of sponge effect of cartilage
    - ○ Decreased stress on the entire joint

    All of the following can contribute to osteoarthritis:
    - ○ Loss of the balance between cartilage breakdown
    - ○ Loss in cartilage components
    - ○ Loss of sponge effect of cartilage
    - ○ Increase stress on the entire joint

2. What types of things might a doctor do when evaluating knee pain?

    Great! A clinician evaluating knee pain may do the following
    - Take a detailed history
    - Perform a physical exam
    - Take X-rays
    - Perform blood tests to rule out other diseases

Part 3: Pain Relief

VIDEO: Understanding Osteoarthritis: Module 1 | Part 3

Reflective Questions

1. There are pills that cure osteoarthritis. (T/F)

   T: Not quite! There is no pill that can cure osteoarthritis, but there are things you can do to improve pain and function.
   F: That's correct! There is no pill that can cure osteoarthritis, but there are things you can do to improve pain and function.

2. What are two main treatment goals for osteoarthritis?

   A: Two important treatment goals are pain relief and return of function.

3. What are some treatment that were shared in this video?

   A: Nice! Some treatment strategies Dr. Halsey listed included over the counter medications, activity modification, ice, heat, exercise programs, and weight loss.

Part 4: Clinical Guidelines

VIDEO: Understanding Osteoarthritis: Module 1 | Part 4

Reflective Questions

1. Which of the following are strong recommendations by the AAOS Clinical Practice Guidelines:
   a. Strengthening and low impact aerobic exercise
   b. NSAIDs
   c. Glucosamine or chondroitin
   d. Hyaluronic acid injections
   e. Self-management programs

   A: Strengthening, low impact aerobic exercise, self-management program and NSAIDS are strong clinical practice recommendations.

2. What are two main treatment goals for osteoarthritis?

   A: Two important treatment goals are pain relief and return of function.

3. What are some treatments that were shared in this video?

A: Nice! Some treatment strategies Dr. Halsey listed included over-the-counter medications, activity modification, ice, heat, exercise programs, and weight loss.

Closing

In this module, we learned about:

1. Contributors to osteoarthritis
2. Treatments goals for osteoarthritis
3. AAOS Clinical Practice Guidelines

While there is no pill to cure osteoarthritis, it can be helpful to learn more about treatment goals and clinical recommendations for improving osteoarthritis symptoms.

Module 1 References

1. "What is Osteoarthritis?" YouTube, uploaded by The University of Vermont Medical Center, 24 May 2016, https://www.youtube.com/watch?v=8RMJOIZh75g.

Module 2: Osteoarthritis Pathophysiology

Introduction

Welcome to Module 2 of **Understanding Osteoarthritis**! So far, we learned about diagnosing osteoarthritis and general guidelines for treatment. In this module, we'll dive deeper into the pathophysiology of osteoarthritis.

These videos were created by Armando Hasudungan, a physician specializing in sharing biology and medical education. This module consists of three short films, followed by reflection questions on what you've just learned.

Part 1: Anatomy and Physiology

VIDEO: Understanding Osteoarthritis: Module 2 | Part 1

Reflective Questions

1. Cartilage contains pain receptors (T/F).

   T: Not quite! The periosteum is the sensitive material that wraps around the bone and contains pain receptors.
   F: You got it! The periosteum is the sensitive material that wraps around the bone and contains pain receptors.

2. What is the purpose of blood vessels? Select all that apply.
   - Bring in immune cells
   - Bring in nutrition
   - Draining waste

   A: Great! The blood vessels help with all of those activities.

Part 2: Clinical Presentation

VIDEO: Understanding Osteoarthritis: Module 2 | Part 2

Reflective Questions

1. Name one or more clinical presentations of osteoarthritis

   A: Good work! Clinical presentations of osteoarthritis include pain, stiffness, muscle wasting, tenderness on joint palpation, crepitations, and joint effusion, and reduced joint movement.

2. With age, cartilage tends to get thinner because it has decreased hydration. (T/F)

   T: Correct! Decreased hydration of cartilage is common with aging and can be one of the reasons of thinning cartilage.
   F: Not exactly. Decreased hydration of cartilage is common with aging and can be one of the reasons of thinning cartilage.

3. Everyone with osteoarthritis has severe pain. T/F

   T: It's surprising, but that's actually not true. Pain is complex and the amount of pain one experiences is related to many things, including how long one has experienced pain.
   F: That's it! Pain is complex and the amount of pain one experiences is related to many things, including how long one has experienced pain.

Part 3: Osteoarthritis Pathology

VIDEO: Understanding Osteoarthritis: Module 2 | Part 3

Reflective Questions

1. Which of the following are indications of osteoarthritis?
   a. Loss of joint space
   b. Osteophytes
   c. Subchondral sclerosis
   d. Subchondral cysts

   A: All of these are indiciations of osteoarthritis.

Closing

In this module, we learned about:

- The anatomy and physiology of the knee joint and how it is related to osteoarthritis
- Clinical presentations of osteoarthritis
- The complications of pain

We hope this module helped you learn more about the complexities of the body and how it relates to your experience with osteoarthritis.

Module 2 References

1. "Osteoarthritis Overview (causes, pathophysiology, investigations, treatment)" YouTube, uploaded by Armando Hasudungan, 30 Nov 2016, https://www.youtube.com/watch?v=pnKaBMvVUs0.

Module 3: Clinical Perspectives

Introduction

Welcome to Module 3 of **Understanding Osteoarthritis**! This module consists of videos created by Dr. Jesse Chrastil, an orthopedic surgeon from Panorama Orthopedics & Spine Center, and Ashley Ridout, a healthcare practitioner with the Precision Wellbeing Group. These clinicians go into further detail on the causes of cartilage breakdown and the available options for treating osteoarthritis.

This module consists of three short films, followed by reflection questions on what you've just learned.

Part 1: Arthritis Types and Options

VIDEO: Understanding Osteoarthritis: Module 3 | Part 1

Reflective Questions

1. Osteoarthritis is the only type of arthritis (T/F)

   T: Not exactly. Other types of arthritis include post-traumatic arthritis, gouty arthritis, and autoimmune arthritis.
   F: That's right! Other types of arthritis include post-traumatic arthritis, gouty arthritis, and autoimmune arthritis.

2. What are the characteristics of osteoarthritis that are visible on an X-ray?

   A: Clinicians typically diagnose arthritis when they see characteristics such as joint space narrowing and bone spurs on an X-ray.

Part 2: Causes of Cartilage Breakdown

VIDEO: Understanding Osteoarthritis: Module 3 | Part 2

Reflective Questions

1. Osteoarthritis is only due to using the joint too much (T/F)

   T: Not quite. Joint mechanics, family history, trauma, and body weight can also play a role in osteoarthritis development.
   F: You got it! Joint mechanics, family history, trauma, and body weight can also play a role in osteoarthritis development.

2. How can one's occupation or involvement in sports affect their risk of osteoarthritis?
   A: Nice! Occupations or sports that involve a lot of heavy lifting or repetitive high-intensity pounding or vibrations can increase the risk of developing osteoarthritis.

Part 3: Patient-Centered Management

VIDEO: Understanding Osteoarthritis: Module 3 | Part 3

Reflective Questions

1. X-rays are the only indicator of osteoarthritis (T/F)

   T: That's not exactly right. An individual's age, sport and occupation history, and symptoms such as knee stiffness or grinding can indicate osteoarthritis.
   F: You got it! An individual's age, sport and occupation history, and symptoms such as knee stiffness or grinding can indicate osteoarthritis.

2. What is one patient-centered osteoarthritis management approach?

   A: That's it! Patient-centered management approaches include:
   - Maintain joint strength and stability
   - Prevent the joint from being overloaded
   - Pain relief from things like ice and movement
   - Modifying daily activities

Closing

In this module, we learned:

- There are different types of arthritis
- There are many causes for osteoarthritis beyond just joint overloading
- X-rays are not the only way to diagnose osteoarthritis
- Ideas for patient-centered management

There are common misunderstandings about osteoarthritis. We hope this module helped you learn more about how osteoarthritis develops and is diagnosed and ideas for management.

Module 3 References

1. "What is osteoarthritis?" YouTube, uploaded by Panorama Orthopedics & Spine Center, 15 Nov 2020, https://www.youtube.com/watch?v=FIBizZ1uBVg.
2. "What is ARTHRITIS / OSTEOARTHRITIS?" YouTube, uploaded by Precision Wellbeing, 3 Jul 2021, https://www.youtube.com/watch?v=yPcPIa9rYk4.

Module 4: Treatment and Patient Perspectives

Introduction

Welcome to the final module in **Understanding Osteoarthritis**! In Module 4, we will go into more detail about treatment strategies for osteoarthritis and hear from patients about their experiences. In the first video, Dr. Dana DiRenzo from the Johns Hopkins School of Medicine discusses treatment strategies. In the second video, Advanced Practice Physiotherapist Suzanne Denis provides information on how exercise and physical activity can help osteoarthritis. In the final video, firefighter Kelly Barber tells his story about having osteoarthritis to help people understand that arthritis affects healthy, strong, and active people and that more research is needed to prevent this debilitating disease.

This module consists of three short films, followed by reflection questions on what you've just learned.

Part 1: Understanding Treatment Strategies

VIDEO: Understanding Osteoarthritis: Module 4 | Part 1

Reflective Questions

1. What are the two main types of management for osteoarthritis?

   A: The two main types of management for osteoarthritis are medication and non-medication management.

2. What is an example of medication management recommended for osteoarthritis?

   A: Pain medication and topical gels or creams are examples of medication management.

3. What is an example of a non-medication management tool recommended for individuals with osteoarthritis?

   A: Exercise, weight loss, tai chi, acupuncture, and yoga are all examples of non-medication management.

Part 2: Osteoarthritis and Exercise

VIDEO: Understanding Osteoarthritis: Module 4 | Part 2

Reflective Questions

1. 1. When does stiffness tend to occur in people with osteoarthritis?

   A: Stiffness most commonly occurs in the morning and after being in one position for too long.

2. What are some consequences of becoming less active for individuals with osteoarthritis?

   Consequences of becoming less active can include:
   - Physical deconditioning
   - Loss of strength
   - Progression of osteoarthritis
   - Low mood
   - Fatigue

3. What are some of the benefits of exercise and activity?

   A: Keep it up! Exercise and activity are beneficial for osteoarthritis and generally, including:
   - Relieve stiffness
   - Improve strength and energy
   - Make everyday activities easier
   - Increase energy
   - Improve mental health
   - Decreased risk of other chronic conditions

4. Standing for long periods of time can be detrimental to one's cartilage. (T/F)

   T: That's correct! Standing for long periods of time can make cartilage more vulnerable. This is another reason why movement is important!
   F: Not exactly. Standing for long periods of time can make cartilage more vulnerable. This is another reason why movement is important!

Part 3: Patient Perspectives

VIDEO: Understanding Osteoarthritis: Module 4 | Part 3

Reflective Questions

1. When did Ken first notice that he had osteoarthritis?

   A: Ken noticed the pain when he started working in his office and couldn't get comfortable sitting at his desk. When he realized this pain was not "normal" compared to other people in his life, he got an X-ray and was diagnosed with hip osteoarthritis.

2. What do you think contributed to Ken's osteoarthritis development?

   A: Ken's high-impact lifestyle and profession as a firefighter may have contributed to his osteoarthritis.

3. How did Ken's daily habits change after he developed osteoarthritis?

   A: Ken reduced the number of high-impact activities. He noted that if he were diagnosed earlier, he would have moved to more low-impact activities sooner. He is also now an advocate of osteoarthritis research and shares his story to help others with osteoarthritis.

Closing

In this module, we learned about:

- Treatment strategies for managing osteoarthritis symptoms
- The benefits of exercise for osteoarthritis
- The experiences of a patient with osteoarthritis

Module 4 References

1. "Understanding Treatment Options for Osteoarthritis (OA) | Johns Hopkins Rheumatology" YouTube, uploaded by Johns Hopkins Rheumatology, 8 Apr 2020, https://www.youtube.com/watch?v=1pN6IvUjaaM.
2. "Introduction to osteoarthritis and exercise" YouTube, uploaded by Arthritis Society Canada, 24 Mar 2020, https://www.youtube.com/watch?v=FtQF0iZixmY.
3. "Series 2 Part 1: Intro to exercise for osteoarthritis of the hip and knee" YouTube, uploaded by Arthritis Society Canada, 5 Feb 2021, https://www.youtube.com/watch?v=qPW6_dMe-2I.

4. "Osteoarthritis in his 40's: Firefighter Kelly Barber Tells His Story" YouTube, uploaded by Arthritis Research Canada, 20 Dec 2012, https://www.youtube.com/watch?v=EgCDdBWma1Q.

Program Closing

Congratulations! You have completed the **Understanding Osteoarthritis** program!

This program offered insights into what osteoarthritis is, clinical perspectives, and treatment strategies. The videos shared in this program were developed by world-leading orthopedic surgeons, physical therapists, university researchers, and arthritis organizations.

We hope that **Understanding Osteoarthritis** helped you improve your knowledge about osteoarthritis and learn strategies for osteoarthritis management.

Supplementary Results and Data

Supplementary Table 8. Baseline descriptive statistics for participants who completed the study or withdrew or did not complete the study.

Characteristic	Completed N = 408	Withdrew N = 50
Age, mean (SD), years	63.6 (8.8)	62.5 (9.2)
Sex, n (%)		
Male	163 (40.0)	19 (38.0)
Female	244 (59.8)	31 (62.0)
Nonbinary	1 (0.2)	0 (0.0)
Not listed	0 (0.0)	1 (2.0)
Gender, n (%)		
Male	163 (40.0)	19 (38.0)
Female	243 (59.6)	31 (62.0)
Transgender Male	1 (0.2)	0 (0.0)
Transgender Female	1 (0.2)	0 (0.0)
Gender variant/non-conforming	0 (0.0)	0 (0.0)
Not listed	0 (0.0)	0 (0.0)
BMI, mean (SD), kg/m2	34.2 (7.9)	34.2 (8.7)
Race and Ethnicity (not mutually exclusive), n (%)		
American Indian or Alaskan Native	9 (2.2)	0 (0.0)
Asian	16 (3.9)	4 (8.0)
Black or African-American	19 (4.7)	2 (4.0)
Hispanic or Latino	14 (3.4)	6 (12.0)
Native Hawaiian or Pacific Islander	0 (0.0)	0 (0.0)
White	364 (89.2)	39 (78.0)
Other	8 (2.0)	1 (2.0)
Currently employed, n (%)	171 (41.9)	20 (40.0)
Comorbid conditions, n (%)		
Heart disease	35 (8.6)	2 (4.0)

High blood pressure	205 (50.2)	23 (46.0)
Lung disease	14 (3.4)	3 (6.0)
Diabetes	86 (21.2)	10 (20.0)
Ulcer or stomach disease	5 (1.2)	3 (6.0)
Kidney disease	25 (6.1)	1 (2.0)
Liver disease	8 (2.0)	2 (4.0)
Anemia or blood disease	10 (2.5)	0 (0.0)
Cancer	16 (3.9)	3 (6.0)
Depression	78 (19.1)	7 (14.0)
Back pain	150 (36.8)	12 (24.0)
Unilateral symptoms, n (%)	137 (33.6)	14 (28.0)
Time since pain started, mean (SD), years	9.7 (8.4)	11.7 (11.3)
Average pain in most painful knee, mean (SD), 0-10	5.3 (2.0)	5.1 (1.8)

Abbreviations: BMI, body mass index (calculated as weight in kilograms divided by height in meters squared)

Supplementary Table 9. Three of the open-ended responses to *Rethinking Osteoarthritis* at the end of the program.

Rethinking Osteoarthritis		
**What, if anything, did you find exciting or helpful about this program as a whole?**	**What, if anything, did you find confusing or unhelpful about this program as a whole?**	**Do you have any additional comments about or suggestions for this program?**
The program really made me think about arthritis and its overall affect on me and what I can do to help myself. I really feel hopeful.	Nothing	I'd love if if this were accessible in the future as a "refresher". I really feel motivated and don't want to lose that.
It did not address diet as being as an important part.	nothing	none
The idea that exercise actually helps to manage and even improve mobility for those of us with osteoarthritis. Hearing from others with osteoarthritis was helpful.	I still do not have a full understanding of what osteoarthritis is and how it progresses.	I'd have benefitted from more information on the anatomy and physiology of arthritis. More examples and stories from real life. I wish there were a local group I could join to help keep my new motivation going !
I think it helped me appreciate how important it is to attack this problem, bc mindset keeps me stuck.	Sometimes I think blaming negative mindset is like blaming a victim. Also a way of deflecting the poor medical care in this country.	
	No discussion of pain associated with osteoarthritis and non medication ways to deal with it	
The detail information regarding both mindsets and osteoarthritis. Nothing	Nothing	No
I really liked the educational aspects. When I got my diagnosis, I didn't get any info about OA so I'm really glad I got	nothing was confusing or unhelpful.	I really liked this and am glad I got to see it.

to see these videos. Especially: learning about strengthening the muscles and tendons around the joint, that pain in and of itself is not evidence of more damage.		
It was interesting, made me think	A lot at one time	Extend module completion, maybe only 1 a week
Short, easily digestible amounts of information	Videos in final module were noticeably longer than the previous sections, which was a bit of a letdown	
It gave me prompts to make my activity more exciting. The realization that my mind is an important part of my exercise program and success.	Some of the the 'testimonials' didn't necessarily connect with me.	I'd love if my mother with Parkinson's would take part in a program like this. (She probably wouldn't on my suggestion however)
not much.	I already do a lot of exercise in general and targeted to overcome some weak spots. I still fail to see anything positive with arthritis.	better screen for non-athletes
the positive take on things	nothing confusing	
It gave me hope. All positive reinforcement. Things are not so bleak as I thought.	Nothing.	No.
Sometimes I need to be reminded of things I already know. Plus I really did think things were bound to just continue downhill, happy to hear that's not necessarily the case	nothing	no
new information that I was never given when I was diagnosed, positive focus	nothing was confusing but I think some more tangible examples	no
I found it to be motivating.	This was very easy to understand.	Not at this time.
That the benefits of exercising can be enjoyable and obtained	nothing	It appears to be very motivational for people to get up, get out, and

without rigorous exercises which include repetitions and sweat.		get moving in enjoyable ways to improve your arthritis and your attitude.
It made me rethink my attitude toward exercising, telling myself that I can do it.	Nothing	I found the constant hand gesturing by the speaker distracting, and extremely annoying. Also, the music is annoying as well.
It's a good reminder for a positive approach to arthritis	Nothing, it was very clear	find more varied people to interview for your patient video shorts. The subjects were boring and clearly reading a script.
A very manageable and realistic approach	Nothing	It‚Äôs very relatable. A Just try something kind of approach
i liked the focus on the positive; that this is a disease that can be managed	too many modules and far too long..........also pretty basic for those of us who have any experience with alternative therapies	i think one thing missing is the relationship with the care provider.....in fact maybe they should be included in the audience....the care and information i have received was very poor - basically a shot of cortizone and move on down the line........i think providers need to know this
I have found this program to be very positive! It was encouraging and I found it very helpful in my thinking about exploring new ideas for exercising!	Nothing was confusing	I have enjoyed all of the modules! The mindset idea really resonates with me and my outlook on this diagnosis.
That I can‚Äôt think of it as damaged	None of it	Nope
That being diagnosed with osteoarthritis is not a sentence to inactivity and pain for life.	Nothing	Anyone diagnosed with osteoarthritis should watch these videos.
Movement will not damage the joint further.	Changing mindsets. Or that we all possess a mindset or multiple mindsets.	Presenters seemed to know their subjects well. Examples of beginning exercise programs might be helpful.
I found the idea of "osteoarthritis as manageable"	I was confused about the idea that exercise couldn't hurt your	This program would be most beneficial to patients/clients who

to be helpful. I also found the concept of thinking differently about exercise to be extremely helpful. I am excited to find fun ways to move more and for movement to be more enjoyable.	joints. Depending on your level of osteoarthritis, some kinds of exercise can damage your joints further. This is factual and should be clarified. Maybe it was clarified, and I missed it.	are newly diagnosed with osteoarthritis of the knee(s), who are in the earlier stages of osteoarthritis. That said, I am in later stages of osteoarthritis in both knees, but still found this program helpful because I filtered out somethings that I felt applied more to people in the earlier stages of osteoarthritis, such as what I pointed out above.
Changing my mindset is important and exercise helps to lubricator joints and reduce inflammation.	I found nothing to be confusing or unhelpful.	I really enjoyed all of the modules and plan to make changes to improve my OA.
Broken up into segments and thought provoking questions and suggestions	Videos and modules were too long, then having to answer all the questions after very time consuming	See above notes and thus was very time consuming, especially the small amount of money offered ($10)
Learning about the joint actually being bathed in synovial fluid and that there are ways to build strength and reduce pain	Nothing	Suggestions for what to do at night when the pain makes one feel like one is never going to sleep and never going to get better.
I'm glad it used a different approach than just prescribing drugs.	I already knew the information and already had the mindset that OA is manageable. Some forms of exercise are fun, but things like PT exercises are boring . It seemed like you said the same thing over and over.	I find the background music on the video distracting and it makes it hard to hear the speaker.
The whole mindset review	I had already adopted some positive attitudes about my osteoarthritis. The survey does not reflect an option to answer accurately	Quite helpful. I hope I can share this with friends and family who are not dealing well with their condition
Overall the amount of time it takes is too high	Don,Äôt know	Reduce the time of these
reinforcement of behavior changes to continue mobility	mindsets not as helpful, but either was mindfulness for pain	enjoyable.
The info on mindsets	Nothing really	No

That exercise should improve my osteoarthritis.	Nothing	No
Everything!	Nothing.	No. It was very well done.
The encouragement to adapt one's mindset and to not view osteoarthritis as limiting.	Nothing necessarily.	None that I can think of. Thought it was well constructed and helpful.
Putting feelings into words.	losing my place when interrupted	not really
It really made me think about things in a different way.	It was just a bit long.	No not really.
The mindset is the most useful as a technique and can be used in all aspects of life	Nothing but I,Äôm pretty educated on the topic	No
learning about the meaning of mindsets	I found nothing confusing.	none at this time
The brain is powerful and we need to keep our belief systems in check.	Nothing	This was a good reminder that nothing stays the same forever. We have the power to change the way we think.
That osteoarthritis progression can be mitigated and perhaps stalled.	The concept that pain was primarily due to the expectation of pain.	I feel the messaging is not consistent. The stated concepts say, "it's manageable with mind magic", yet - to me - the unmistakeable take away from your interviewees was that "it's about learning your new limitations and living within them". I have a hard time reconciling those two. Many/most of the coping mechanisms you tout I have been using for years, so much of that is a bit of restating the obvious to me.
exercise can be helpful	nothing	Thank you
The discussion of mindset was useful as it is something I always struggle with.	I would have appreciated more technical discussion on the physiology around	Provide resources on massage and other physical treatments that can help with the after

	Osteoarthritis to debulk the myths there.	exercise recovery specific to osteoarthritis. Often this recovery can be a barrier to staying active the next day.
very complete presentatioin	nothing	glad I joined
I learned a lot about arthritis that I didn‚Äôt know. I have to change my mindset if I am ever going to start exercising regularly.	I didn‚Äôt really find anything confusing or unhelpful.	No
OA very manageable	Nothing	Great stuff
Exercise is beneficial in managing my arthritis.	None	None
It is just a reminder to not let OA hold you back but that you may have to find new activities	No	Some of the people felt very scripted and not as genuine and therefore it came across a little condescending.
Mindsets		More information about pain management
The fact that I have many of the misconceptions that were outlined in the first part and now I know that most of them are not correct	The whole concept of mindsets is a little confusing, but I believe that I understand for the most part.	I felt that the participation in the survey was very helpful. The length of the survey kept me interested throughout. I have done other surveys in the past on other topics and they get so drawn out that you lose interest halfway through it.
The concept of shorter, more frequent exercise sessions	Nothing confusing. Some of the testimonials were a bit redundant.	On the whole the medical community in my experience leaves a lot to be desired insofar as their ability to put forth a positive mindset to those suffering with OA. HOWEVER,you folks pick ed up the ball nicely and I'll be forever grateful

The $10	All the happy-clappy nonsense	
The new information about my condition and the way in which I could easily understand the information.	Nothing.	I wish there were some specific resources or exercise regimens and programs suggested or recommended.
It,Äôs helpful for people who may not have in depth knowledge of osteoarthritis and the benefits and importance of exercising	N/A	N/A
New perspective	Nothing	Thought it was good
I liked all of the information, even though I'd heard most of it before. But I liked how it was short, to the point, and included lots of good information with examples.	The only thing I didn't like was how they'd tell us information, like 'even though it hurts when you exercise, it isn't wearing down your knee more', but not provide any evidence or studying supporting this. I'd like some more information..so why does it hurt more when I exercise if it isn't damaging the cartilage more, etc.	Like I said above, I'd like some more information about what is really happening in a knee with osteoarthritis. Why does it hurt if it isn't 'bone on bone,' how does movement repair it, how come it only hurts when I exercise? Etc.
The voices of the speakers are C A L M ing and gentle. I never heard the words you MUST do this. I heard a lot of TRY... maybe its not a today thing but TRY..... and from my doc is persistence. Do not give up.	Nothing at all, 100% safe	I really liked this and I love you can stop and start. So thank yoU!
Rethinking mindset	Nothing to negatively comment it was all positive and excellent information	Share it with others
That Osteo arthritis is not a curse	Not applicable	Not at this time
I can refocus!	Nothing	
The positive encouragement.		Very minor point: Reduce the hand gestures by half.

Gave me a new perspective on osteoarthritis as manageable. It knocked down sone misconceptions I had.	N/a	I really enjoyed this experience and hope to make changes!
All was exciting	None	No
Helpful strategies to manage OA	Concerned around some of the mindset messaging. Not all physical issues can be changed with a positive mindset. The module may cause harm in making a person have feelings of failure or guilt if physical issues are not improved with a positive mindset.	Add content that acknowledges that you don‚Äôt need to feel guilty or like a failure if your symptoms don‚Äôt change with this program
The reminder that I have the power to change my mindset. And that it is a privilege to have the time and resources to focus on myself and my health.	Somewhat confusing: How to integrate surgery (in my case the prospect of two knee replacements) into what I learned. I am wondering if rethinking exercise (shorter bouts every day, focusing away from pain) will be as helpful ultimately as surgery.	I have been diagnosed with very advanced bone on bone arthritis in both knees. But wonder if this means surgery is ineveitable. Experts spoke about delaying the progress of the disease. Can it get worse from here. Can "advanced" arthritis be manageable in the same way as "less severe" arthritis?
nothing exciting	nothing	
Very informational and motivating to change my mindset about the osteoarthritis	not at all, i think the information is very clear	great. i don't think i would change anything.
Well, for me, it‚Äôs all new and enlightening information. I just started having problem with my left knee and I‚Äôve been spending a lot of time not doing things because of the pain and I realize that that may have made my situation worse. So, now I have better information on how to deal with osteoarthritis.	I didn‚Äôt find anything confusing or unhelpful.	Thank you for preparing such a simple yet affective set of videos to educate people with osteoarthritis about the condition they have and what they can do about it.
Osteoarthritis is manageable	no	no

New ideas about how to think about exercise and mindset	None	Thank you
The mindset that osteoarthritis is manageable.	Nothing I can think of.	Cost of group activities can be a barrier for some. I don't think this was factored in.
Recognizing tha small amounts of mild to medium exercise multiple times a day is just as important as strenuous exercise.	Nothing	None
The motivational tone of the presenters was inspiring	Lack of acknowledging genetics and co- morbiditiies are influences.	thank you for sharing this program
general information was helpful	nothing	no
The factual part	Sometimes the speakers don't blink, sometimes they seem like they are reading a teleprompter	No
It helped me feel powerful to improve my situation instead of fearful and limited without major medical intervention	Nothing	No
As a whole, I realized that physical activity / exercise is still very beneficial to me even with arthritis.	Nothing.	Thank you! I found this to be extremely motivational, helping me realize that not only could I but I needed to get back to working out consistently despite, and even because of, my arthritic knees!
That exercise can make changes such as increased synovial fluid, less inflammation	Because I have seen such worse conditions in other people I probably didn’t have much of a mindset.	
This week's module or the entire program to date?	The most helpful so far was the rethinking of exercise as something to avoid and the least.helpful to me.wws.this module (mindset)	No
Upbeat. Motivating.	Way too long. Could have been condensed into three videos total.	

Lots of good things I can talk to, even with parents, who suffer from this type of pain.	Nothing	Nope
Gave a different petspective	It was very clear on action items to help accomplish goals.	The program was helpful as is
Validation of daily activities as exercise	Representation matters. People with PCOS and other conditions causing obesity exist and have osteoarthritis. It would have been nice to see a couple in the interviews, stills, or videos	Remember, we are not all grey haired and thin.
That more shorter walks are better than one long walk	The mindset section	
Mindsets	Nothing	No
The timing and relevance of this program was perfect for me. It gave me the mindset that I needed.	nothing	Excellent program. Very understandable and enjoyable. Thank you for including me.
Motivation!	It was nice! No confusion.	It was quite motivational.
It made things easy to understand. Having lay people give their views also was helpful.	Nothing.	Very well done. Thanks for allowing me to participate.
New ways of thinking, understanding osteoarthritis, and understanding yourself.	Nothing.	Not at this time.
Information about bone on bone not being a death sentence.	All good	No
I am excited about developing a positive mindset when it comes to moving forward with osteoarthritis.	I didn't find any part of this program confusing or unhelpful. In fact, the opposite was true.	I wish there was a way to incorporate the mindset you have cultivated in this program with more healthcare professionals. Many approach osteoarthritis with a totally different mindset.

Doing something everyday no matter how small	nothing. It was very straightforward which I appreciated	no
Mind set keep moving	None	No
Good integration of physical and mental aspects	Testimonials are only marginally beneficial	Could dig deeper into addressing barriers or obstacles in changing mindsets
There is life with this	Mindset means a lot	Not right now
looking at things differently	nothing really	no
The possibility of still being active and keeping the knees lubricated and strengthening muscles	Nothing	Not at this time
I found it encourageing and motivational		I liked the way that, rather than just imparting information, it made me think about everything and relate it to what I can/need to do now, going forward, to improve my life with osteoporosis.
Presentation	Nothing	Informative
The knowledge that exercising won,Äôt cause more harm to the joint.	N/A	
Good reminders that mild exercise does count, and that I in fact enjoy much of it. Try to brush the gym-oriented cobwebs from my consciousness.	I hate videos :-). This could have been better presented in writing, taken a lot less time and been just as helpful. Remember, much of your target audience is older, we are literate...	I'd give a 'transcript' option as an alternative to the videos. With that I think it'd be helpful to the general public. There's been way too much misinformation on arthritis from drug advertising, and pressure to get joints replaced without trying to improve matters. Also, you mention briefly the proven fact that pain is almost entirely disconnected from radiological findings, as well as ability to exercise, I think it'd be helpful to most people to really emphasize

		that, it deserves a section of its own.
Got me thinking that maybe I can have less pain	It was pretty clear	Nope
Just hearing that arthritis can be managed and slowed.	Melissa is a wonderful speaker but her (excessive) arm movements became a distraction for me. But the program was very helpful.	
that concepts and activites I've leaned 40 to 50 years ago are becoming 'mainstream'. These include tai chi, yoga, meditation and NLP	nothing, really. This is an excellent collection of ideas and presentations!	not at this time
All was informative	Nothing	No
The people were very relatable and easy to understand.	Nothing	No
Very informative.		
finding new ways to exercise	unsure	no
New ideas to think about.	None	I would like some exercise plans to try out to go along with this program.
That exercise is not going to make things worse and to do more.	Is it all geared to knees? What about my back is it helpful there also? What is the best exercise for the back?	I know this is a stand alone thing. Asking questions would be really nice.
The presentors way of explaining ideas. And the testimonies from actual people with osteoarthritis.	Nothing.	It was great!
The idea that changing my mindset will lead to greater success	It was well presented	
learned how much more valuable exercise is as it wasnt	nothing	none

really explained by PT, DR or Ortho		
The program provided information I did not previously have and motivates me to do exercise.	Nothing.	At times, the audio was not in sync with the movement of lips on the video.
It opened my world to an exciting future in dealing with osteoarthritis	Nothing. It was first class in all respects	Thank you for opening my eyes and improving the way I view osteoarthritis and the ways that I can manage it
Hearing from patients	N/A	No,very helpful
Its ok to feel pain.	didn't mention anything about taking pills to offset pain? Aleve Asprin ect....	None will need to try these new concepts.
Remind me about incorporating nature to increase enjoyment of what I need to do	nothing	no
Really reinforced the concept of how beneficial exercise is. I recently learned that exercise is medicine and this program reinforced that ifea	The length could be shortened a bit.	Overall really impressed and I learned a lot. Thank you.
Things I found exciting and helpful: that osteoarthritis is more manageable than I realized; that movement can increase lubrication; and that I'm on the right track with a positive mindset.	I didn't find anything confusing or unhelpful, although it was a fair amount of information to take in and I'll likely review some of it.	I think the video lengths and timing of information was pretty right on. The speakers are personable and generally engaging. The stock images are pretty good. The slides that recap info are helpful. I'd love to share this program with friends who have osteoarthritis in joints other than their knees, but maybe the info is more specific to knees. Great job overall and I really enjoyed this!
Positive emphasis	Nothing	Thank you for this program

Supplementary Table 10. Three of the open-ended responses to *Understanding Osteoarthritis* at the end of the program.

Understanding Osteoaritis		
**What, if anything, did you find exciting or helpful about this program as a whole?**	**What, if anything, did you find confusing or unhelpful about this program as a whole?**	**Do you have any additional comments about or suggestions for this program?**
The story at the end helped me to see what I may have done in the past that contributed to my oa	The second module man was too indepth for me to really focus	Lose the guy who gets so technical as it's above a normal persona intellect
Finding out my personal mindset of exercise and weight bearing is helpful and need to focus on weight loss.	I think it was very good, although a bit depressing to realize that overall there is no non surgical ‚Äòcure‚Äô	No
Continual suggestions of medical and non medical approaches.	The video with many medical terminology.	Informative, user friendly, easy to follow, encouraging, educational , and insightful
The anatomy module was interesting, along with spelling Pout the reasons why exercise is helpful.		
I like the end where you had the Fireman tell his story and it was related able to me and should be taught in school to avoid in future for the adult whom is likely to be diagnosed as oa.	Some days of repetition of ideas especially in the beginning. I didn‚Äôt like the drawing learning. There were overwhelming information there.	No thanks
I found the explanations of how to manage arthritis helpful.	I found the second series of videos way too technical for me. The person talked and drew too fast and there is now way I will remember all that terminology.	The third series of videos need editing. The first video is 16 min. and the other two are repeats of what was in the first one.
I found it intwresting that both exercise and weightloss can help with pain management.	I did not find the program either confusing or unhelpful.	No
I liked the detail about what happens physiologically in the knee and also the discussion about which treatments were	Some of the terms weren't explained well: crepitation, subchondral sclerosis, subchondral cysts	I would have expected the program to mention that before beginning an exercise or activity program it would be

proven effective and those that were not effective.		wise to consult a doctor and a physical therapist
I appreciated the varied perspectives provided by the different speakers. That kept my attention as well as provided new information to me. I also liked that the information was "chunked" into digestible bits. The knowledge checks also helped me retain the information.	In the 3rd series of videos, there was significant repetition, which was distracting.	I think it's designed to easily fit into one's schedule, making it more likely to be useful to people.
Very supportive and good explanation of the problems with osteoarthritis. Exercise was encouraged but not in a threatening manner	Some of the medical terminology and explanations in module 2 could be confusing to a lot of people.	Good information. Very helpful
Knowing I‚Äôm on the right track.	One video said glucosamine was not recommended, another said it was. There were also two videos that disagreed about the value of acupuncture.	The video with all the drawings (where the narrator persistently said ‚Äúvalrus‚Äù when he meant ‚Äúvarus‚Äù presented too much info too fast for anyone who have studied anatomy/physiology.
Information about the benefits of certain types of exercise.	The medical terms about the different parts of the knee and medical terms got a bit confusing.	Maybe make the technical medical stuff a little bit easier to understand with having a bunch of medical terms thrown at you quickly.
The emphasis on early detection and the importance of exercise	I felt it was good information and nothing was confusing	I will follow up on YouTube to view additional information mentioned in the footnotes because of my own personal situation with OA.
To a certain extent, I appreciated the early videos that explained, with graphics and photos, how arthritis presents and what causes it. That said, the video with all the hand drawing was way too technical (and I'm a person drawn to technical	Some of the presenters contradicted each other; for example one doctor said that arthroscopic surgery does not work for osteoarthritis, but your physical therapy doc said that debridement works. I also found this sequence of videos to be highly repetitive.	The exercise portion would have been more effective if it demonstrated simple, effective exercises to aid knee osteoarthritis instead of simply talking about why exercises matter.

explanations--this was too much.).		
real people sharing their experiencd		
Understanding how the knee joint works and how OA modifies the joint	Lots of medical professionals talk about benefits of PRP and hyaluronic acid to treat knee OA pain, but the video says there is no evidence it works	No
All of the speakers were encouraging. One was a little too scientific and one was a little too "bland"--unexciting.	The program as a whole was very contradictory, e.g. Glucosamine, scoping vs. non-surgical treatments.	Overall, very informative. I just did not like the discrepancy in treatment. Some videos seemed a little dated.
I think the first three videos with the male doctor were ok as a basic intro to OA.	Most of the videos were waaaay too long and boring. I would have rather just read a few slides that included the same info. The one with all the drawings and arrows and discussion of the anatomy was terrible and too much information I didn't need or want.	No.
The different options for treatments		
Information needed. Should have received more info from my doctors.	Nothing.	Great project
Better understanding of what ortho drs. Have told me. Why they won't give shots, Why exercise is imprtant. They all had the attitude that I should just do as they say and not question it. This made me feel like I could be more of a partner in any therapies given.	There were a couple of conflicting opinions between doctors and the physical therapist. They are minor. One was the effectives of glucosamine. The other on joint debriding. Also, the firefighter's name was Kelly, but the questions refered to Ken	Thank you for this opportunity to learn.
There physiology was repeated multiple times, but there was very little on concrete action plans. Other than mentioning yoga and ti chi once, there was no mention of specific	Loys of repetitive information, although some of it was conflicting, such as the gentleman who recommended ‚Äújoint clean out,‚Äù after a presentation that	Not really very helpful. I understand why I have OA, but only understand what to do in a very general way.

exercises that might be beneficial.	stated it has not been shown to be of benefit.	
Clear sense of what to expect in the future and how I can influence that future		
I received a lot of new information and discovered that some previous ideas about arthritis were not correct.	I had to concentrate hard to understand those with English accents.	It mde me more hopeful about controlling my symptoms.
reinforcement of things I already knew.	nothing.	i would like to have been shown actual exercises.
The understanding of osteoarthritis. Diagrams certainly helped. You reached all the different learning styles.	A lot of ‚Äúmedical‚Äù words I couldn‚Äôt understand , which made some info difficult to comprehend.	I‚Äôm happy I decided to look into it.
The right exercise is important	Way too much repetition	No
Need to continue to exercise	More understanding of OA	None
I found very little helpful. In fact it upsets me greatly that there is no cure. I have to give up my sports and pop pills the rest of my life. I‚Äôm depressed now.	I already knew all this stuff. In fact I think some of this is wrong. For example there is little peer reviewed research supporting some of the claims some of the speakers discussed.	I don‚Äôt see how this research or whatever it is- not sure you have a control group- is going to help anyone.
Encouraged me to think about getting more active	The one speaker was so monotone it was hard to stay engaged	No
Practical advice	Indepth scientific explanations	A lot of it was repetative so far
The treatments that are available	none	no
Review of all avenues to help relive arthritis pain and prevent further joint problems I‚Äôm glad I signed up	Weight loss as part of improving arthritis pain	Im glad I signed up. The information is motivating me to continue what I am doing and gradually increase my exercise program

Great information and general options for care	The science of cells might be too detailed for some	I appreciate being involved. This should be an option for ortho patients to view
list of medications, what is good and what is not recommended	Nothing confusing	no
I really appreciate the anatomy and description of osteoarthritis. My MD never explained anything nor made suggestions other than lose weight (obvious). I understand better now why he didn't have much to offer as a treatment plan.	A little repetitive with multiple explanations if the same info (but not too bad).	I'm glad I got to see the videos and I will work harder on preserving my remaining joints!!
activity is good, even if you have bone on bone	should I use glucosamine & chondroitin?	the focus on activity and exercise was encouraging.
I found it helpful to learn that I can increase my activity and it will not make my arthritis worse.	I found the long description of how and why arthritis occurs to be confusing because of the many medical terms used. Also, it was very fast paced.	I found it to be a very informative program.
information	nothing	no
Break down of osteoarthritis, cause and effect	Ken's story at the end I did not understand the purpose	not at this time
Physical abnormalities in joint structures can increase arthritis low impact exercise can improve synovial fluid flow j and out of joints. glucosamine chondriton has little proven benefits	Some area had redundancies, Orthotics are a must for my flat feet and resulting knee hip pain, yet insoles were said to not help	Other medications can increase inflammation and pain exacerbating arthritis for me heart and cancer drugs
I always enjoy learning more about my condition and non surgical ways to magage it	Na	I like the program so far just hoping at some point something comes out to help me specifically
Interesting		

Better understanding of methods for addressing the challenges of my knee issues	Nothing	No
That it can be manageable	Nothing	No
makes me think	meddical terminology	no
Realizing that you are responsible for management of your condition	Nutritional health was not complete. I think diet for people over 50 is paramount to good joint health	Would like to have seen specific exercise recommendations
importance of movement still	nothing	no
It was informative and gave science back answers.	Nothing was confusing or unhelpful	I think specific knee joint strengthening exercises would be good to have.
Most of the information was very helpful, especially in clarifying where the pain originates, and how exercise and activity can help manage the pain, stabilize the joint and improve mood.	One physician indicated the evidence is not there for glucosamine being helpful in managing osteoarthritis pain, and in a later segment the physical therapist indicated that there is evidence glucosamine may be mildly helpful. I felt these contradictory messages confused the issue more than being helpful.	None
Always great to access new information, although very little was actually new to me.	The second section of videos seemed repetitive. I also noted a couple of contradictions among the presenters (one said acupuncture was ineffective, another said it could help). On the whole, too long.	It was all over the map; some very technical, occasionally repetitive, presenters were good.
That activity improves nutrition of cartilage	At some points, program says exercise is beneficial at other points that it contributed to arthritis	No
More tools to manage my arthritis	Nothing	No
	There were conflicting statements	The second module was a little technical for the layman. The male PT with Australian accent

		gave statements that conflicted with the MD from Vermont. The female PT from Canada was excellent.
	A little outdated in the timing	
The explanation of the physiology and progress of arthritis.	There was copnfoicting info regarding efficacy of some things such as glocosamine and accupuncture.	Audio was a bit muted at times and the subtitles werre autogenerated and had minor accuracy issues.
	The very clinical description was overwhelming	
The fact that there are a lot of things we can do as individuals to slow the progression and control the paint of arthritis.	I didn't find anything confusing, but I can see how some might be intimidated by the video that got into the immune system response to arthritis.	I think the quiz in the first module had on of the question responses mis-phrased. The choice was decreased stress on the joint, but the answer had increased stress.
I can keep exercising and not hurt it any more then normal	That it can,Äôt be fixed	I thought there was more that I could do to fix it
It gives me confidence that exercise and weight loss may help with OA quite a bit	Some modules contradicted each other, especially about glucosamine	Nice way to learn more about OA
That I can manage OA withut replacement	That more sctivity may help	No
The understanding that exercise, activity, and movement is necessary and helpful to improve ones condition.	There was a few contradictions throughout the videos with different things to do to improve ones condition (i.e., whether acupuncture was good or not, whether taking glucosamine is worthwhile, etc.)	There were a few videos included at the end that weren't apart of the modules (or I missed something?)
The medical information, though some of it was over my head.	Nothing much, it was fairly straight forward.	This portion of the study was excessively long and time consuming, especially with the videos. Perhaps in the future this could be broken into two separate, consecutive modules and/or provide the information in the videos for those who

		learn better reading material vs. watching videos.
The explanations and diagrams that were presented in layman,Äôs terms that referred to osteoarthritis, as well as how to manage it.	The clinical presentations were, at times, above the level of a lay person.	Worthwhile use of my time
More awareness	Nothing	None
deeper understanding of the causes and learning more about ways to help me cope with OA symptoms	Some film segment appear to be repetitive	A general outline and time involved at the beginning of the session would be helpful.
how to get more active and manage the pain	nothing	none
Good presentations	The last section was difficult to play. It would continually be loading	No
nothing i can apply	answers to many questions were more for medical students or others involved in care	less open ended questions. more true or false
All	None	No
Descriping the mechanics and characteristics of the knee pain	Sometimes an overload of information	I learned a lot and have become more motivated to try the suggested appraochws
mod4, first video	na	helpful
Glad to hear that exercise and activity are not likely to further damage joints.	There was too much repetition between the segments. Some were much better and more helpful than others.	Still hard to understand how to increase exercise and activity when it is so painful. No real discussion of how much/how long one can take nsaids- other than discuss with md of course.
good information		
how movement is good	there was one segment that was too fast in his presentation	
Start exercising	Nothing	No

The multiple ways of dealing with osteoarthritis	One video pointed out benefits of hyaluronic acid injections and glucosamine while another video dismissed it	No
Change mindset T	No straightforward chN	Thank you
That I finally to believe my doctor that working on my thigh/leg muscles will help reduce my knee pai.	The pathology/physiology video was of interest but way too technical	Not sure that the firefighter's personal story at the end did anything to help me with my knee pain. It sure awakened me to 'get it together'
Very informative from different speakers	Nothing	None
That movement actually can help heal, reduce pain.	Waaaay too much info on the medical side. Sometimes contradictions occurred (chondroiton, glucosamine)	It got less interesting as it went along.
The encouragement of exercise	The part where OA was described in medical diagrams and terms	None
That OA pain is manageable, and can be rectified with a) proper mindset b) proper activities / exercise /treatments	In one module, the doctor does not recommend acupuncture (actually is against it). In a later module, the researcher recommends it.	I'd like to be on the mail list/ communication loop for current & future knowledge, discoveries, advancements in the treatment of OA
Yes	No	Thank you for sharing
explaining what it was, why it happens and what you can do.	some had alot of information it was hard to remember all for the quiz, however,can't think of anything else	I am really glad to take part I thought it was helpful would go back over if I had access
all	the one presenter who drew everything was to in depth	no
Exercise is helpful	Repetitive	
The new ways of thinking about arthritis an new ways of dealing with the pain.	Nothing was unhelpful. I have found new information on effects of osteoarthritis and ways of dealing with the pain.	I appreciate the lessons learned in these modules. They have given meincentive to deal with osteoarthritis in better ways.
interested information, applicable to anyone with osteoarthritis	not at all, i like having a transcript with the videos..	none

	Module two was repeated	
Things I knew, weight loss, and physical activity were re enforced	A lot of the language used by presenters	Maybe should show types of exercises that would be beneficial
Interesting relevant topic	The info with the knee and medical terminology was cluttered	Thanks
Knowing that I can manage my pain by getting active	Too much explanation of science terms but it was informative	I take glucosamine so hearing that is not effective maybe question your video as it did not provide an explanation about not helping
mostly reinforcing what i already know	its a good program	i appreciate what i have learned
Everything	Some spots had too big medical words that lost me	Simplify the language
new information	none	great job
I really provided a good explanation of OA and how to manage it.	One section was just a repeat for the same video 3 times	I really liked the added information about was is and is not effective treatment
good advice and how to manage my oestheoarthritis	nothing	no
Repetition of information in slightly different format	Some segments seemed to contradict others. The doctor in one of the segments who had a strong accent was difficult to understand	Use of more diagrams and/or charts
encouragement to increase activity	Too much detail in medical breakdown of what causes artheritis	
Well, honeslty, it made me realize that there is little that can be done that I'm not already doing.	too long, too repetitive.	I got the vibe that "it sucks to be you" and "you really need to finally drop those 20 pounds."
review	some of the videos were repetitive.	none
The information on how exercise feeds the knee joint.	Not really	Treatments that have not been shown to

reinforced some of what i know	A little technical	
It was very beneficial to learn about the progression of ostio arthritis and how much diet can play a vital part in providing a tactic against inflammation.	All were helpful. Some more than others.	Would like to have had more information about how mucle strength (and lack of) specicially support a knee joint. This would include the strategy of what muscles are most valuable and detrimental in managing ostio arthritis
All	No answer	No answer
Ways of managing osteoarthritis	Lots of good information about ostearthritis and how to alleviate the pain	No
learning about OA and what can help	the module explaining the disease was a lot packed in one module.. the guy drawing	i would love specific exercises for my knee OA
Good information	None	No
Videos were good	None	Little too long each module
It helps cultivate a positve attitude that you can do something to help your OA.	Nothing was confusin. I've researched on my own.	Not at this time.
It was all helpful	Nothing	No
The module about the science of arthritis was very interesting, everything else I already knew	Nothing	No
it made us aware of the importance of doing the right exercise.	one video stated Supplementary of glucosamine is not recommended but one video said it helped even though it's minor.	one part of video presented by the physical therapist (2nd module) contains repetitive info of its 2nd and 3rd parts of video. also some suggestions from a doctor of the first module's contradictive to the suggestions from the physical therapist of the second module.

Learning there were more parts of the knee than I thought/remember. Osteoarthritis can cause fatigue.	It was hard to listen to the presentations. The second program the quality of the video wasn't as good as the others.	With the exception of the firefighter, the presentation were dull. While the information was useful, I really wanted to listen, but found I wanted them to be over.
The examples were very good.	The drawings in module 2 were too too detailed.	Module 2 was my least favority
It covered a lot of scenarios.	Nothing	No
New information learned	It is a good program	
good science information; good anatomy explanation	nothing	nope
Learning that proper exercise shouldn't promote joint degeneration.	The 2nd module (the one with the hand drawn images) was too full of new terminolgy to easily follow.	No
The detailed explanation of the science of osteoarthritis. The x rays and drawings were interesting and helped me to understand the factors affecting OA	two presenters did present differing views. while supplements do not have a significant amount of research, some patients do get relief from glucosamine or condroitin	I would try to limit the videos to 5 min. I started to wander when they went longer
The in-depth look into arthritis	The breakdown of the knee joints	None
learned new things about supplements and the need for more daily activities	some things were repeated to much	glad i was able to take part and learn new information about my OA
Understanding how the cartilage and joints need exercise	some of the terminology when describing OA	Interested in how to start a program to reduce pain from OA
detailed information	nothing	no
The focus on physical activity as a way to help with OA	Nothing	Too long. There was a lot of repetition.
I found the lectures with the diagrams interesting	The doctor from Canada had little to contribute in comparison to the other Doctors	It was long, but very informative
There was lots of good information reinforcing what I	Some of the presenters advocated contradictory	It would be helpful to have concrete examples of how to

had already learned, and it's good to know that I may be able to slow the progression of the disease by following their recommendations.	treatment options, for example, recommending acupuncture. Some things they said they would discuss, eg. braces, topical ointments, were not discussed.	implement recommended practices in real life, how to track progress, and how to know when "enough is enough". Some treatments, for example, are impractical--who has 20 minutes per hour to sit around with an ice pack? Not me!
The repetitive nature of the information is helpful in emphasizing a mindset. For example the importance of exercise and strength training, icing and activity and losing weight.	The one segment was extremely detailed. I thought it more appropriate for someone in the medical field than a patient. Also some of the information was contradictory. An example was the first segment said that glucosomine was not proven to be helpful, and cleaning up the joint did not provide a benefit. In the most recent YouTube video the presenter said that glucosomine has been proven to provide pain relief and suggested that cleaning up the joint is helpful.	The woman presenter on You Tube has an extremely monotonous tone and presentation.
All of it especially how the knee works and is effective by oa	Nothing i understood everything	Great program and very informative
